# MARK3 promotes host antiviral defense by driving GEF-H1–mediated TBK1 activation

**DOI:** 10.1371/journal.ppat.1014701

**Published:** 2026-10-27

**Authors:** Xingmei Qi, Xue Zhu, Wenbiao Peng, Luoxue Hua, Xinru Xie, Chuncan Si, Meimei Lan, Zifan Zhuang, Siyi Ding, Ting Xu, Junjie Li, Yundi Zhang, Yifan Song, Yiping Yang, Yixue Cheng, Tao Guo, Linchao Li, Kezhen Wang

**Affiliations:** 1 Jiangsu Key Laboratory of Infection and Immunity, Institutes of Biology and Medical Sciences, Soochow University, Suzhou, Jiangsu, China; 2 Department of Microbiology and Biotechnology, College of Life Sciences, Anhui Medical University, Hefei, China; 3 Department of General Surgery, The First Affiliated Hospital of Anhui Medical University, Hefei, China; 4 Anhui Public Health Clinical Center, Hefei, China; 5 School of Biological and Environmental Engineering, Jingdezhen University, Jingdezhen, China; University of Kentucky College of Medicine, UNITED STATES OF AMERICA

## Abstract

Innate immunity constitutes the body’s first line of defense against pathogenic challenge, serving not only as a physical and cellular bulwark that rapidly curbs microbial proliferation but also as the essential rheostat that orchestrates downstream adaptive immune responses. Here, we identified MARK3, a serine/threonine protein kinase of the conserved MARK family, as a crucial regulator of innate antiviral immunity that promotes type I interferon (IFN-I) production in a kinase-dependent manner. Using both *in vitro* and *in vivo* models, we demonstrate that MARK3 exerts broad-spectrum antiviral activity via a signaling axis involving GEF-H1 and TBK1. Mechanistically, MARK3 directly interacts with GEF-H1 and phosphorylates it at Ser645. This phosphorylation event potentiates the activation of TBK1, leading to enhanced IRF3 phosphorylation. Activated IRF3 then translocates to the nucleus, where it triggers potent IFN-I induction and the downstream expression of interferon-stimulated genes (ISGs). Our study establishes MARK3 as an important antiviral kinase and defines the MARK3–GEF-H1–TBK1 axis as a novel signaling pathway driving interferon-mediated antiviral defense.

## Author summary

The innate immune system serves as the body’s first line of defense against viral pathogens, acting to curb infection at its earliest stages. In this study, we identified MARK3 as a candidate GEF-H1–interacting kinase and further showed that it enhances innate antiviral immunity in a kinase-dependent manner. Using both in vitro and in vivo models, we demonstrate that MARK3 binds to and phosphorylates GEF-H1 at Ser645, which in turn promotes TBK1 and IRF3 phosphorylation, driving robust activation of type I interferons to reinforce the host antiviral response.

## Introduction

The innate immune system constitutes the body’s first line of defense against viral infection, employing pattern recognition receptors (PRRs) to detect viral pathogen-associated molecular patterns (PAMPs), particularly viral nucleic acids [1,2]. Specialized cytoplasmic sensors play distinct roles in this detection: RIG-I and MDA5 recognize viral RNA, while cGAS serves as the primary DNA sensor [3,4]. Upon viral recognition, adapter proteins such as MAVS and STING recruit the kinases TBK1 and IKKε to initiate downstream signaling [5–7]. The TBK1-IKKε complex subsequently phosphorylates and activates transcription factor IRF3, promoting its nuclear translocation and inducing type I interferon (IFN-α/β) production and interferon-stimulated gene (ISG) expression, thereby establishing a robust antiviral state [8].

Guanine nucleotide exchange factor H1 (GEF-H1), a microtubule-associated RhoGEF, regulates various cellular processes including epithelial barrier integrity, cell cycle progression, motility, polarization, and leukemic cell differentiation [9]. Notably, GEF-H1 also enhances inflammatory cytokine production, facilitates intracellular pathogen clearance, and contributes to antiviral defense by promoting TBK1-mediated interferon activation in macrophages [10,11]. However, the regulatory mechanisms controlling GEF-H1 function extend beyond microtubule association and involve complex phosphorylation-dependent regulation and dynamic protein-protein interactions [12–18]. To further identify the kinases that manipulate GEF-H1 to exert antiviral effects, we stimulated HUVECs with Sendai virus (SeV) and performed immunoprecipitation-mass spectrometry (IP-MS) to identify GEF-H1-interacting proteins. Among these, we identified Microtubule Affinity-Regulating Kinase 3 (MARK3) as a key candidate.

MARK3, a serine/threonine protein kinase highly conserved within the MARK family [19], plays crucial roles in regulating microtubule dynamics and cell migration [20]. Beyond these functions, MARK3 phosphorylates diverse substrates and participates in multiple signaling pathways, including cell cycle regulation, Ras signaling, cAMP-PKA pathway, JNK signaling, and Notch signaling [21–24]. Previous studies have shown that MARK3 phosphorylates GEF-H1 at Ser151 to establish cell polarity [25]. Bioinformatics analyses suggest that MARK3 interacts with SARS-CoV-2 proteins and may contribute to viral suppression by modulating innate immune cell populations [26,27].

In this study, we identify MARK3 as a novel positive regulator of the antiviral interferon response. We demonstrate that MARK3 activates TBK1 and amplifies innate immunity by interacting with and phosphorylating GEF-H1 at Ser645, revealing a previously unrecognized signaling mechanism in antiviral defense.

## Results

### The antiviral role of MARK3 *in vitro*

Since MARK3 was identified as a candidate GEF-H1-interacting protein (S1 Table), we first investigated its association with viral infection. In a previous study, a systematic characterization of protein quantitative trait loci (pQTLs) across the phenome identified MARK3 as a putative viral interaction partner that affects immune response, linking it to protein–drug–disease interactions [27]. Here, we performed Module Membership–Gene Significance (MM–GS) correlation analysis, which demonstrated that genes associated with H1N1 or VSV infection are generally hub genes within their respective infection-related modules (S1A Fig). Using a dual-dimensional screening strategy, we also identified MARK3 as one of the 40 key hub genes involved in both H1N1 and VSV infection processes (S1B Fig).

To confirm the antiviral role of MARK3, we first infected HUVECs with Vesicular Stomatitis Virus (VSV) and Herpes Simplex Virus type 1 (HSV-1). We found that overexpression of MARK3 consistently suppressed the replication of both viruses, as evidenced by markedly lower levels of viral genome, reduced viral protein expression, and decreased virus titers compared with control cells (Fig 1A and 1B). Furthermore, overexpression of MARK3 significantly reduced the fluorescence intensity of VSV-GFP in HEK293T cells (S2A Fig). The replication of VSV and HSV-1 was similarly restricted in HEK293T cells with MARK3 ectopic expression (S2B and S2C Fig). Conversely, MARK3 knockdown significantly enhanced the replication of both VSV and HSV-1 compared to controls in HEK293T cells (S2D and S2E Fig) or in HUVECs (Fig 1C and 1D).

**Fig 1:**
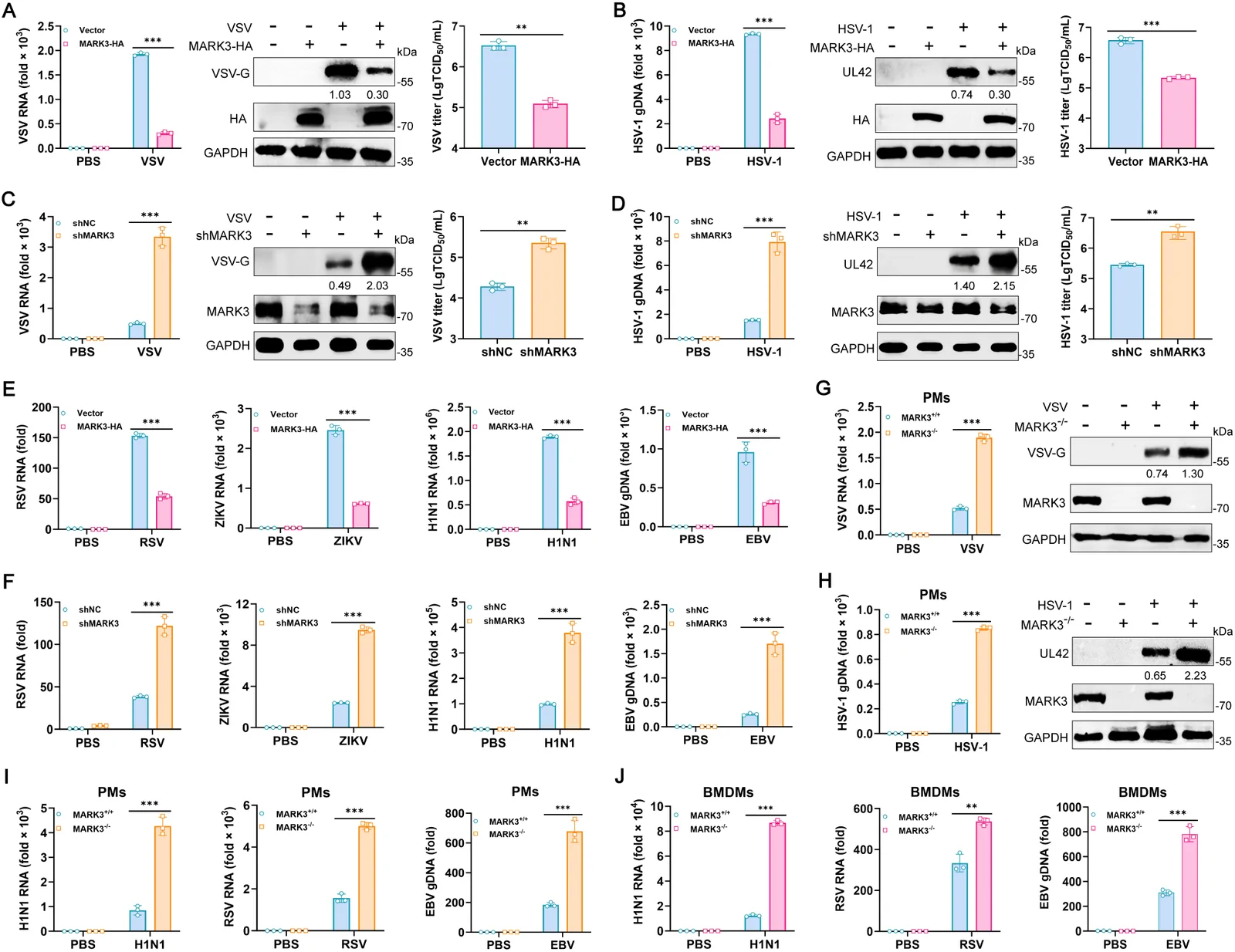
MARK3 exhibits broad-spectrum antiviral activity. **(A, B)** Viral genome copies, protein levels, and progeny virus titers were measured in VSV- (A) and HSV-1- (B) infected HUVECs overexpressing MARK3 at 12 and 24 hours post-infection (hpi), respectively. **(C, D)** Viral genome copies, protein levels, and progeny virus production were assessed in VSV- (C) and HSV-1- (D) infected HUVECs expressing MARK3 shRNA at 12 and 24 hpi, respectively. (**E, F**) mRNA expression levels of RSV, ZIKV, H1N1, and EBV were evaluated in HUVECs transfected with MARK3-HA (E) or MARK3 shRNA (F) for 24 h. **(G, H)** Viral mRNA and protein levels were analyzed in mouse MARK3^-/-^ and MARK3^+/+^ PMs that were either mock-infected or infected with VSV (G) or HSV-1 (H) at the indicated time points. **(I, J)** Replication of H1N1, RSV, and EBV were examined in MARK3^-/-^ PMs (I) and BMDMs **(J)**. Data represent mean ± SEM of 3 independent experiments. Statistical analysis was performed using two-way ANOVA followed by Welch’s t-test with Holm correction. *P < 0.05, **P < 0.01, ***P < 0.001; NS, not significant.

To further determine whether MARK3 exerts broad-spectrum antiviral activity, we extended to test its effect against respiratory syncytial virus (RSV), Zika virus (ZIKV), Influenza A virus (H1N1), and Epstein–Barr virus (EBV). Ectopic expression of MARK3 significantly reduced viral genome levels across all viruses tested (Fig 1E), while MARK3 knockdown consistently enhanced infection by each (Fig 1F).

Having established MARK3’s antiviral function in cell lines, we sought to validate these findings in a physiologically relevant context by generating MARK3 knockout (MARK3^-/-^) mice via CRISPR-Cas9 and isolating primary peritoneal macrophages (PMs) or bone marrow-derived macrophages (BMDMs) from homozygous offspring and wild-type (MARK3^+/+^) controls. Infection of these PMs with VSV and HSV-1 demonstrated that MARK3 deficiency enhanced viral replication, as both viral genome and protein levels were significantly elevated in MARK3^-/-^ cells compared to MARK3^+/+^ controls (Fig 1G and 1H). In addition, the replication of H1N1, RSV and EBV was enhanced in both MARK3^-/-^ PMs and BMDMs (Fig 1I, J). Taken together, these results establish MARK3 as a key mediator of a broad-spectrum antiviral response.

### MARK3 promotes IFN-β activation

Having established the cell-intrinsic antiviral role of MARK3, we next sought to determine whether it functions through the interferon (IFN) signaling pathway. Using dual-luciferase reporter (DLR) assays in HEK293T cells, we found that MARK3 overexpression enhanced the promoter activity of both IFN-β and ISRE upon stimulation with the RIG-I N-terminal domain (RIG-IN) or SeV (Fig 2A). Consistent with this, MARK3 potentiated the expression of IFN-β mRNA (Fig 2B) as well as three representative interferon-stimulated genes (ISGs)—IFIT1, ISG15, and CIG5—following SeV or Poly(I:C) challenge in HUVECs (Fig 2C). Given that IFN-β activation depends on TBK1 and IRF3 phosphorylation—a process that promotes IRF3 dimerization and nuclear translocation [28]—we next examined the role of MARK3 in these signaling events. Consistent with this premise, ectopic MARK3 expression in SeV-stimulated HUVECs potently enhanced TBK1 and IRF3 phosphorylation (Fig 2D).

**Fig 2:**
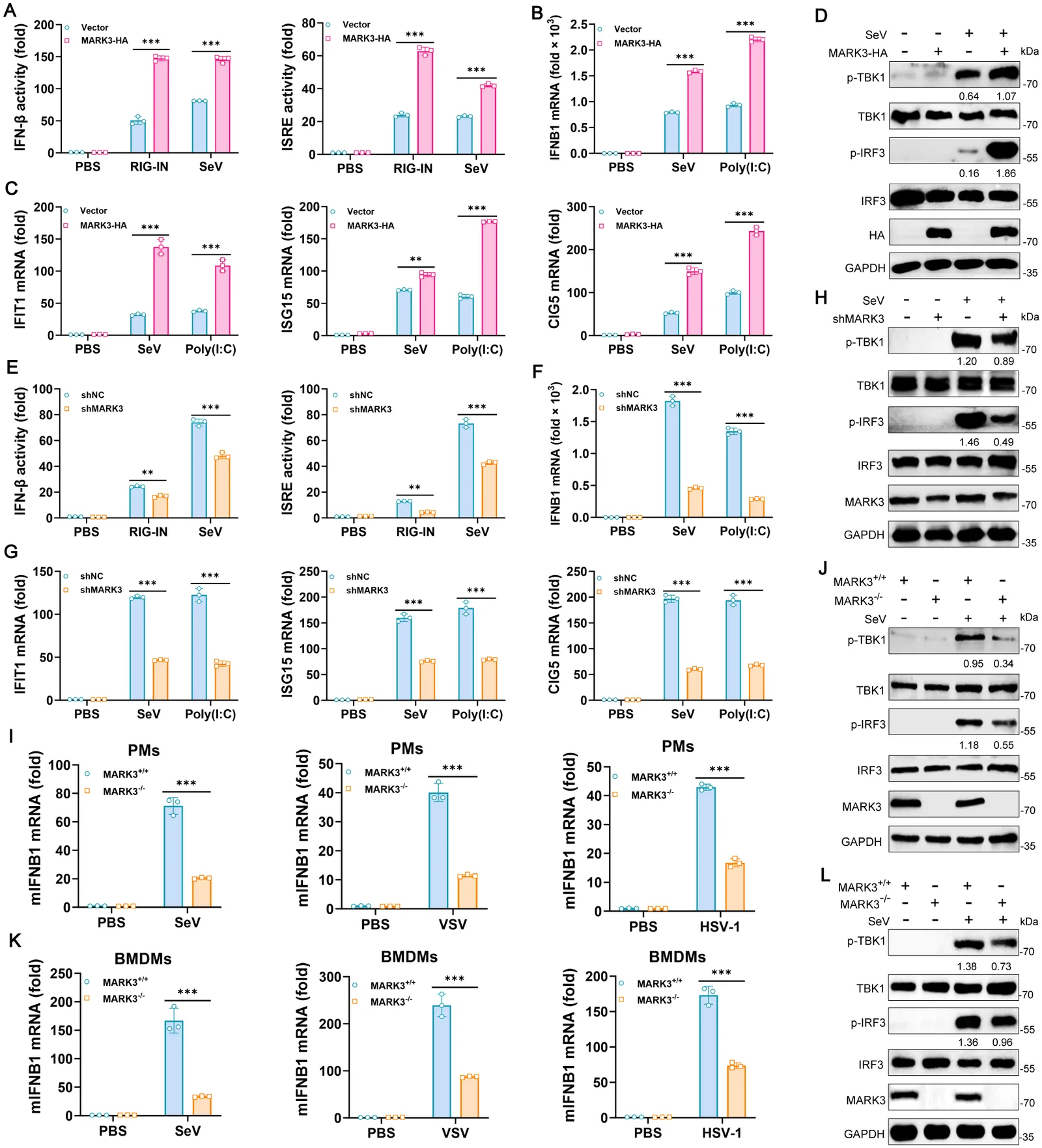
MARK3 enhances the expression of IFN-β and ISGs. **(A)** Luciferase reporter activity driven by the IFNB1 promoter (IFN-β-Luc) or an interferon-stimulated response element (ISRE-Luc) in HEK293T cells transfected with empty vector or MARK3-HA, along with an expression plasmid for RIG-IN or following SeV stimulation. (**B, C**) mRNA expression levels of IFN-β **(B)**, as well as IFIT1, ISG15, and CIG5 **(C)**, in HUVECs overexpressing MARK3 after stimulation with SeV or Poly(I:C). **(D)** Immunoblot analysis of phosphorylated (p-) and total TBK1 and IRF3 in HUVECs overexpressing MARK3, with or without SeV stimulation. **(E)** IFNB1 and ISRE promoter activities in MARK3-silenced HUVECs upon RIG-IN transfection or SeV stimulation. (**F, G**) mRNA levels of IFN-β **(F)**, along with IFIT1, ISG15, and CIG5 **(G)**, in MARK3-silenced HUVECs stimulated with SeV or Poly(I:C). **(H)** Phosphorylation status of TBK1 and IRF3 in MARK3-silenced HUVECs with or without SeV stimulation. **(I)** The mRNA levels of IFN-β in MARK3^-/-^ PMs stimulated with SeV, VSV or HSV-1. **(J)** The phosphorylation of TBK1 and IRF3 in SeV-stimulated PMs with or without MARK3 knockout. **(K)** The expression of IFN-β in MARK3^-/-^ BMDMs with SeV, VSV, or HSV-1 stimulation. **(L)** The phosphorylation of TBK1 and IRF3 in MARK3^-/-^ BMDMs stimulated with SeV. Data represent mean ± SEM of 3 independent experiments. Statistical analysis was performed using two-way ANOVA followed by Welch’s t-test with Holm correction. *P < 0.05, **P < 0.01, ***P < 0.001; NS, not significant.

Conversely, MARK3 knockdown by shRNA attenuated IFN-β and ISRE promoter activation induced by RIG-IN or SeV (Fig 2E). It also reduced the expression of IFN-β (Fig 2F) and the three selected ISGs upon SeV or Poly(I:C) stimulation in HUVECs (Fig 2G). Accordingly, MARK3 depletion impaired TBK1 and IRF3 phosphorylation in SeV-treated HUVECs (Fig 2H). In addition, MARK3 also enhances IFN activation in HEK293T cells (S3 Fig). These data collectively indicate that MARK3 plays an essential role in potentiating the IFN-mediated immune response.

To further validate the role of MARK3 in IFN induction in a physiologically relevant setting, we isolated primary macrophages from MARK3^+/+^ and MARK3^-/-^ mice and stimulated them with SeV, HSV-1, or VSV. In all cases, IFN-β expression was significantly lower in MARK3^-/-^ PMs than in MARK3^+/+^ controls (Fig 2I). Additionally, SeV-induced phosphorylation of TBK1 and IRF3 was markedly dampened in MARK3^-/-^ PMs (Fig 2J). Similar results were observed in MARK3^-/-^ BMDMs (Fig 2K and 2L). Collectively, these findings demonstrate that MARK3 mediates broad antiviral activity by potentiating the IFN-β response.

### Kinase activity is necessary for MARK3’s antiviral function

MARK3 contains an N-terminal serine/threonine kinase domain, a central ubiquitin-associated domain, and a C-terminal kinase-associated domain [20]. Having determined that MARK3 potentiates the IFN-mediated immune response, we next sought to investigate whether this function depends on its kinase activity. Given that phosphorylation of Thr211 in the activation loop is essential for MARK3 kinase activity [20,29], we generated a kinase-dead T211A mutant, which has minimal impact on the overall protein structure (Fig 3A). In HEK293T cells stimulated by RIG-IN, MARK3 wild-type significantly enhanced the promoter activity of both IFN-β and ISRE, whereas the T211A mutant failed to do so (Fig 3B). Similarly, only wild-type MARK3 potentiated the mRNA levels of IFN-β and three representative ISGs upon SeV or Poly(I:C) stimulation in HUVECs (Fig 3C and 3D). At the protein level, the T211A mutant impaired the phosphorylation of TBK1 and IRF3, as well as the formation of IRF3 dimers, in response to SeV infection (Fig 3E).

**Fig 3:**
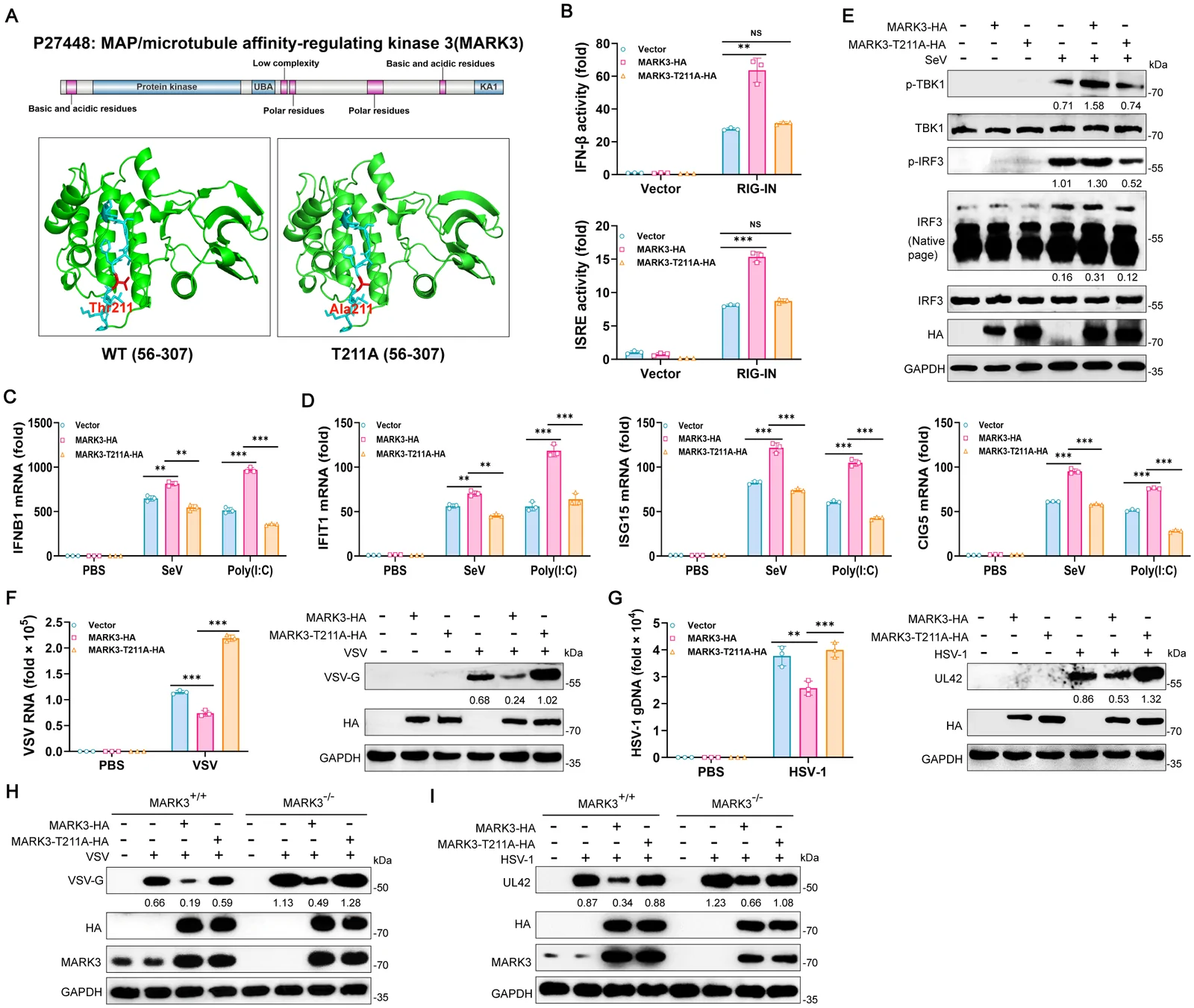
Kinase activity is required for the antiviral function of MARK3. **(A)** Schematic representation and structural model of MARK3 WT and its T211A mutant. **(B)** IFNB1 or ISRE promoter activity in HEK293T cells transfected with MARK3‑HA WT or the T211A mutant. (**C, D**) mRNA levels of IFN‑β **(C)**, as well as IFIT1, ISG15, and CIG5 **(D)**, in HUVECs transfected with MARK3 WT or the T211A mutant and stimulated with SeV or Poly(I:C). **(E)** Immunoblot analysis of phosphorylated (p‑) and total TBK1 and IRF3, and of monomeric (Mono‑) and dimeric (Dimer‑) IRF3 (by native PAGE), in HUVECs transfected with MARK3 WT or the T211A mutant, with or without SeV stimulation. **(F, G)** Viral mRNA and protein levels of VSV (F) or HSV‑1 (G) in HUVECs transfected with MARK3 WT or the T211A mutant. (**H, I**) Viral protein levels of VSV (H) or HSV-1 (I) transfected with MARK3 WT or the T211A mutant in MARK3^-/-^ HEK293T cells. Data represent mean ± SEM of 3 independent experiments. Statistical analysis was performed using two-way ANOVA followed by Welch’s t-test with Holm correction. *P < 0.05, **P < 0.01, ***P < 0.001; NS, not significant.

We further assessed the functional relevance of MARK3 kinase activity in restricting viral replication. In HUVECs, both viral mRNA and protein levels of VSV and HSV-1 were strongly suppressed in cells expressing MARK3 WT, but not in those expressing the T211A mutant (Fig 3F and 3G). Furthermore, the inability of the T211A mutant to restrict the replication of these two viruses was corroborated in MARK3^-/-^ HEK293T cells (Fig 3H and 3I). Taken together, these results demonstrate that the kinase activity of MARK3 is indispensable for its ability to activate the IFN response and confer antiviral immunity.

### MARK3 plays a critical role in antiviral defense *in vivo*

To assess the physiological relevance of our *in vitro* findings, we evaluated the antiviral role of MARK3 in a murine model. Following VSV infection, MARK3^-/-^ mice exhibited a significant decrease in IFN-β expression across multiple organs—including the heart, liver, spleen, lung, and kidney—compared to wild-type controls (Fig 4A). This compromised IFN response correlated with significantly enhanced viral replication, as demonstrated by elevated viral mRNA and protein levels across all examined organs in the MARK3^-/-^ cohort (Fig 4B and 4C). Histopathological analysis further revealed more severe lung tissue damage in VSV-infected MARK3 knockout mice (Fig 4D). Furthermore, IFN-β transcript levels in the blood and protein levels in the serum were broadly downregulated in the knockout animals, underscoring the critical role of MARK3 in antiviral immunity in vivo (Fig 4E). Consistent with this impaired defense, viral RNA levels in the blood were significantly higher in MARK3^-/-^ mice than in MARK3^+/+^ controls (Fig 4F). Finally, in survival analyses, MARK3^-/-^ mice exhibited significantly higher mortality upon infection with VSV (Fig 4G).

**Fig 4:**
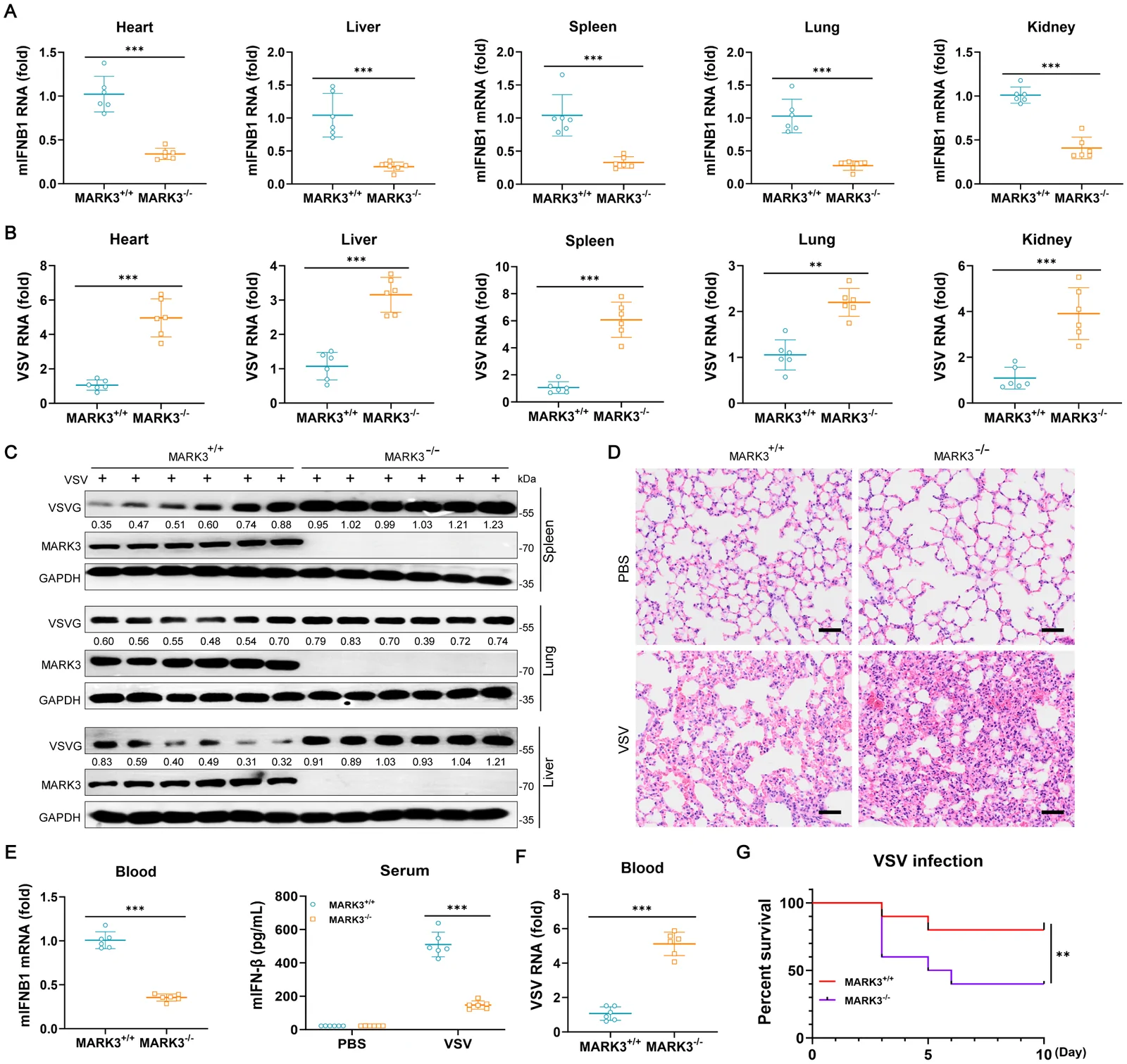
MARK3 is essential for antiviral immunity in vivo. (**A, B**) qPCR analysis of IFN-β mRNA (A) and viral RNA (B) in the indicated organs from VSV-infected MARK3^+/+^ and MARK3^-/-^ mice (n = 6). **(C)** Immunoblot analysis of viral protein levels in spleen, lung, and liver lysates from VSV-infected MARK3^+/+^ and MARK3^-/-^ mice (n = 6). **(D)** Hematoxylin and eosin **(H&E)**-stained lung sections from PBS- or VSV-treated mice (24 hpi.). Scale bar: 100 μm. **(E)** IFN-β levels were measured in the blood (RT-PCR) and serum (ELISA) of MARK3^+/+^ and MARK3^-/-^ mice (n = 6 per group) at 24 h after intraperitoneal injection of PBS or VSV (5 × 10^8^ PFU/mouse). **(F)** Quantification of Viral RNA in the blood of VSV-infected MARK3^+/+^ and MARK3^-/-^ mice (n = 6). **(G)** Survival curves of MARK3^+/+^ and MARK3^-/-^ mice (n = 10) following intraperitoneal VSV infection (1 × 10^8^ PFU/mouse). Data are presented as mean ± SD from n = 6 biologically independent mice and are representative of three independent experiments. Statistical analysis was performed using two-way ANOVA followed by Welch’s t-test with Holm correction (A-F) and Log-rank test **(G)**. NS, not significant; *P < 0.05, **P < 0.01, ***P < 0.001.

To further investigate the role of MARK3 in adaptive immunity, we evaluated immune cell development in mice by quantifying the proportions of B cells, T cells, dendritic cells (DCs), and myeloid cells in the spleen and lymph nodes. The results indicated that MARK3 deficiency did not significantly impact the development of these immune cell populations (S4 Fig). Collectively, these in vivo data demonstrate that MARK3 is essential for mounting a protective type-I interferon-mediated antiviral response at the organismal level.

### MARK3 mediates antiviral immunity via GEF-H1

We observed that GEF-H1 expression was upregulated in HUVECs following stimulation with SeV or Poly(I:C) (S5A and S5B Fig), and that the replication of both VSV and HSV-1 was effectively restricted by GEF-H1 (S5C and S5D Fig). However, treatment with nocodazole, a microtubule inhibitor, dampened both the interaction between TBK1 and GEF-H1 and the downstream phosphorylation of TBK1 and IRF3 (S5E and S5F Fig). Moreover, overexpression of GEF-H1 significantly enhanced IFN-β expression and markedly suppressed VSV replication in wild-type HEK293T cells, but this effect was abolished in TBK1-knockout (TBK1^-/-^) cells. (S5G Fig). These findings confirm that GEF-H1 functions as a microtubule-associated guanine nucleotide exchange factor that physically links microtubule dynamics to RIG-I-mediated viral RNA sensing [10].

Since MARK3 is recruited by GEF-H1 (S1 Table), we further explored the relationship between these two proteins. After confirming the interaction between MARK3 and GEF-H1 via co-immunoprecipitation (Co-IP) of the ectopically expressed proteins both in HEK293T cells and HUVECs (Fig 5A), as well as at the endogenous level in mouse PMs with or without SeV stimulation (Fig 5B), we performed GST pulldown assays to demonstrate a direct interaction between the two proteins (Fig 5C). Furthermore, we observed that both MARK3 and GEF-H1 remained localized in the cytoplasm regardless of SeV or HSV-1 infection (Fig 5D). Confocal assays also confirmed that TBK1, GEF-H1, and MARK3 each colocalized with β-tubulin in the cytoplasm, and that this colocalization was enhanced following SeV stimulation (Fig 5E).

**Fig 5:**
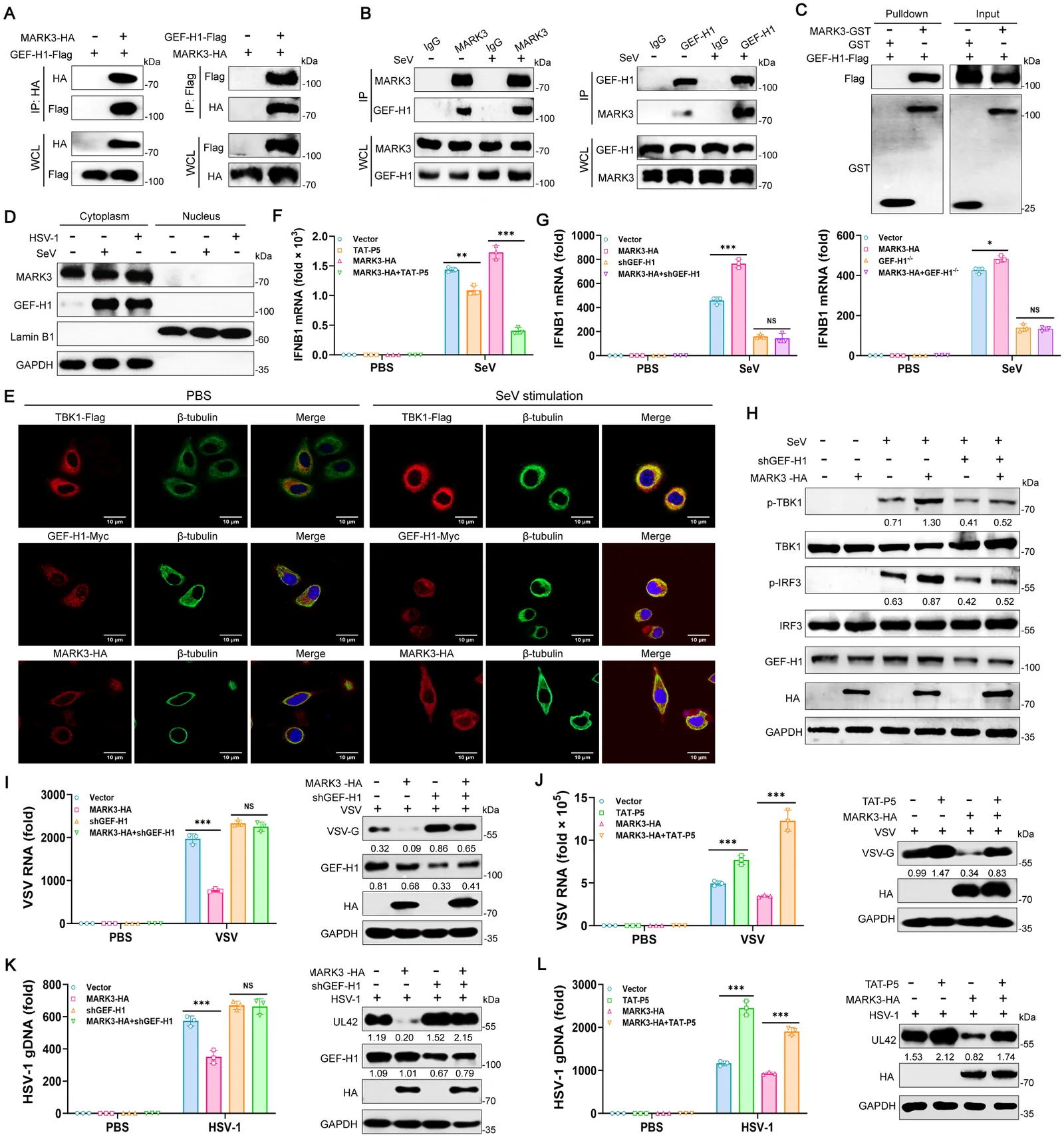
MAKR3 exerts antiviral activity through GEF-H1. **(A)** Co-IP of lysates from HEK293T cells expressing GEF-H1-Flag with or without MARK3-HA (Left). Reciprocal Co-IP of lysates from HUVECs transfected MARK3-HA in the presence or absence of GEF-H1-Flag (Right). Immunoprecipitation was performed using anti-HA or anti-Flag antibody, respectively. **(B)** Endogenous Co-IP in mouse PMs with or without SeV stimulation using an anti-MARK3 antibody. **(C)** GST-pulldown assays with MARK3-GST in the presence or absence of purified GEF-H1-Flag. **(D)** Protein levels of MARK3 and GEF-H1 in the nuclear and cytoplasmic fractions of SeV-stimulated HUVECs. Lamin B1 and GAPDH served as nuclear and cytoplasmic markers, respectively. **(E)** Cofocal microscopy images of HeLa cells expressing TBK1-Flag, GEF-H1-Myc or MARK3-HA with or without SeV stimulation, Anti-Flag, anti-Myc, anti-HA and anti-β-tubulin antibodies were used to detect the indicated proteins, and nuclei were counterstained with DAPI. **(F)** IFN-β mRNA expression in SeV-stimulated HEK293T cells following ectopic MARK3 expression or TAT-P5 treatment. **(G**) IFN-β mRNA levels in SeV-stimulated HUVECs with GEF-H1 knockdown or GEF-H1^-/-^ HEK293T cells, with or without ectopic expression of MARK3. **(H)** Phosphorylation of TBK1 and IRF3 in HUVECs transfected with MARK3, with or without GEF-H1 knockdown. **(I, J)** Viral mRNA and protein levels in VSV-infected HEK293T cells with GEF-H1 knockdown (I) or HUVECs after TAT-P5 treatment **(J)**. **(K, L)** HSV-1 mRNA and protein levels in HEK293T cells following GEF-H1 knockdown (K) or HUVECs treatment with the GEF-H1 inhibitor TAT-P5 **(L)**. Data represent mean ± SEM of 3 independent experiments. Statistical analysis was performed using two-way ANOVA followed by Welch’s t-test with Holm correction. *P < 0.05, **P < 0.01, ***P < 0.001; NS, not significant.

Subsequently, we treated HEK293T cells with TAT-P5, a specific inhibitor of GEF-H1 [30], in the presence or absence of SeV stimulation. The results demonstrated that TAT-P5 treatment significantly dampened the upregulation of IFN-β expression (Fig 5F). This finding was consistent with observations in GEF-H1-knockdown HUVECs and GEF-H1-knockout HEK293T cells (Fig 5G). In addition, MARK3-induced phosphorylation of TBK1 and IRF3 was attenuated in GEF-H1-knockdown HUVECs (Fig 5H). Functionally, MARK3’s ability to suppress VSV mRNA and protein levels was lost upon GEF-H1 knockdown in HEK293T cells (Fig 5I) or following TAT-P5 treatment in HUVECs (Fig 5J). Similar results were observed in HSV-1-infected cells (Fig 5K and 5L). Together, these findings demonstrate that MARK3 exerts its antiviral activity through a functional interaction with GEF-H1.

### MARK3 phosphorylates GEF-H1 at Ser645 site

To pinpoint functionally relevant phosphorylation sites on GEF-H1 in antiviral signaling, we conducted IP-MS assays in SeV-stimulated HUVECs overexpressing GEF-H1-Flag, identifying six sites (S151, T152, S645, T679, S691, S696). Functional analysis of alanine substitution mutants showed that all except S645A enhanced SeV-induced IFN-β expression (S6A Fig) and inhibited VSV replication (S6B Fig) to a similar extent as the wild-type protein, implicating S645 as a key regulatory site. Co-IP assays further confirmed that the Ser645 mutant of GEF-H1 partly dampened the interaction between itself and TBK1 (S6C Fig).

To further validate the role of S645 phosphorylation, we constructed phosphomimetic mutants S645D and S645E. In HEK293T cells stimulated with RIG-IN or SeV, the S645A mutant failed to enhance IFN-β promoter activity, whereas both S645D and S645E significantly potentiated it (S6D Fig). Similarly, only the phosphomimetic mutants promoted TBK1 and IRF3 phosphorylation (S6E Fig) and increased mRNA levels of IFN-β and ISGs in HUVECs (S6F Fig). Furthermore, replication of both VSV (S6G Fig) and HSV-1 (S6H Fig) was suppressed in cells expressing GEF-H1 WT, S645D, or S645E, but not the S645A mutant, confirming that phosphorylation at S645 is essential for GEF-H1–mediated antiviral immunity.

To determine whether MARK3 functions as the upstream kinase for GEF-H1 phosphorylation, we assessed the impact of MARK3 on GEF-H1 activation. While the expression of IFN-β and ISGs was significantly enhanced by GEF-H1 overexpression, this effect was markedly impaired in MARK3-knockdown cells, regardless of whether they were stimulated with SeV or Poly(I:C) (Fig 6A and 6B). Indeed, MARK3 overexpression robustly increased the overall phosphorylation level of GEF-H1-Flag upon SeV stimulation (Fig 6C). Among the six phosphorylation site mutants, only S645A abolished MARK3-induced phosphorylation (Fig 6D), indicating that S645 is the primary residue targeted by MARK3. SeV infection alone induced GEF-H1 phosphorylation at S645, and this was further enhanced by MARK3 in a dose-dependent manner (Fig 6E). Critically, wild-type MARK3, but not the kinase-dead T211A mutant, significantly increased phosphorylation of both overexpressed (Fig 6F) and endogenous (Fig 6G) GEF-H1 at S645 in SeV-stimulated HUVECs. Taken together, these results demonstrate that MARK3 phosphorylates GEF-H1 specifically at Ser645 to potentiate interferon activation and antiviral defense.

**Fig 6:**
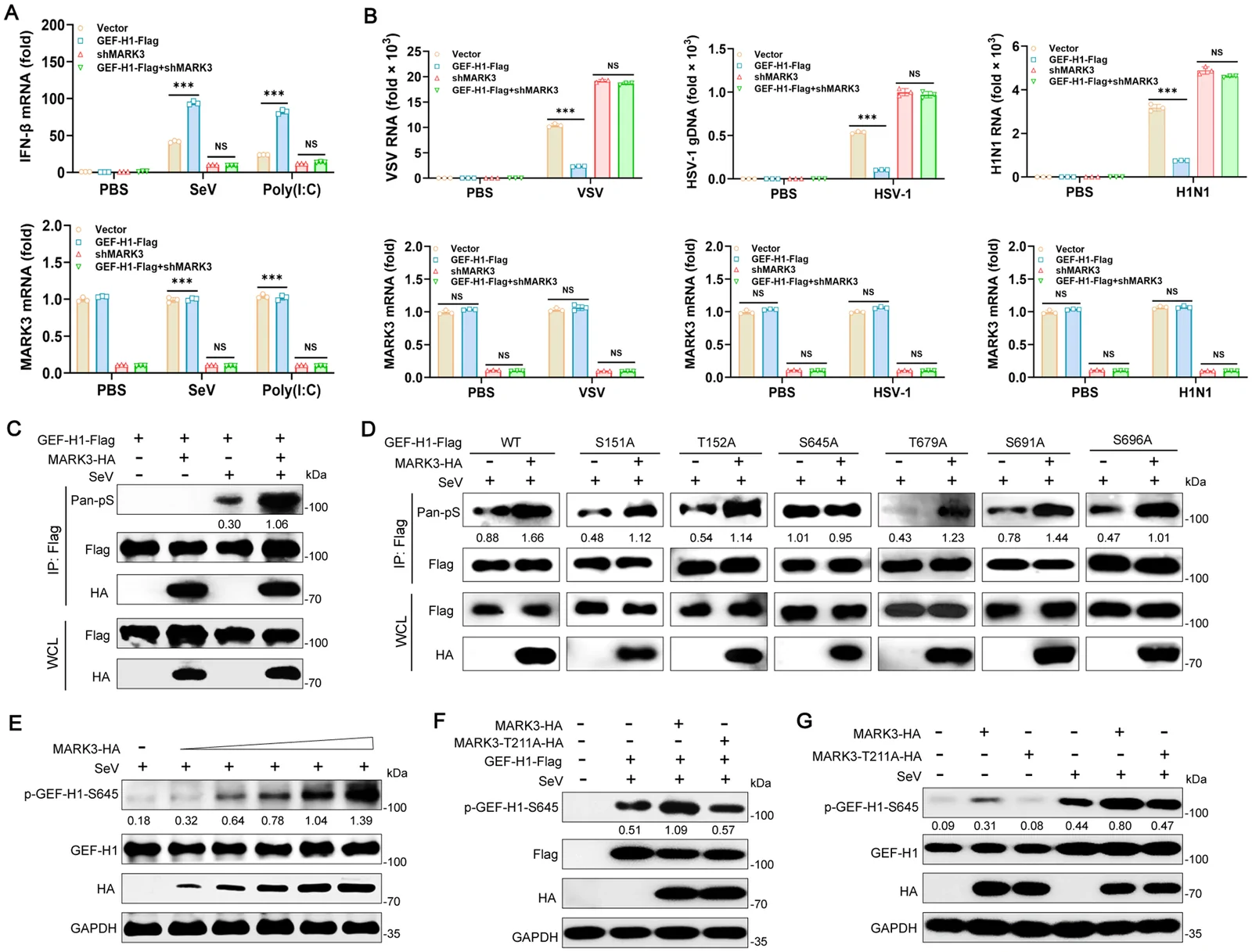
MARK3 phosphorylates GEF-H1 at Ser645 site. (**A**) mRNA levels of IFN-β and MARK3 in SeV- or Poly(I:C)-stimulated HUVECs following GEF-H1 overexpression or MARK3 knockdown. **(B)** Viral RNA levels in GEF-H1-overexpressing HUVECs with or without MARK3 knockdown. **(C)** SeV-stimulated HUVECs were transfected with GEF-H1-Flag alone or with MARK3-HA. Anti-Flag immunoprecipitates were immunoblotted with a pan-phosphoserine (pan-pS) antibody to assess GEF-H1 phosphorylation. **(D)** HUVECs expressing WT or each mutant GEF-H1-Flag with or without MARK3-HA, were stimulated with SeV. GEF-H1 phosphorylation was analyzed in anti-Flag immunoprecipitates using a pan-pS antibody. **(E)** Ser645 phosphorylation of GEF-H1 in SeV-stimulated HUVECs transfected with increasing doses of MARK3-HA. **(F)** Ser645 phosphorylation of GEF-H1 in HUVECs co-expressing GEF-H1 with either MARK3 WT or the kinase-dead T211A mutant. **(G)** Endogenous GEF-H1 phosphorylation at Ser645 in HUVECs expressing MARK3 WT or the T211A mutant, with or without SeV stimulation. Data represent mean ± SEM of 3 independent experiments. Statistical analysis was performed using two-way ANOVA followed by Welch’s t-test with Holm correction. *P < 0.05, **P < 0.01, ***P < 0.001; NS, not significant.

### MARK3 exerts an antiviral effect via GEF-H1–TBK1 axis

To elucidate the mechanism by which MARK3 regulates the interferon pathway, we systematically investigated its functional position within the antiviral signaling network. We overexpressed MARK3 in HEK293T cells together with key signaling components—RIG-IN, MAVS, TBK1, IKKε, constitutively active IRF3 (IRF3/5D), or cGAS/STING. MARK3 enhanced the promoter activity of both IFN-β and ISRE when induced by RIG-IN, MAVS, TBK1, IKKε, or cGAS/STING, but not by IRF3/5D (Fig 7A), indicating that MARK3 acts upstream of IRF3, likely at the TBK1/IKKε level, consistent with the role of GEF-H1 on interferon activation [10].

**Fig 7:**
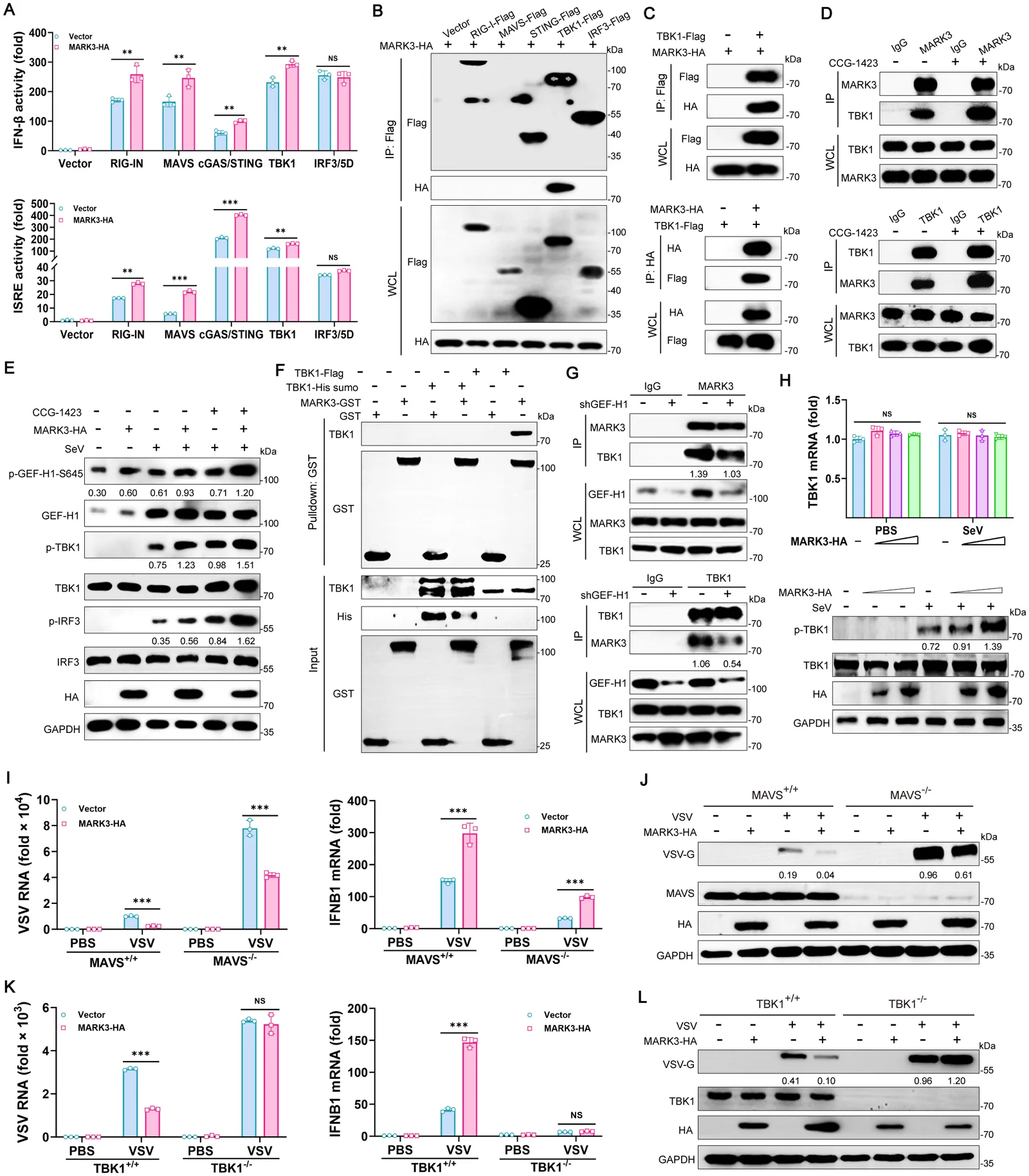
MARK3 mediates antiviral signaling through GEF-H1–mediated TBK1 activation. **(A)** Luciferase reporter assay of the IFNB1 or ISRE promoter in HEK293T cells co-transfected with MARK3-HA and the indicated signaling components (RIG-IN, MAVS, TBK1, IRF3/5D, or cGAS/STING). **(B)** Co-IP of MARK3-HA from HEK293T cells co-expressing Flag-tagged RIG-I, MAVS, STING, TBK1, or IRF3. **(C)** Co-IP of the interaction between TBK1-Flag and MARK3-HA in transfected HEK293T cells. **(D)** Endogenous Co-IP of the MARK3-TBK1 complex in HUVECs treated with or without CCG-1423 (HY-13991, MCE) using anti-MARK3 or anti-TBK1 antibodies. **(E)** The phosphorylation levels of GEF-H1, TBK1 and IRF3 were tested in MARK3-overexpressing HUVECs treated with or without SeV or CCG-1423. **(F)** A GST pulldown assay was performed using MARK3-GST and TBK1, with TBK1 sourced from either bacterial purification (TBK1-His sumo) or overexpressing HEK293T cell lysates (TBK1-Flag). **(G)** The endogenous interaction between MARK3 and TBK1 was assessed in HEK293T cells (top) and HUVECs (bottom) following GEF-H1 knockdown. Immunoprecipitation was performed using the indicated antibodies. **(H)** TBK1 mRNA and protein levels in HUVECs with MARK3-HA dose escalation, with or without SeV stimulation. **(I, J)** VSV and IFN-β mRNA levels (I) and viral protein expression (J) in VSV-infected MAVS^-/-^ HEK293T cells. **(K, L)** VSV and IFN-β mRNA levels (K) and viral protein expression (L) in VSV-infected TBK1^-/-^ HEK293T cells. Data represent mean ± SEM of 3 independent experiments. Statistical analysis was performed using two-way ANOVA followed by Welch’s t-test with Holm correction. *P < 0.05, **P < 0.01, ***P < 0.001; NS, not significant.

To identify MARK3 binding partners in this pathway, we performed Co-IP screening in HEK293T cells expressing Flag-tagged RIG-I, MAVS, STING, TBK1, or IRF3, with or without MARK3-HA. Anti-Flag Co-IP showed that only TBK1 specifically co-precipitated with MARK3. (Fig 7B). This interaction was confirmed in HEK293T cells through reciprocal Co-IP: MARK3 was pulled down with TBK1-Flag using an anti-Flag antibody, and vice versa using an anti-HA antibody (Fig 7C). Given that phosphorylation of GEF-H1 at Ser645 is associated with RhoA activation and actin stabilization [31], and that cytoskeletal reorganization is essential for RLR activation [32], we further validated this endogenous interaction and the phosphorylation of GEF-H1 at Ser645 in HUVECs treated with or without CCG-1423, an inhibitor of the Rho/MRTF/SRF pathway. Our results showed that the inhibitor enhanced the interaction between TBK1 and MARK3 (Fig 7D) as well as the phosphorylation of GEF-H1, TBK1 and IRF3 upon SeV stimulation (Fig 7E), suggesting that the MARK3–GEF-H1–TBK1 axis and the R12C-PP1-RLR axis may act synergistically or complementarily, rather than functioning independently.

Furthermore, in vitro GST pulldown assays confirmed that purified MARK3-GST could bind to TBK1-Flag from lysates of overexpression HEK293T cells (Fig 7F). However, no direct interaction was observed between purified MARK3-GST and purified TBK1-His proteins (Fig 7F), suggesting that their association is indirect and may require additional cellular factors. Strikingly, knockdown of GEF-H1 attenuated the endogenous interaction between MARK3 and TBK1 (Fig 7G), indicating that GEF-H1 serves as the target through which MARK3 potentiates TBK1 activation.

Then we examined whether MARK3 influences TBK1 expression or activation. MARK3 overexpression did not alter TBK1 mRNA or total protein levels but enhanced TBK1 phosphorylation in a dose-dependent manner in HUVECs (Fig 7H). To determine whether TBK1 is required for MARK3-mediated antiviral activity, we infected MAVS-knockout (MAVS^-/-^) and TBK1^-/-^ HEK293T cell lines with VSV. Consistent with our previous findings, overexpression of MARK3 in wild-type HEK293T cells increased IFN-β expression and reduced VSV mRNA and protein levels—effects that were preserved in MAVS^-/-^ cells (Fig 7I and 7J). In contrast, these effects were completely abolished in TBK1^-/-^ cells (Fig 7K and 7L), mirroring the phenotype observed with GEF-H1 (S5G Fig). Collectively, these results demonstrate that MARK3 potentiates antiviral immunity by enhancing interferon activation via a GEF-H1–mediated, TBK1-dependent pathway.

## Discussion

Viral infection triggers sophisticated host defense mechanisms, among which AMP-activated protein kinase (AMPK) plays a well-established role in innate immunity. AMPK directly phosphorylates TBK1 at Ser511 to facilitate the assembly of STING and MAVS signalosomes, thereby enhancing pathogen surveillance [33]. It also restricts Zika virus replication in endothelial cells through suppression of glycolysis [34]. In this study, we identify another AMPK family member, MARK3, as a crucial regulator of antiviral immunity. While MARK3 has been implicated in diverse cellular processes including osteoclast differentiation, cell cycle progression, and ciliated cell differentiation [20], our systematic investigation reveals its essential role in orchestrating interferon-mediated antiviral defense.

We found that MARK3 exerts its antiviral effects by promoting GEF-H1–mediated phosphorylation of TBK1. GEF-H1 is known to be essential for foreign RNA sensing via the RLR signaling pathway, where it promotes IFN-β expression to protect macrophages against RNA viral infection [10]. Here, we confirmed that GEF-H1 enhances both TBK1 and IRF3 phosphorylation, thereby potentiating IFN-I signaling and the expression of ISGs. We further found that the interaction between MARK3 and TBK1 was diminished upon GEF-H1 knockdown. In addition, GST pull-down assays confirmed that this interaction is not direct, indicating that MARK3 modulates TBK1 activation through GEF-H1. This mirrors the cellular phenotypes observed in GEF-H1-deficient models, where MARK3 overexpression failed to enhance TBK1 phosphorylation or restrict viral replication. These findings demonstrate that MARK3 possesses broad-spectrum antiviral activity by activating the GEF-H1–TBK1 axis.

Previous studies have demonstrated that MARK3 phosphorylates GEF-H1 at Ser151 to regulate microtubule dynamics [25]. Although we confirmed this phosphorylation event, the S151A mutant did not abrogate MARK3-mediated interferon activation, suggesting that this residue is dispensable for antiviral signaling. Instead, through comprehensive mutational analysis, we identified Ser645 as the key functional phosphorylation site. Nevertheless, the precise mechanism by which Ser645 phosphorylation facilitates antiviral immunity warrants further investigation. The S645A mutation completely abolished MARK3-enhanced GEF-H1 phosphorylation and subsequent TBK1 activation, whereas phosphomimetic mutants (S645D/E) fully restored interferon signaling. Importantly, kinase-active MARK3, but not the T211A kinase-dead mutant, specifically phosphorylated GEF-H1 at Ser645 in response to viral infection, underscoring the essential role of MARK3 catalytic activity in this signaling pathway.

Although this study systematically elucidates the role of the MARK3–GEF‑H1–TBK1 axis in antiviral immunity, several key questions remain unresolved. First, while MARK3 kinase activity has been implicated in antiviral responses, it is still unclear whether viral infection directly triggers MARK3 activation, and if so, whether this involves upstream regulation by LKB1 [35] or virus‑induced metabolic alterations. Additionally, structural analyses—such as cryo‑electron microscopy or X‑ray crystallography—will be essential to clarify, at the molecular level, how site‑specific phosphorylation modulates the interaction between GEF‑H1 and TBK1. Given that GEF‑H1 is known to participate in NLRP3 inflammasome activation [36], it may exert broader immunomodulatory functions beyond antiviral defense. Thus, an open question remains whether the MARK3–GEF‑H1 axis also contributes to antibacterial or antifungal immunity, either independently or in parallel with its established antiviral role.

The integrity of the cytoskeleton is required for the MARK3–GEF-H1–TBK1 axis. Release of GEF-H1 from microtubules activates RhoA[15], yet nocodazole dampened the TBK1–GEF-H1 interaction and TBK1/IRF3 phosphorylation, suggesting that the microtubule-associated pool of GEF-H1, rather than the soluble RhoA-activating pool, scaffolds signalling [10]. Accordingly, MARK3–TBK1 complexes persisted after inhibition of RhoA/MRTF/SRF transcription by CCG-1423 [37,38]. These interactions and their downstream effects are therefore most likely attributable to the preservation of the cytoskeletal architecture that permits signalosome assembly [32], rather than to a direct effect on TBK1 activation, with TBK1 Ser172 phosphorylation being a consequence rather than a trigger. We cannot, however, exclude a requirement for these interactions in RhoA-mediated MAVS or STING rearrangement [39,40]. Notably, MARK3 remained functional in MAVS^-/-^ cells but was completely inactive in TBK1^-/-^ cells, indicating that a MAVS-dependent rearrangement step is dispensable for the RNA-sensing arm, while a contribution to STING rearrangement in DNA sensing remains to be tested [40].

In conclusion, our study establishes MARK3 as a key regulator of broad-spectrum antiviral immunity through its role in phosphorylating GEF-H1 at Ser645. This post-translational modification enables GEF-H1 to bridge MARK3 to TBK1, leading to enhanced interferon production and establishment of an antiviral state. The MARK3–GEF-H1–TBK1 axis thus represents a previously unrecognized signaling pathway that complements known AMPK-mediated antiviral mechanisms, expanding our understanding of how kinase networks orchestrate innate immune defenses against viral infection.

## Materials and methods

### Ethics statement

MARK3^-/-^ mice on a C57BL/6 genetic background were generated by Cyagen Biosciences (Guangzhou, China) via targeted disruption of the MARK3 locus (NCBI RefSeq: NM_001370744.1). The care and use of experimental animals in this study were approved by the Ethics Committee of Anhui Medical University (Hefei, China). All procedures complied with the Laboratory Animal Management Regulations and were conducted under the oversight of the University’s Scientific Investigation Board.

### Cells

HUVEC, HEK293T, and Vero cells were cultured in Dulbecco’s Modified Eagle’s Medium (DMEM) supplemented with 10% fetal bovine serum (FBS), penicillin (100 U/mL), and streptomycin (100 μg/mL). PMs and BMDMs were isolated from 6- to 8-week-old mice four days after intraperitoneal injection with 2.5 mL of thioglycolate broth (BD) and were cultured in RPMI-1640 medium supplemented with 10% FBS. All cells were incubated at 37°C in a humidified atmosphere containing 5% CO_2_.

### Plasmids

The human MARK3 and GEF-H1 genes were amplified by PCR from cDNA reverse-transcribed from RNA extracted from HEK293T cells and subsequently cloned into the pCMV-HA and pFlag-CMV-2 vectors. All IFN-β and ISRE promoter luciferase reporter plasmids, as well as expression constructs for RIG-I, MAVS, cGAS, STING, TBK1, and IRF3, were obtained from our laboratory stock. For the construction of shRNAs, double-stranded oligonucleotides corresponding to the target sequences were cloned into the pGreenPuro shRNA expression lentivector plasmid. All constructs were verified by DNA sequencing. The primer sequences used are listed in S2 Table.

### Antibodies

The following primary antibodies were used for immunoprecipitation (IP), immunoblotting (IB) and flow cytometry (FCM): anti-MARK3 (Affinity, DF3135), anti-GEF-H1 (Proteintech, D52C2), anti-TBK1/NAK (ABclonal, A3458), anti-phospho-TBK1/NAK (Ser172) (Proteintech, 82383–1-RR), anti-IRF3 (ABclonal, A2172), anti-phospho-IRF3 (Ser396) (ABclonal, AP1412), anti-FLAG (Sangon Biotech, D110005), anti-HA (Sangon Biotech, D110004), and anti-VSV-G (Abcam, AB183497), Brilliant Violet 421 anti-mouse CD3e (BioLegend, 100341), Pacific Blue anti-mouse/human CD45R/B220 Antibody (BioLegend, 103227), PE/Cyanine7 anti-mouse CD11c Antibody (BioLegend, 117318) and PerCP/Cyanine5.5 anti-mouse/human CD11b Antibody (BioLegend, 101228), HRP-conjugated secondary antibodies against mouse (Sangon Biotech, D110087) or rabbit (Sangon Biotech, D110058) were also used. The polyclonal antibody specific to GEF-H1 phosphorylated at Ser645 was generously provided by Shan Gao (Southeast University, Nanjing) [31].

### Luciferase reporter assay

HEK293T cells were plated in 24-well plates and transiently transfected with 50 ng of a luciferase reporter plasmid along with an equal total amount of various expression plasmids or corresponding empty vector controls. To normalize transfection efficiency, 10 ng of pRL-TK plasmid was co-transfected as an internal control. After 36 hours, the cells were stimulated with Sendai virus (SeV) for 12 hours. Transcriptional activity was quantitatively measured using the Dual-Luciferase Reporter Assay Kit (YEASEN, 11402ES60) according to the manufacturer’s instructions. Briefly, transfected cells were harvested and lysed with 1 × passive lysis buffer for 15 minutes at room temperature. Firefly luciferase activity was measured first by adding Luciferase Assay Reagent II (LAR II) to the lysate, followed by immediate luminescence detection. Renilla luciferase activity was then assessed after adding Stop & Glo Reagent to quench the firefly signal. Relative luciferase activity was calculated by normalizing firefly luminescence values to the corresponding Renilla luciferase values.

### Mass spectrometry

To identify potential MARK3 interacting proteins or GEF-H1 phosphorylation sites, HUVECs expressing MARK3-HA with VSV infection or GEF-H1-Flag with SeV stimulation were lysed using Western lysis buffer. Immunoprecipitation was performed with anti-HA or anti-Flag magnetic beads (Bimake, A02046). The immunoprecipitated proteins were separated by SDS-PAGE and briefly stained with Coomassie Blue. The gel band corresponding to MARK3 was excised, digested with trypsin, and analyzed by LC-MS/MS on an Orbitrap Elite mass spectrometer (Thermo Fisher) coupled to a Dionex nanoLC system. MS/MS spectra were acquired with an isolation window of 0.02 Da and processed using Proteome Discoverer 1.4 against the UniProt database.

### Quantitative RT-PCR

Total RNA was extracted from cells and homogenized tissues using TRIzol reagent (SparkJade, AC0101-B). Then, 0.5–1 µg of total RNA was reverse-transcribed into cDNA using PrimeScript RT Master Mix (Takara, DRR036A). Quantitative PCR was performed with ChamQ Universal SYBR qPCR Master Mix (Vazyme, Q311). Gene expression levels were normalized to GAPDH, and the relative quantification was calculated using the 2^–ΔΔCt^ method. The primer sequences used are provided in S2 Table.

### Immunoprecipitation and immunoblot analysis

Cells were lysed with Cell Lysis Buffer for Western and incubated on ice for 10 minutes, followed by centrifugation at 12,000 × g for 15 minutes at 4°C. The supernatants were collected and boiled at 100°C for 5 minutes. Denatured proteins were separated by SDS-PAGE and transferred to PVDF membranes. The membranes were blocked with 5% non-fat milk in TBST for 1 hour at room temperature, then incubated with primary and secondary antibodies accordingly. Protein signals were detected using enhanced chemiluminescence (ECL) substrate.

For exogenous protein interactions, cells transfected with the indicated plasmids were lysed in ice-cold lysis buffer (50 mM Tris-HCl [pH 7.5], 250 mM NaCl, 0.5% NP-40, 1 mM EDTA) supplemented with 0.1% protease inhibitor cocktail. Lysates were incubated with anti-HA (Bimake, A02046) or anti-Flag (Bimake, B26101) conjugated agarose beads for 4 h at 4°C with gentle rotation. After washing three times with lysis buffer, the immunoprecipitates were boiled and subjected to immunoblot analysis.

For endogenous interactions, primary antibodies were pre-incubated with protein A/G agarose beads for 30 minutes at room temperature. The antibody-bead complexes were then incubated with cell lysates for 3 hours at room temperature with rotation. Beads were washed 3–4 times with lysis buffer, and bound proteins were analyzed by immunoblotting.

### Viral infection

VSV, HSV-1, CVB3, and RSV were propagated in Vero cells. Viral supernatants were collected and stored as stock solutions, and titers were determined by TCID₅₀ assay in Vero cells. SeV was amplified in 10-day-old specific pathogen-free (SPF) embryonated chicken eggs. The allantoic fluid was harvested and titrated by TCID₅₀ assay in Vero cells.

For infection experiments, cells were seeded at a density of 2 × 10^5^ cells per well and cultured for 24 hours before infection. The multiplicities of infection (MOI) used in all experiments were as follows: 0.1 for VSV, 1 for HSV-1, 0.1 for CVB3, 0.1 for RSV, and 0.1 for SeV.

### Plaque assay

Virus-containing medium was collected and serially diluted, then used to infect confluent Vero cells cultured in 24-well plates. After 1 hour of incubation, the inoculum was removed, and the cells were washed twice with PBS. A culture medium overlay containing 2% methylcellulose was applied and maintained for 24 hours (for VSV) or 48 hours (for HSV-1). The overlay was then aspirated, and cells were fixed with 4% paraformaldehyde for 15 minutes, followed by staining with 1% crystal violet in 70% ethanol for 30 minutes. After two washes with ddH_2_O, plaques were counted. The average plaque count was multiplied by the dilution factor to calculate the viral titer in plaque-forming units per milliliter (PFU/mL).

### Viral infection *in vivo*

Age- and sex-matched C57BL/6J littermates were used for all *in vivo* experiments. Eight-week-old male and female mice were intraperitoneally injected with VSV (5 × 10^8^ PFU). At 24 hours post-infection, blood was collected from the orbital sinus for ELISA and viral titer analysis. Heart, kidney, lung, spleen, and liver tissues were harvested for quantitative PCR to assess viral load and expression of ISGs. In survival studies, mice infected with VSV were monitored daily for mortality.

### CRISPR-Cas9–mediated genome editing

Small guide RNAs (sgRNAs) were cloned into the lentiCRISPRv2 vector (Addgene) and transfected into HEK293T cells. 48 h after transfection, the cells were placed under puromycin selection for one week. Single cells were then isolated by serial dilution and expanded to establish clonal cell lines [41]. Successful knockout was confirmed by immunoblot analysis. The sgRNA sequences used are listed in S2 Table.

### Flow cytometry

Mice were euthanized, and lymph node tissues were aseptically excised. The harvested tissues were placed in phosphate-buffered saline (PBS, pH 7.4) containing 2% FBS. Single-cell suspensions were generated by gently mincing the tissues through a 70 μM cell strainer. The cells were stained with a cocktail of fluorophore-conjugated antibodies at predetermined optimal titrations to delineate distinct cellular lineages, including Brilliant Violet 421 anti-mouse CD3e, Pacific Blue anti-mouse/human CD45R/B220, PE/Cyanine7 anti-mouse CD11c, and PerCP/Cyanine5.5 anti-mouse/human CD11b, and finally resuspended in 200 μL of FACS buffer for cytometric acquisition.

### Indirect immunofluorescence

HeLa cells were seeded in 12-well plates containing 18 mm cell coverslips and subjected to transfection and viral infection treatments, respectively. The cells were then fixed with 4% paraformaldehyde for 18 min and permeabilized with 0.3% Triton X-100 for 10 min. After washing with PBS, the cells were blocked with 5% bovine serum albumin (BSA) for 1 h. Subsequently, primary antibodies against HA (Proteintech, 66006–2-Ig), Flag (Proteintech, 66008–4-Ig), Myc (Vazyme, RA1005), and β-tubulin (Servicebio, GB152667) diluted in blocking solution were added, followed by incubation in a humid chamber at 4°C overnight. After washing the cells three times with PBST, fluorescent secondary antibodies—Goat Anti-Rabbit IgG H&L (iFlourTM 488, HA1121) and Goat Anti-Mouse IgG H&L (iFlourTM 647, HA1127)—were added and incubated in the dark for 1 h. The coverslips were then mounted using an anti-fluorescence quenching mounting medium containing DAPI (4’,6-diamidino-2-phenylindole; Biosharp, BL739A). Images were acquired using a laser confocal microscope.

### Statistical analysis

Statistical analysis was performed using two-way ANOVA followed by Welch’s t-test with Holm correction, unless otherwise noted in the figure legends. Significance is denoted in the figures as follows: *P < 0.05, **P < 0.01, and ***P < 0.001; differences with a P value of 0.05 or less were considered statistically significant. For mouse survival studies, Kaplan–Meier curves were generated and analyzed for statistical significance using the log-rank test in GraphPad Prism version 8.0. Pilot studies were conducted to estimate sample sizes required to achieve sufficient statistical power.

## Supporting information

S1 Fig Association between MARK3 and H1N1/VSV infection. (**A**) A correlation scatter plot was generated by analyzing the Module Membership against Gene Significance (MM-GS). (**B**) Hub genes concurrently involved in both viral infection processes were identified using a dual-dimensional screening strategy.
(TIF)

S2 Fig MARK3 suppresses viral replication in HEK293T cells. (**A**) VSV-G replication, indicated by GFP fluorescence, was monitored via fluorescence microscopy (scale bar: 50 μm) and quantified by flow cytometry. (**B, C**) VSV (B) and HSV-1 (C) mRNA levels, protein expression, and progeny titers were measured in MARK3-overexpressing cells. (**D, E**) Viral genome copies, protein levels, and progeny production of VSV (D) and HSV-1 (E) were assessed in MARK3-knockdown cells. Data are shown as mean ± SEM from three independent experiments. Statistical significance was determined using two-way ANOVA followed by Welch’s t-test with Holm correction. *P < 0.05, **P < 0.01, ***P < 0.001; NS, not significant.
(TIF)

S3 Fig MARK3 enhances the expression of IFN-β and ISGs in HEK293T cells. (**A, B**) mRNA levels of IFN-β (A), along with IFIT1, ISG15 and CIG5 (B) from MARK3-overexpressing cells following stimulation with SeV or Poly(I:C). (**C, D**) mRNA levels of IFN-β (C) and the indicated ISGs (D) in MARK3-silenced cells after SeV or Poly (I:C) stimulation. Data represent mean ± SEM of 3 independent experiments. Statistical analysis was performed using two-way ANOVA followed by Welch’s t-test with Holm correction. *P < 0.05, **P < 0.01, ***P < 0.001; NS, not significant.
(TIF)

S4 Fig The effects of MARK3 on the development of various immune cells. The proportions of B cells, T cells, myeloid cells, and dendritic cells (DCs) in the spleen (**A**) and lymph nodes (**B**) were analyzed via flow cytometry using antibodies against CD45R, CD3e, CD11b, and CD11c, respectively. Data represent mean ± SEM of 3 independent experiments. Statistical analysis was performed using two-way ANOVA followed by Welch’s t-test with Holm correction. *P < 0.05, **P < 0.01, ***P < 0.001; NS, not significant.
(TIF)

S5 Fig GEF-H1 exhibits antiviral immune activity via TBK1 activation. (**A, B**) mRNA and protein levels of GEF-H1 in HUVECs stimulated with SeV (A) or Poly(I:C) (B) at the indicated time points. (**C, D**) Viral protein levels of VSV and HSV-1 in HUVECs following GEF-H1 overexpression (C) or knockdown (D). (**E**) Co-IP analysis of TBK1 and GEF-H1 in mouse PMs with or without nocodazole (A260772, Ambeed) treatment. (**F**) Phosphorylation of TBK1 and IRF3 in SeV-stimulated mouse PMs in the presence of nocodazole. (**G**) The mRNA levels of VSV and IFN-β from VSV-infected TBK1^-/-^ HEK293T cells with or without GEF-H1 ectopic expression. Data represent mean ± SEM of 3 independent experiments. Statistical analysis was performed using two-way ANOVA followed by Welch’s t-test with Holm correction. *P < 0.05, **P < 0.01, ***P < 0.001; NS, not significant.
(TIF)

S6 Fig Ser645 is the potential phosphorylation site in GEF-H1 to enhance antiviral activity. (**A**) IFN-β mRNA levels in SeV-stimulated HUVECs transfected with GEF-H1 WT or the indicated mutants. (**B**) VSV mRNA and protein levels in HUVECs expressing GEF-H1-Flag WT or mutant constructs. (**C**) Co-IP assays between TBK1-HA and wild-type GEF-H1-Myc or its S645A mutant. (**D**) IFN-β promoter activity in HEK293T cells overexpressing GEF-H1 S645 mutants upon RIG-IN or SeV stimulation. (**E**) TBK1 and IRF3 phosphorylation levels in SeV-stimulated HUVECs expressing S645 mutants. (**F**) IFN-β and ISG mRNA levels in S645 mutant-transfected HUVECs with SeV stimulation. (**G, H**) Viral mRNA and protein levels of VSV (G) or HSV-1 (H) in HUVECs expressing GEF-H1-Flag WT or Ser645 mutants. Data represent mean ± SEM of 3 independent experiments. Statistical analysis was performed using two-way ANOVA followed by Welch’s t-test with Holm correction. *P < 0.05, **P < 0.01, ***P < 0.001; NS, not significant.
(TIF)

S1 Table Part of Iped Proteins by GEF-H1 with SeV stimulation. (XLSX)

S2 Table Sequences of Oligo-primers or peptides used in this study. (XLSX)

S1 Raw Images All original, unprocessed blot images are included in this supplementary figure. (PDF)

S2 Raw qPCR Data All original qRT-PCR amplification data are included in this supplementary file. (XLSX)
