# Supplementary material for: MARK3 promotes host antiviral defense by driving GEF-H1–mediated TBK1 activation
Source: PLoS Pathog. 2026 Oct 27;22(10):e1014701. doi: 10.1371/journal.ppat.1014701 (PMC13712703; doi:10.1371/journal.ppat.1014701)

Figures

Fig. 1A

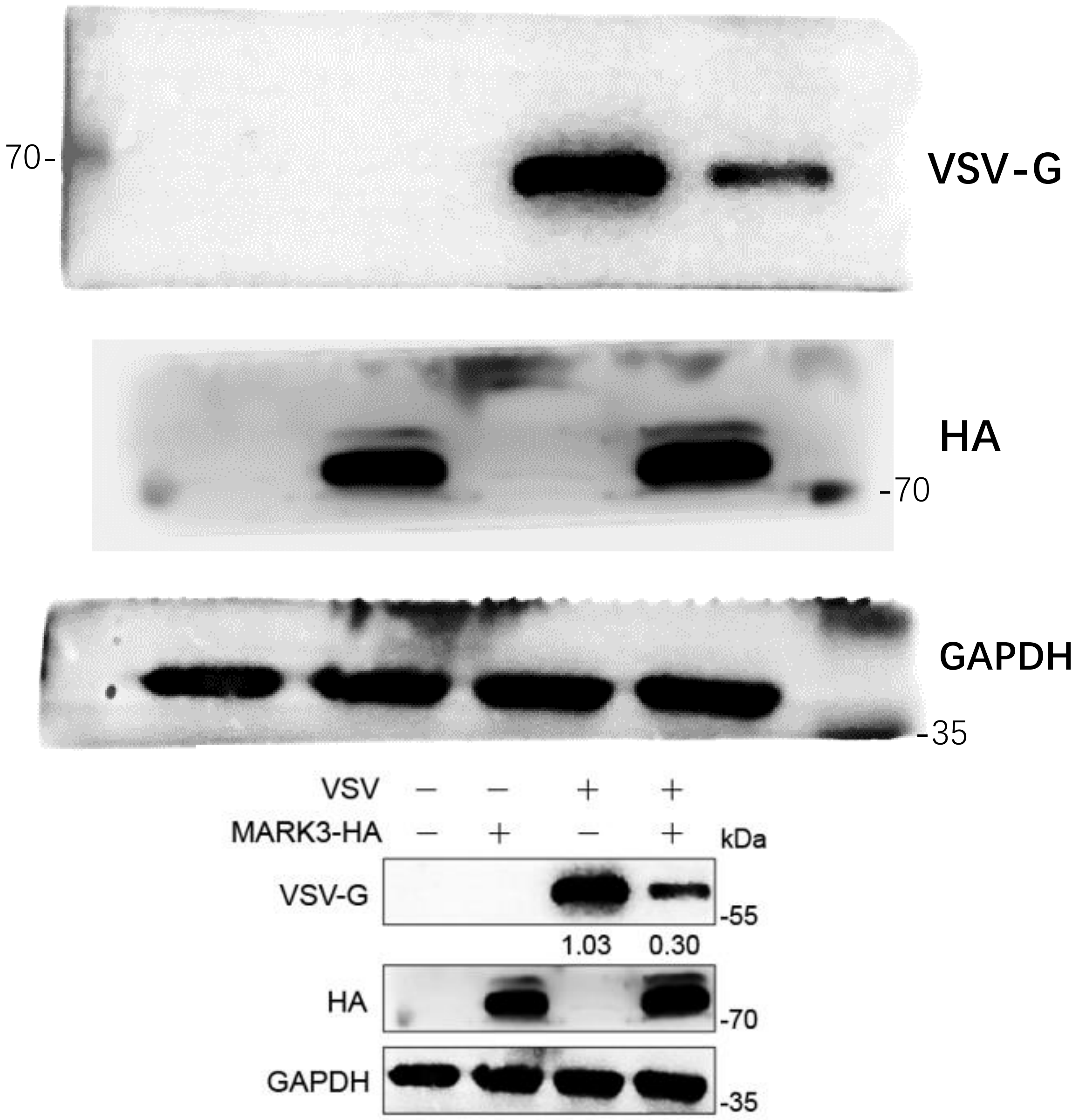

Fig. 1B

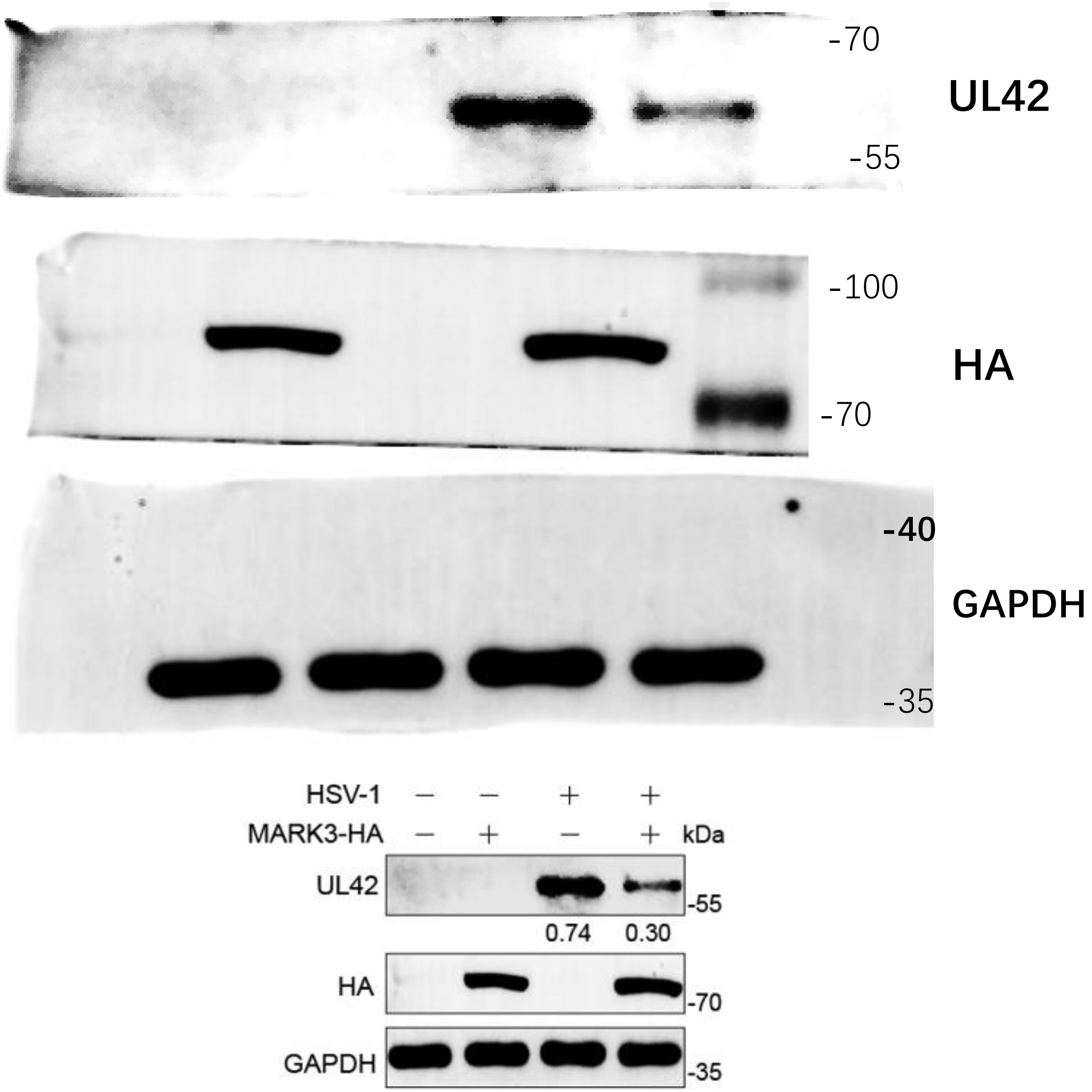

Fig. 1C

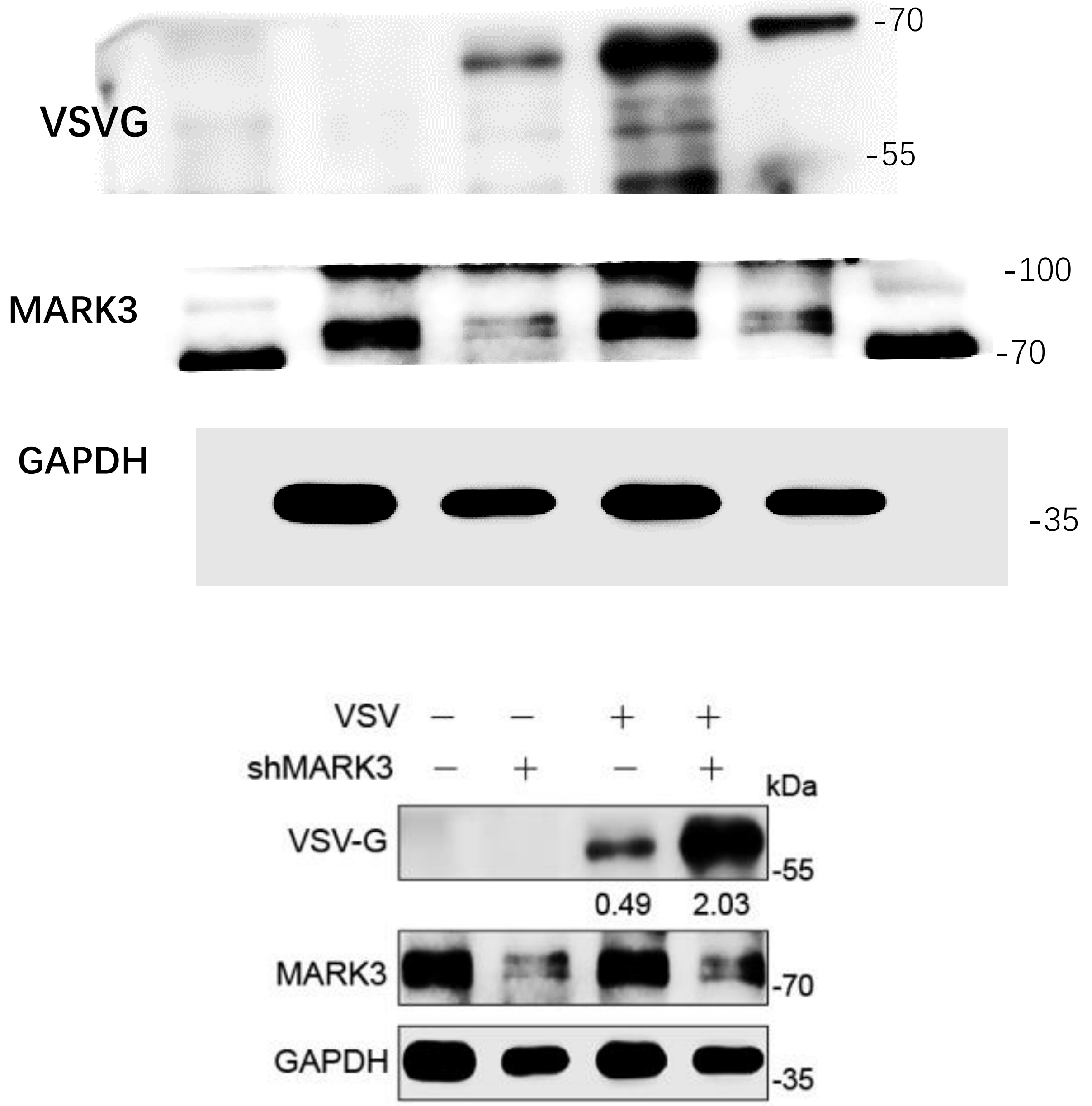
VSVG
-70
-55
MARK3
-100
-70
GAPDH
-35
VSV − − + +
shMARK3 − + − +
kDa
VSV-G
-55
0.49 2.03
MARK3
-70
GAPDH
-35

Fig. 1D

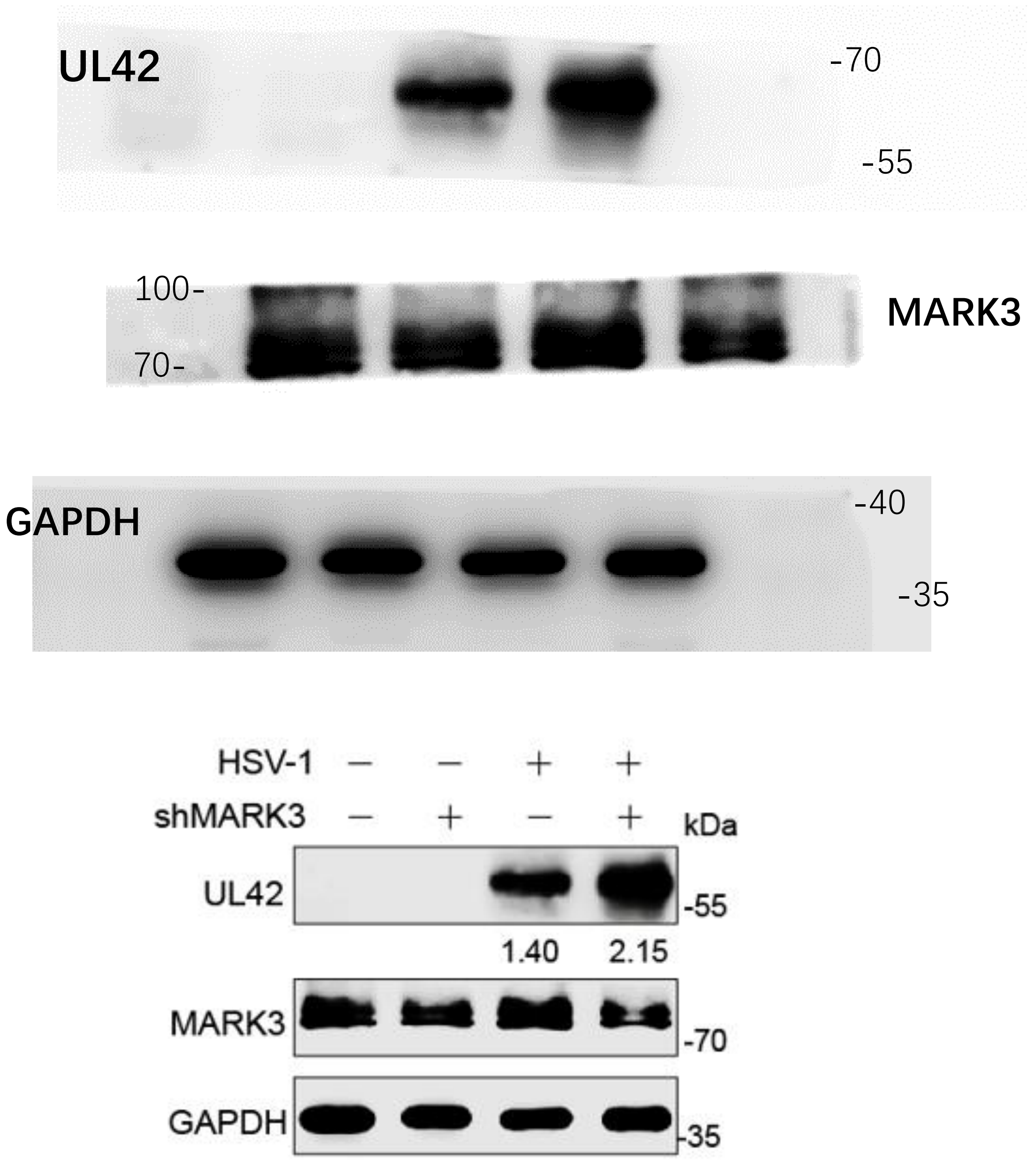
UL42
-70
-55
100-
70-
MARK3
GAPDH
-40
-35
HSV-1 − − + +
shMARK3 − + − +
kDa
UL42
-55
1.40 2.15
MARK3
-70
GAPDH
-35

Fig. 1G

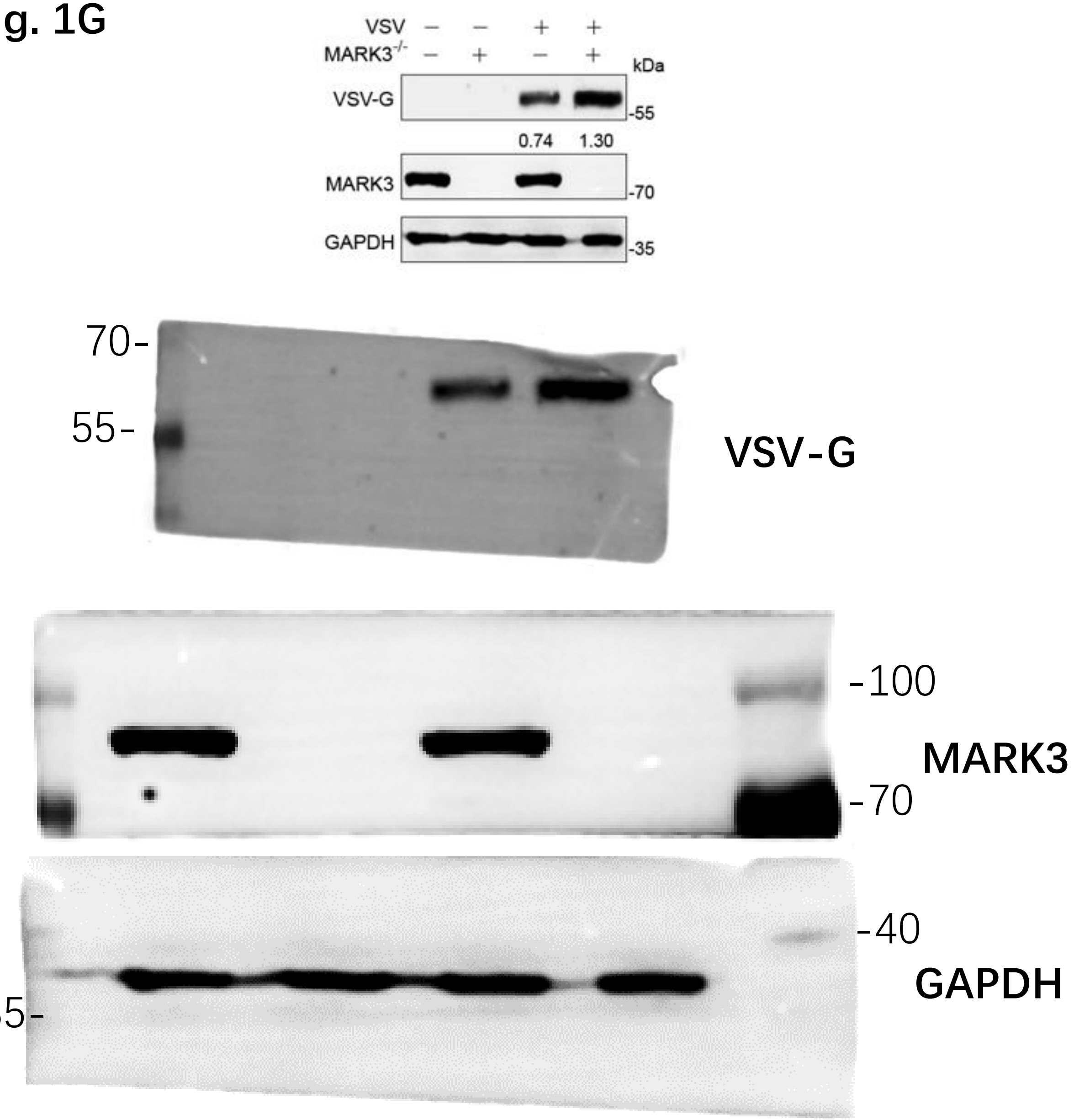
VSV − − + +
MARK3-/- − + − +
kDa
VSV-G
-55
0.74 1.30
MARK3
-70
GAPDH
-35
70-
55-
VSV-G
-100
MARK3
-70
-40
GAPDH
35-

Fig. 1H

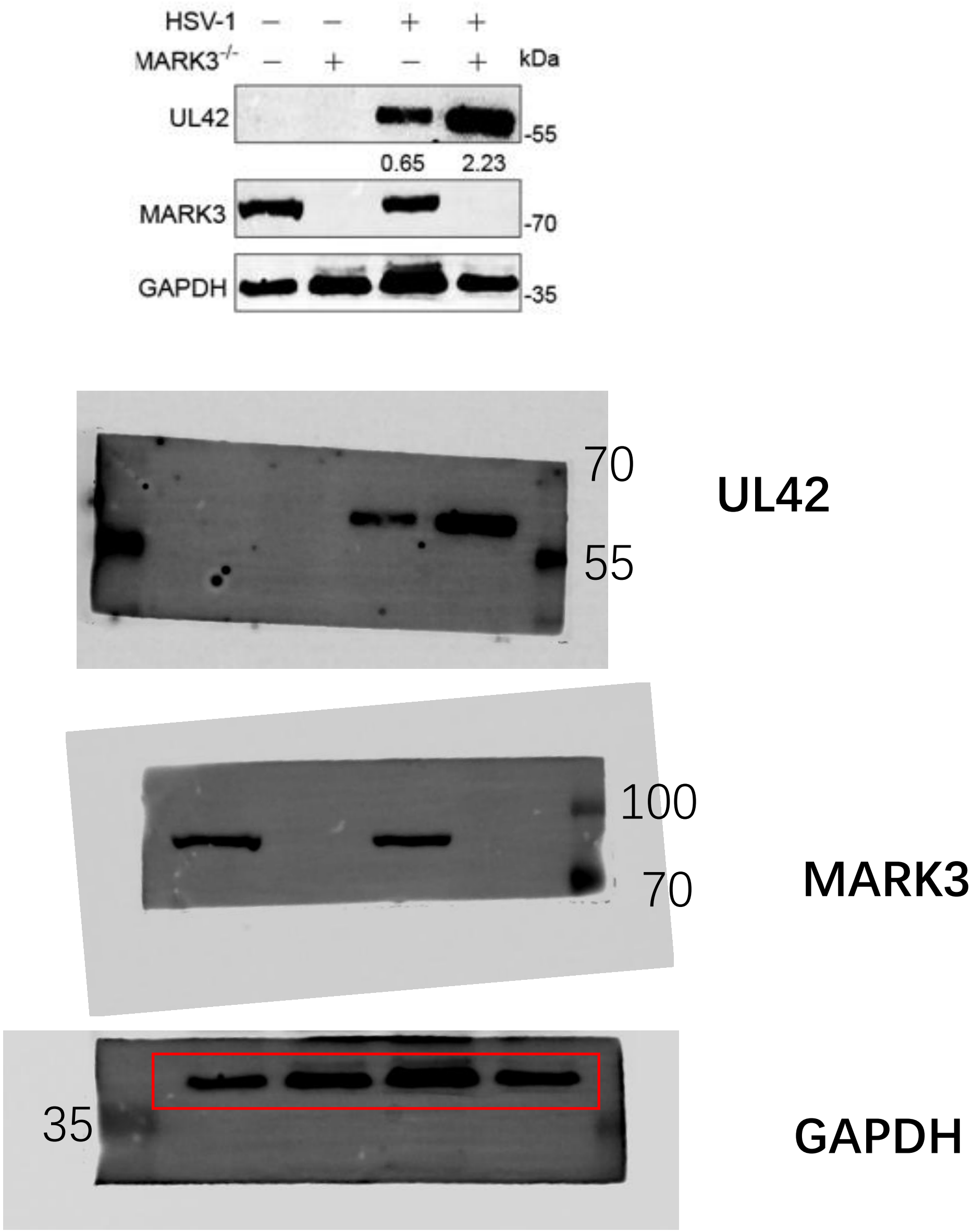
HSV-1 − − + +
MARK3-/- − + − +
kDa
UL42
-55
0.65 2.23
MARK3
-70
GAPDH
-35
70
55
UL42
100
70
MARK3
35
GAPDH

Fig. 2D

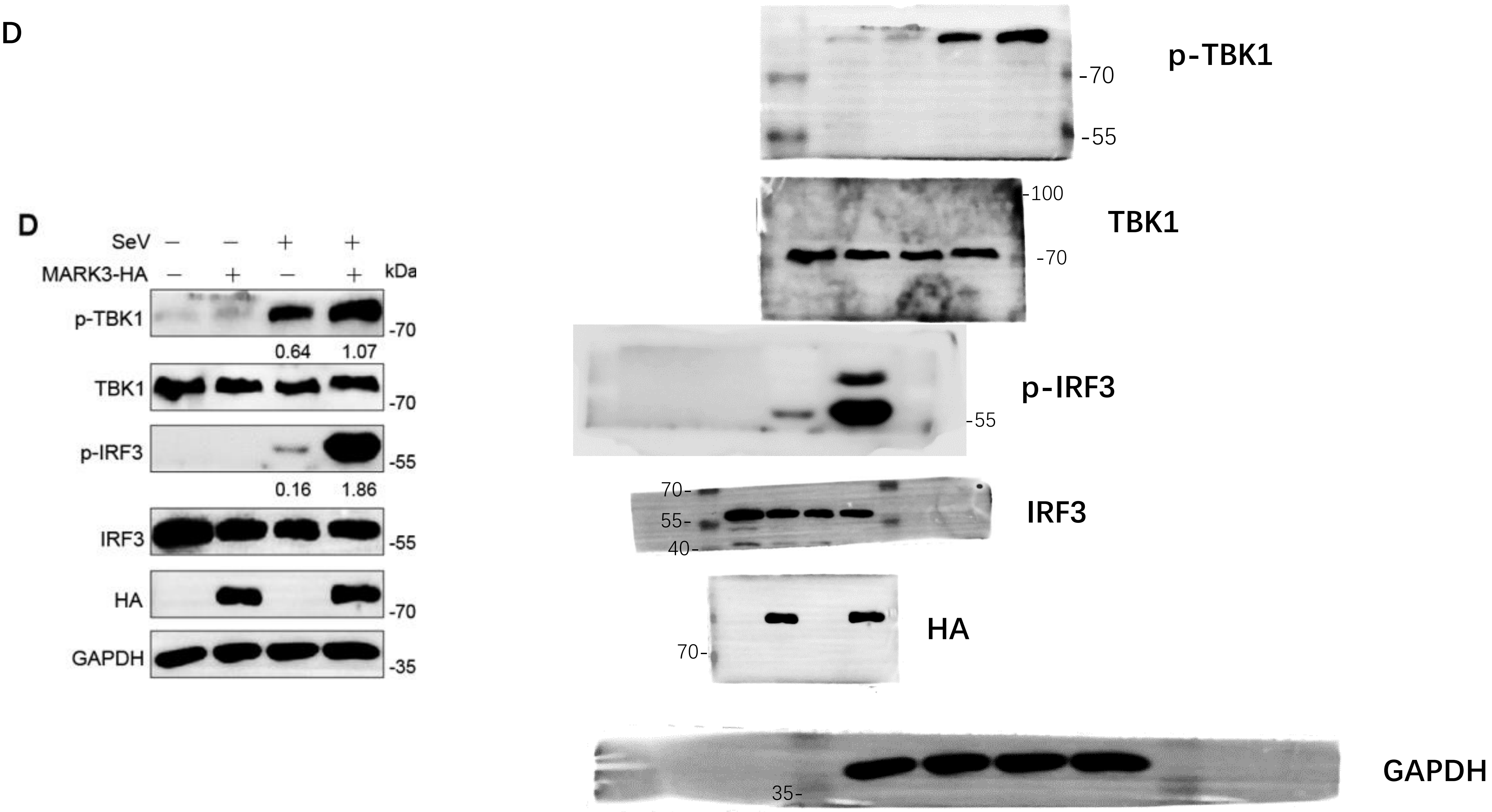
D
SeV − − + +
MARK3-HA − + − +
kDa
p-TBK1
-70
0.64 1.07
TBK1
-70
p-IRF3
-55
0.16 1.86
IRF3
-55
HA
-70
GAPDH
-35
p-TBK1
-70
-55
TBK1
-100
-70
p-IRF3
-55
IRF3
70-
55-
40-
HA
70-
GAPDH
35-

Fig. 2H

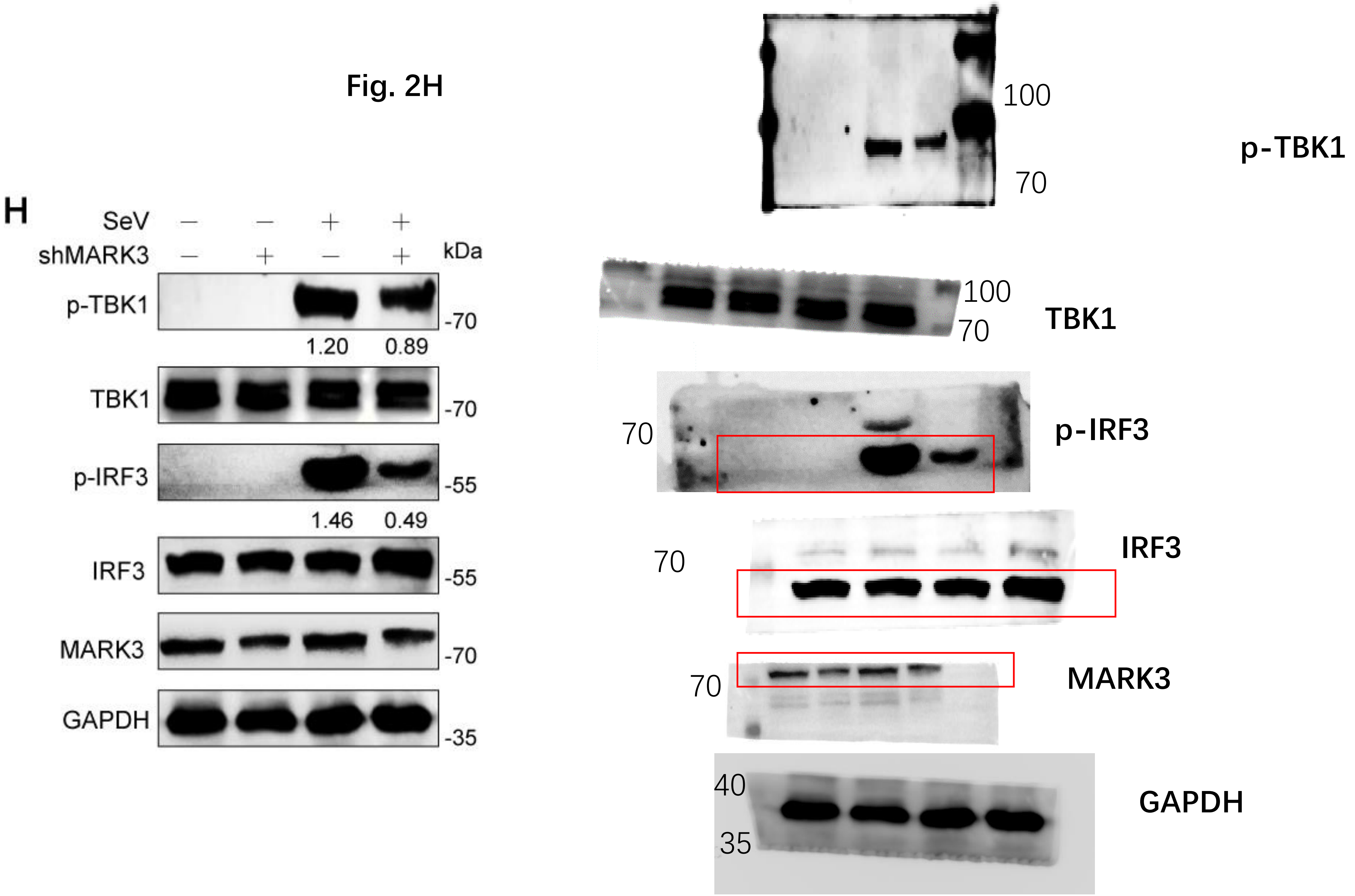
H
SeV
shMARK3
kDa
p-TBK1
-70
1.20
0.89
TBK1
-70
p-IRF3
-55
1.46
0.49
IRF3
-55
MARK3
-70
GAPDH
-35
100
70
p-TBK1
100
70
TBK1
70
p-IRF3
70
IRF3
70
MARK3
40
35
GAPDH

Fig. 2J

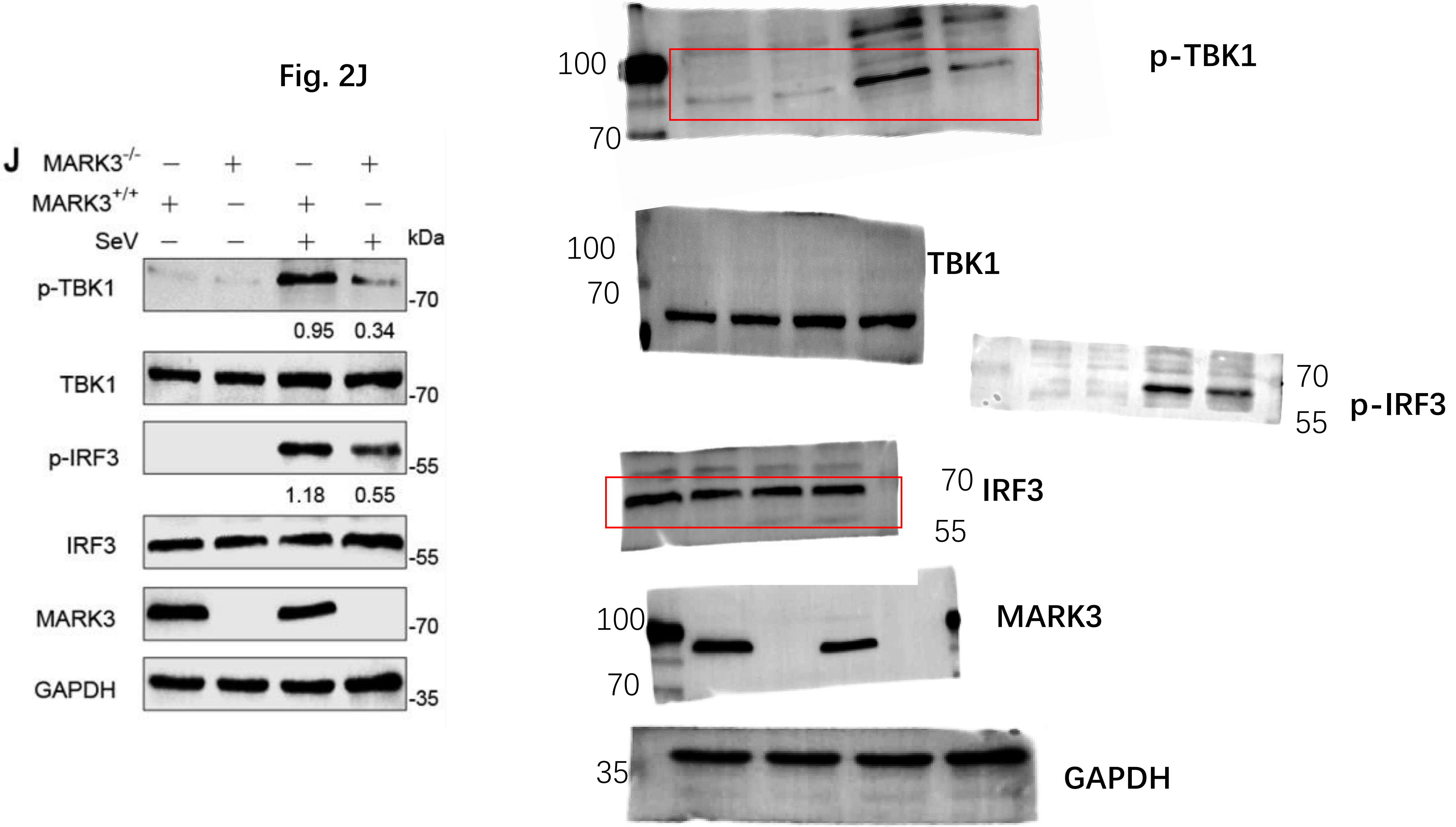

J
MARK3-/-
MARK3+/+
SeV
kDa
p-TBK1
TBK1
p-IRF3
IRF3
MARK3
GAPDH
0.95 0.34
1.18 0.55
-70
-55
-35
100
70
55
35

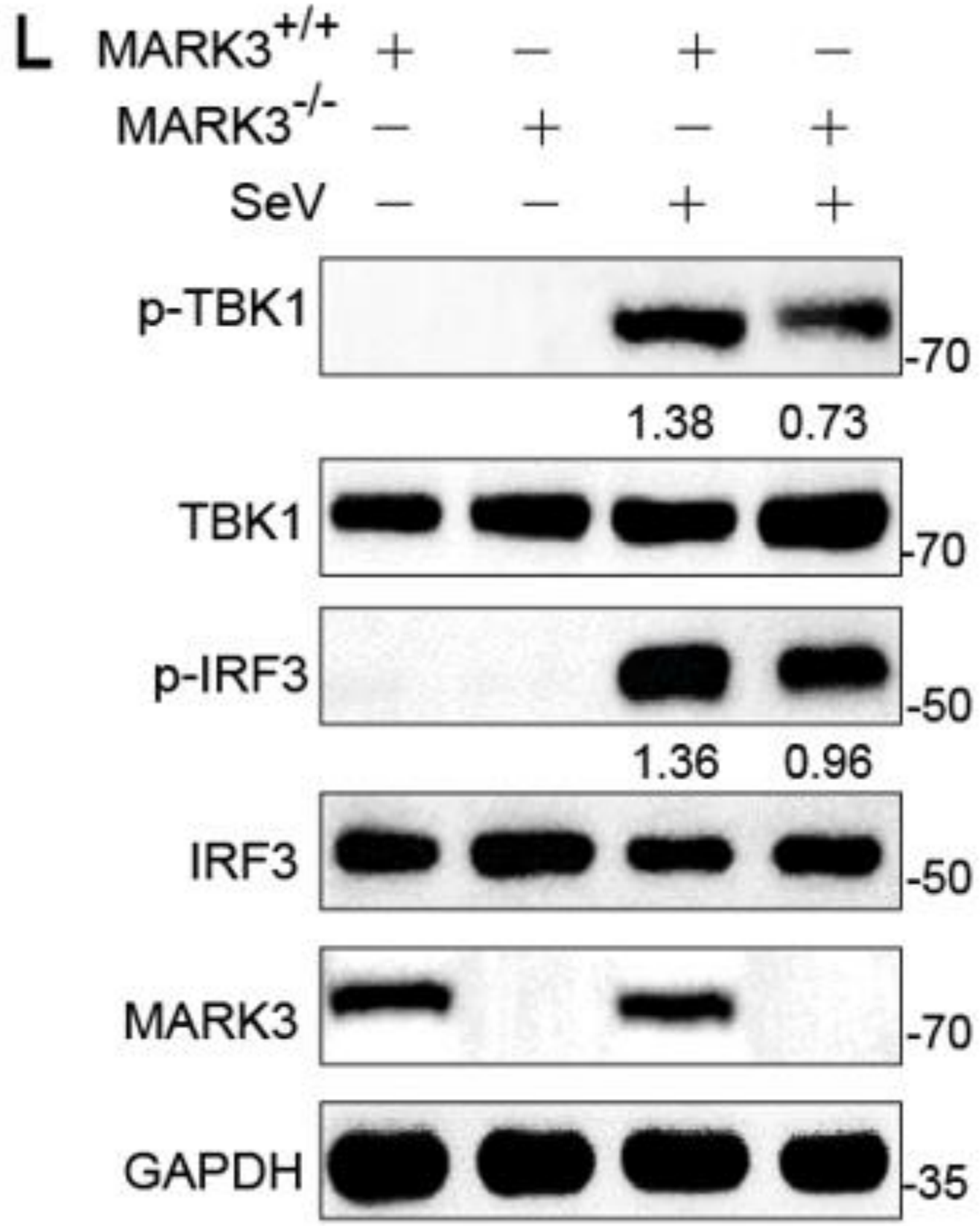
L
MARK3+/+ + − + −
MARK3-/- − + − +
SeV − − + +
p-TBK1
-70
1.38 0.73
TBK1
-70
p-IRF3
-50
1.36 0.96
IRF3
-50
MARK3
-70
GAPDH
-35

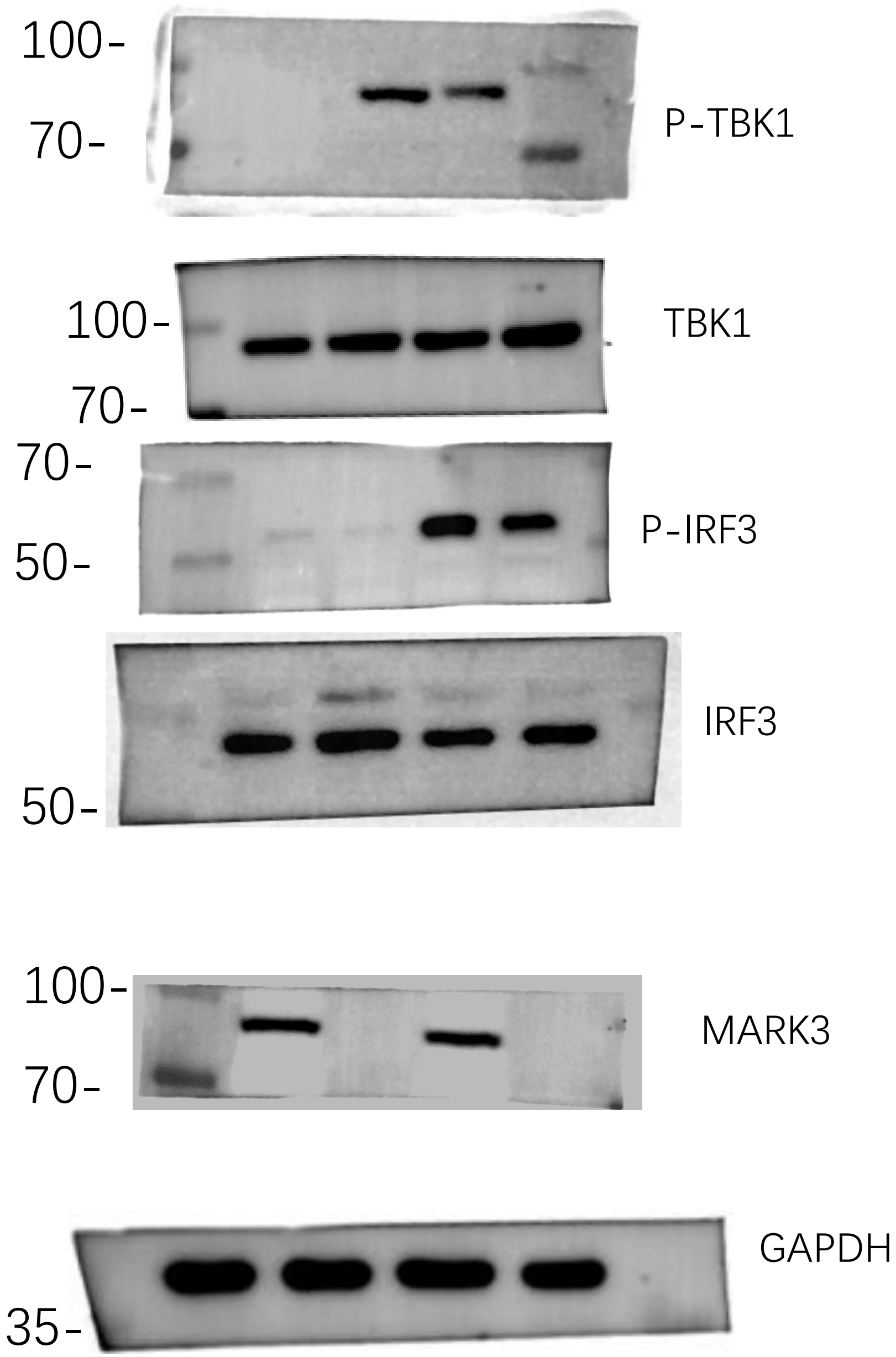
100-
70-
P-TBK1
100-
70-
TBK1
70-
50-
P-IRF3
IRF3
50-
100-
70-
MARK3
35-
GAPDH

Fig. 3E

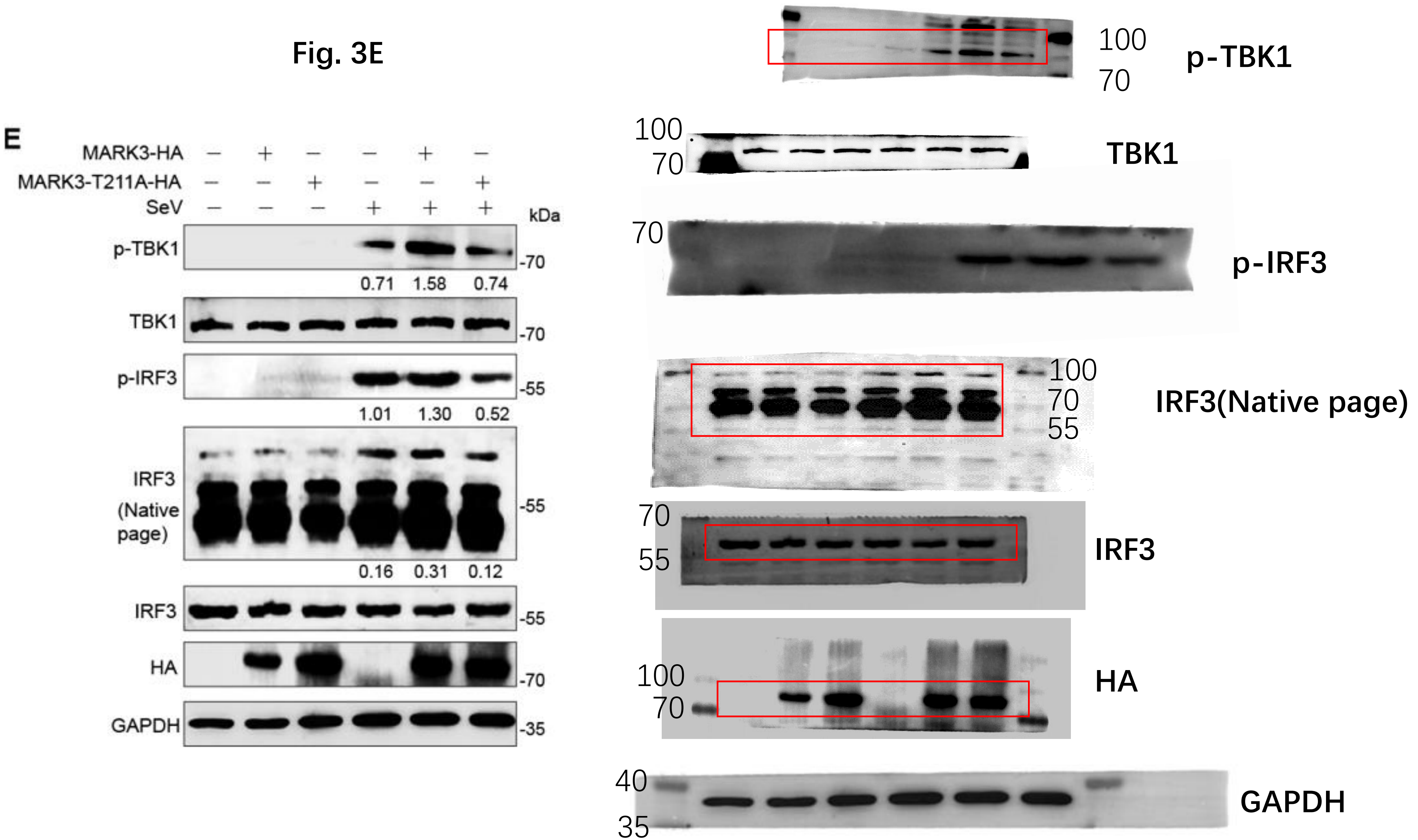
E
MARK3-HA – + – – + –
MARK3-T211A-HA – – + – – +
SeV – – – + + +
kDa
p-TBK1
-70
0.71 1.58 0.74
TBK1
-70
p-IRF3
-55
1.01 1.30 0.52
IRF3 (Native page)
-55
0.16 0.31 0.12
IRF3
-55
HA
-70
GAPDH
-35
100
70
p-TBK1
100
70
TBK1
70
p-IRF3
100
70
55
IRF3(Native page)
70
55
IRF3
100
70
HA
40
35
GAPDH

Fig. 3F

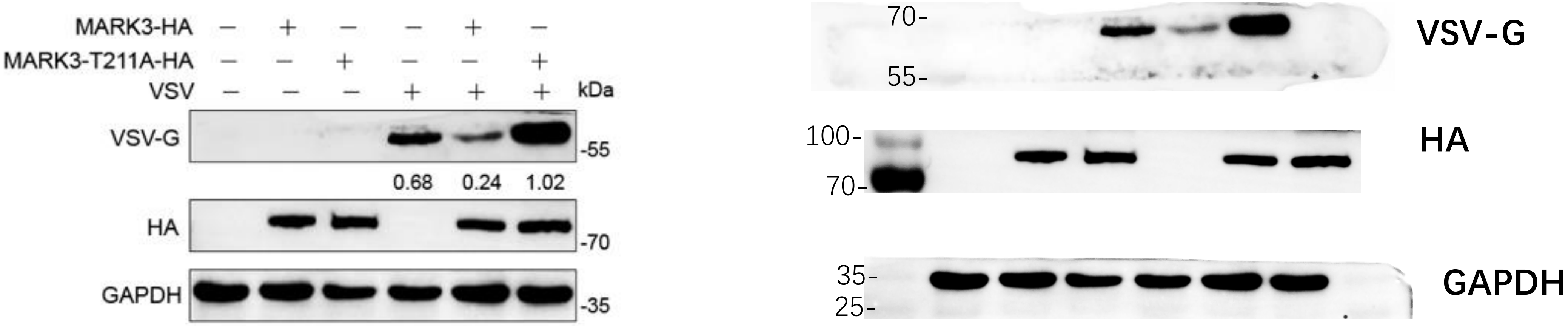
MARK3-HA − + − − + −
MARK3-T211A-HA − − + − − +
VSV − − − + + +
kDa
VSV-G
-55
0.68 0.24 1.02
HA
-70
GAPDH
-35
70-
55-
VSV-G
100-
70-
HA
35-
25-
GAPDH

Fig. 3G

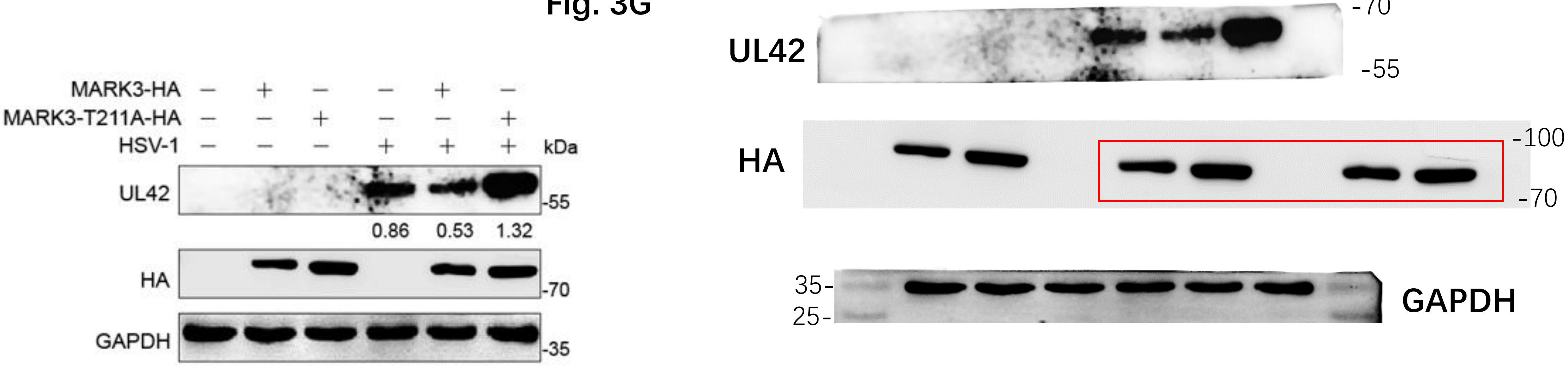
MARK3-HA − + − − + −
MARK3-T211A-HA − − + − − +
HSV-1 − − − + + +
kDa
UL42
-55
0.86 0.53 1.32
HA
-70
GAPDH
-35
UL42
-70
-55
HA
-100
-70
35-
25-
GAPDH

Fig. 3H 3I

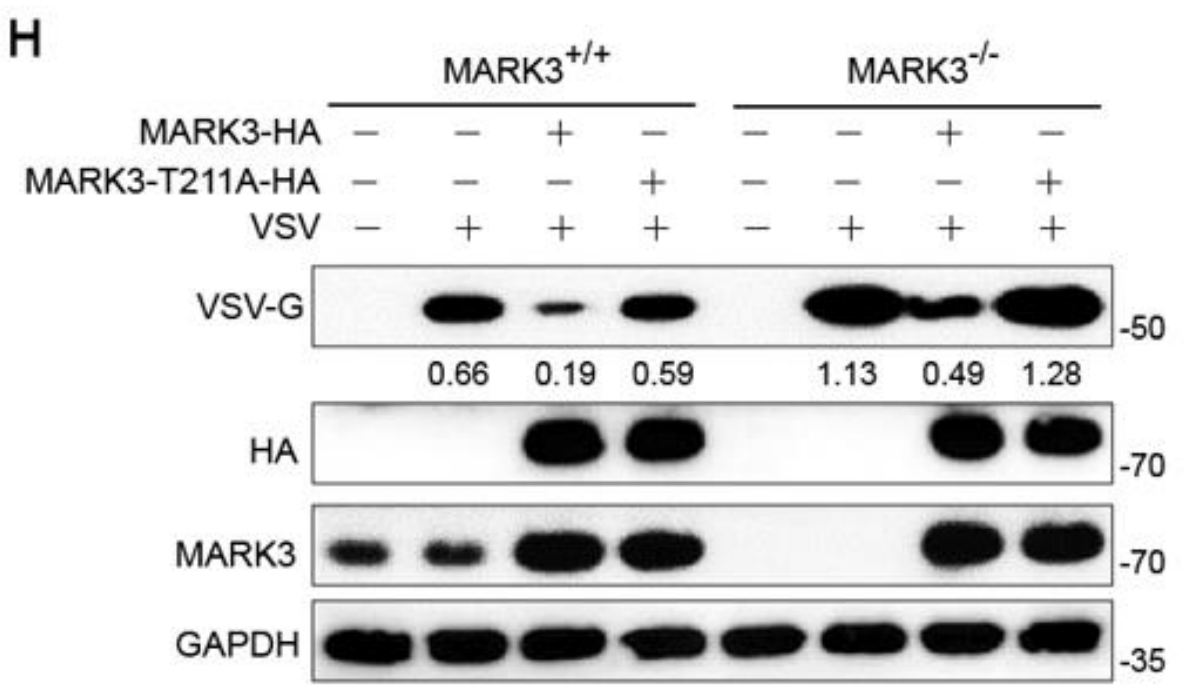
H
MARK3+/+
MARK3-/-
MARK3-HA
MARK3-T211A-HA
VSV
VSV-G
0.66
0.19
0.59
1.13
0.49
1.28
HA
MARK3
GAPDH
-50
-70
-70
-35

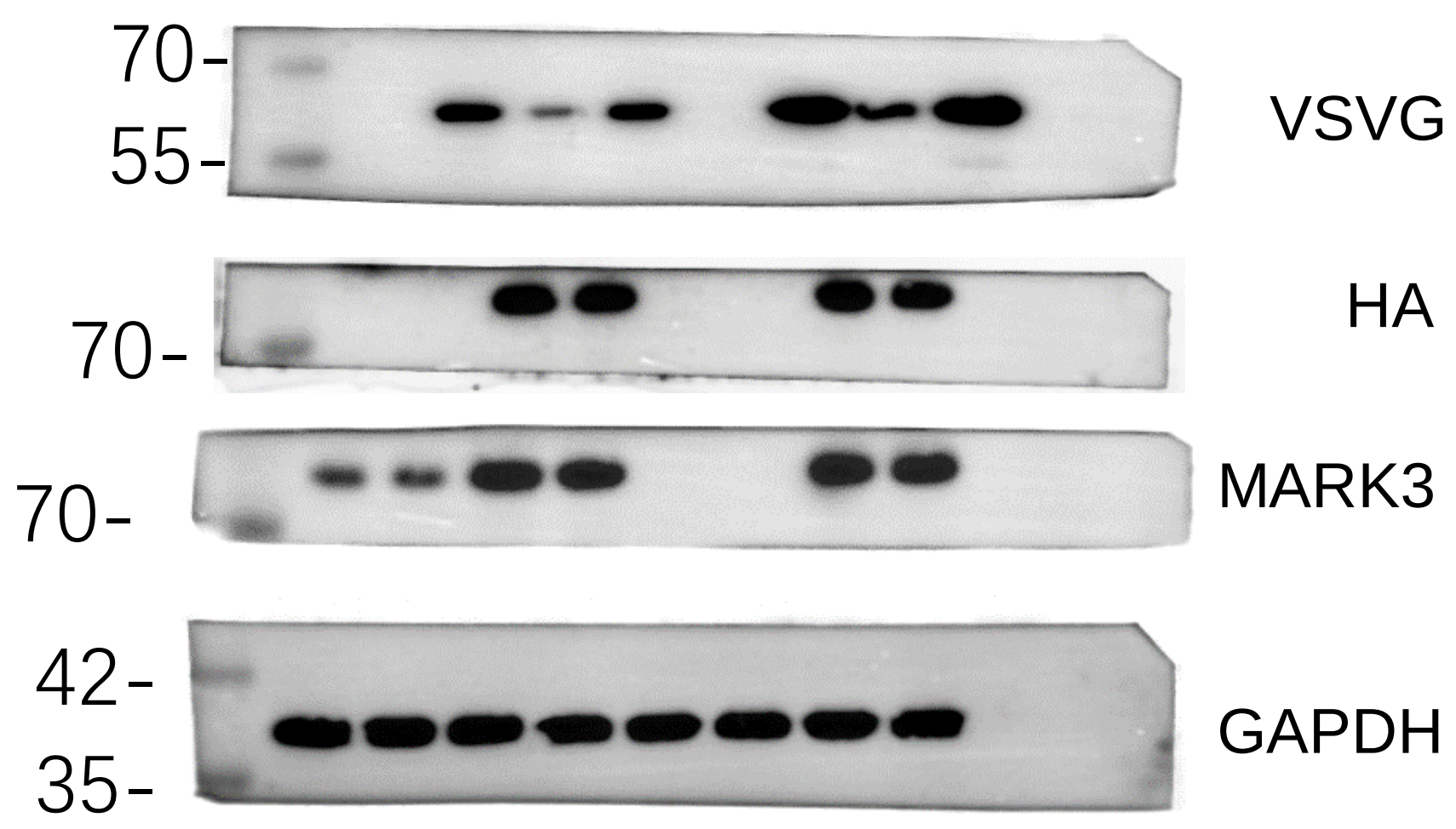
70-
55-
VSVG
HA
70-
MARK3
70-
42-
35-
GAPDH

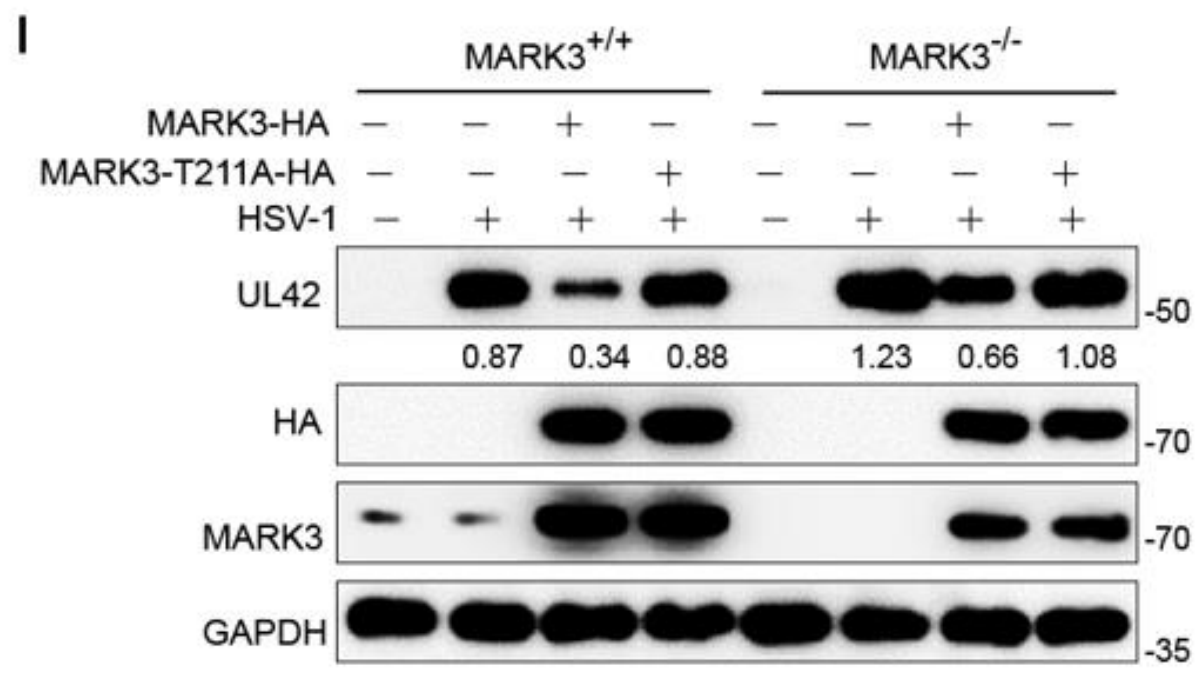
I
MARK3+/+
MARK3-/-
MARK3-HA
MARK3-T211A-HA
HSV-1
UL42
0.87
0.34
0.88
1.23
0.66
1.08
HA
MARK3
GAPDH
-50
-70
-70
-35

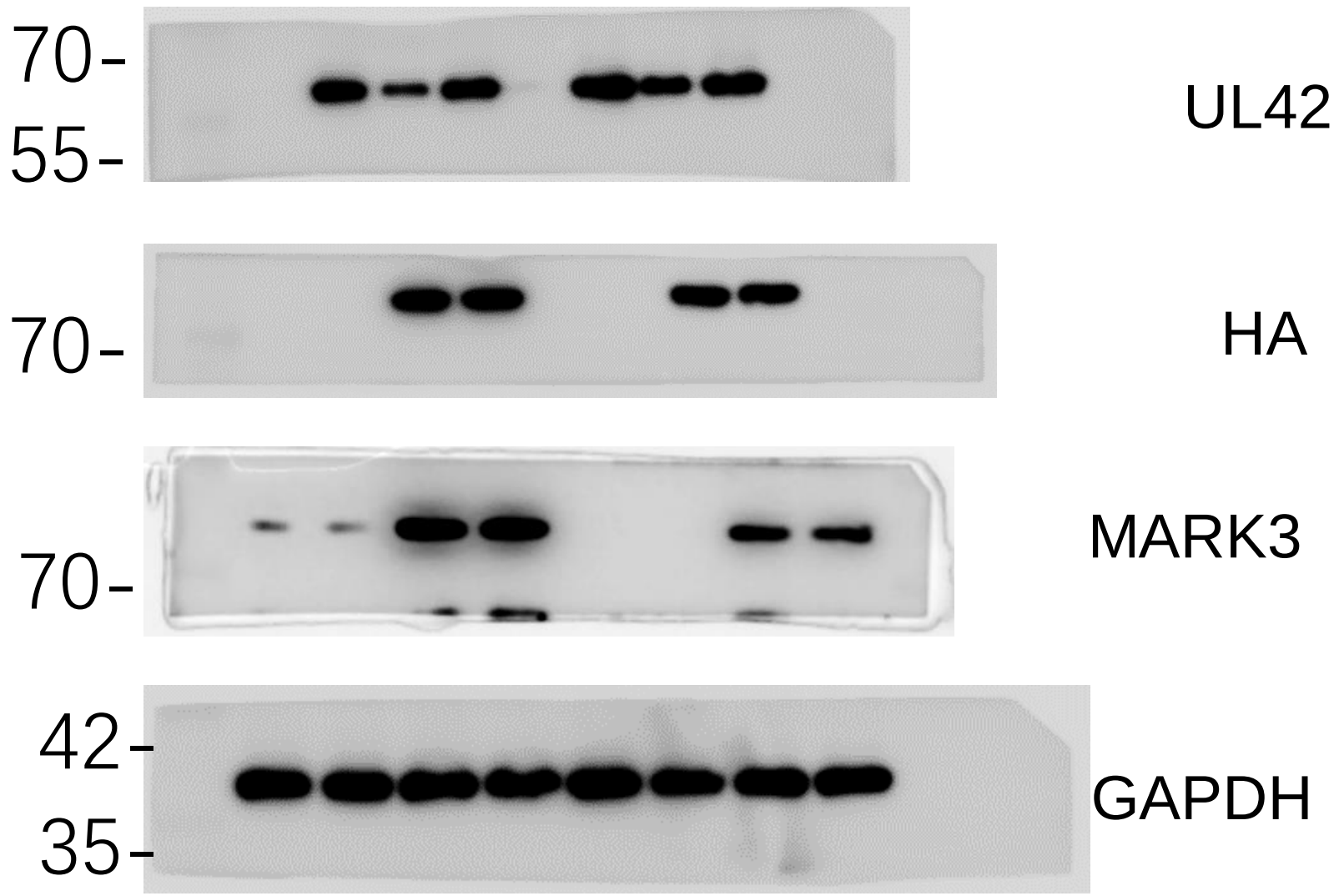
70-
55-
UL42
70-
HA
70-
MARK3
42-
35-
GAPDH

Fig. 4C

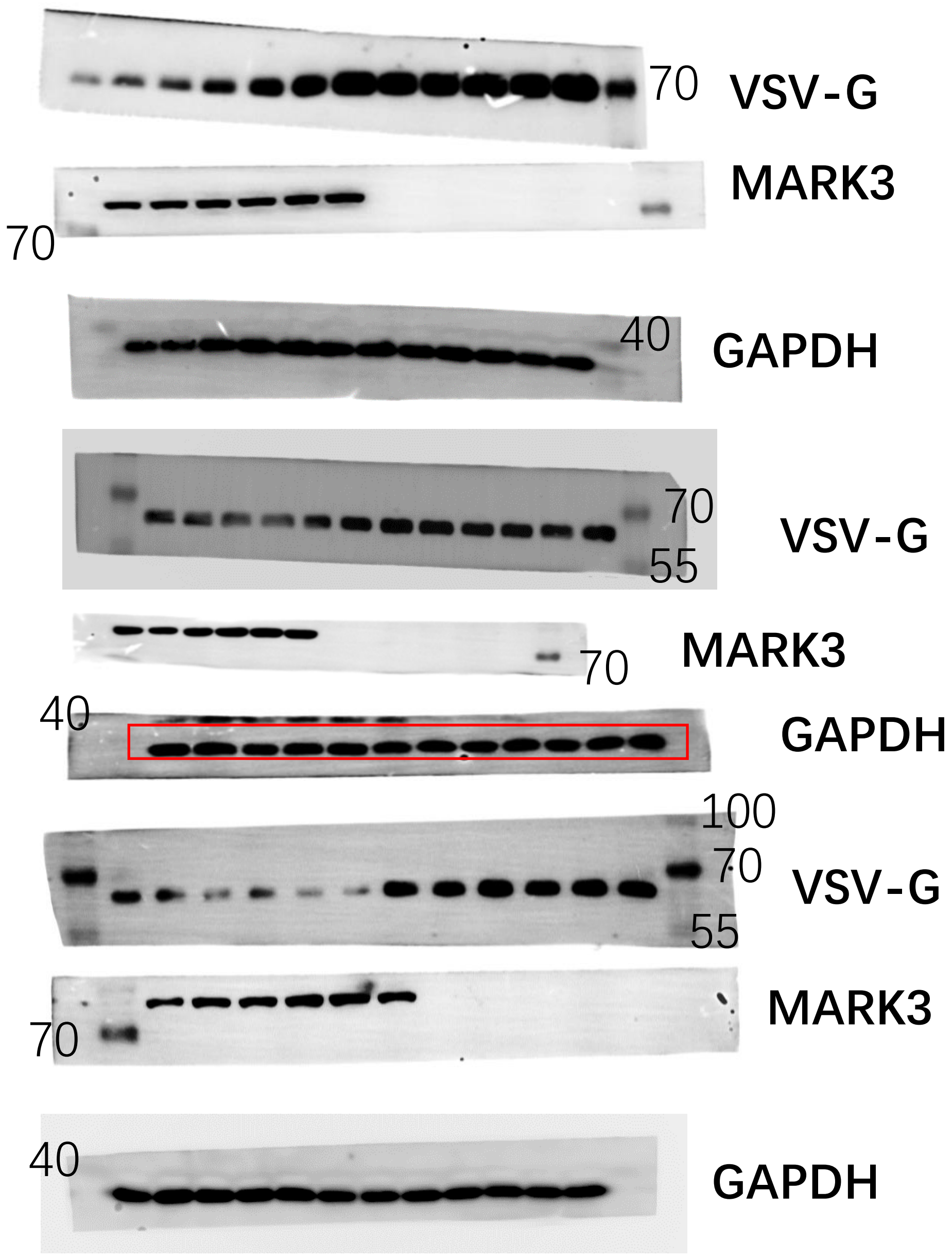

70
VSV-G
MARK3
70
40
GAPDH
70
55
VSV-G
MARK3
70
40
GAPDH
100
70
VSV-G
55
MARK3
70
40
GAPDH

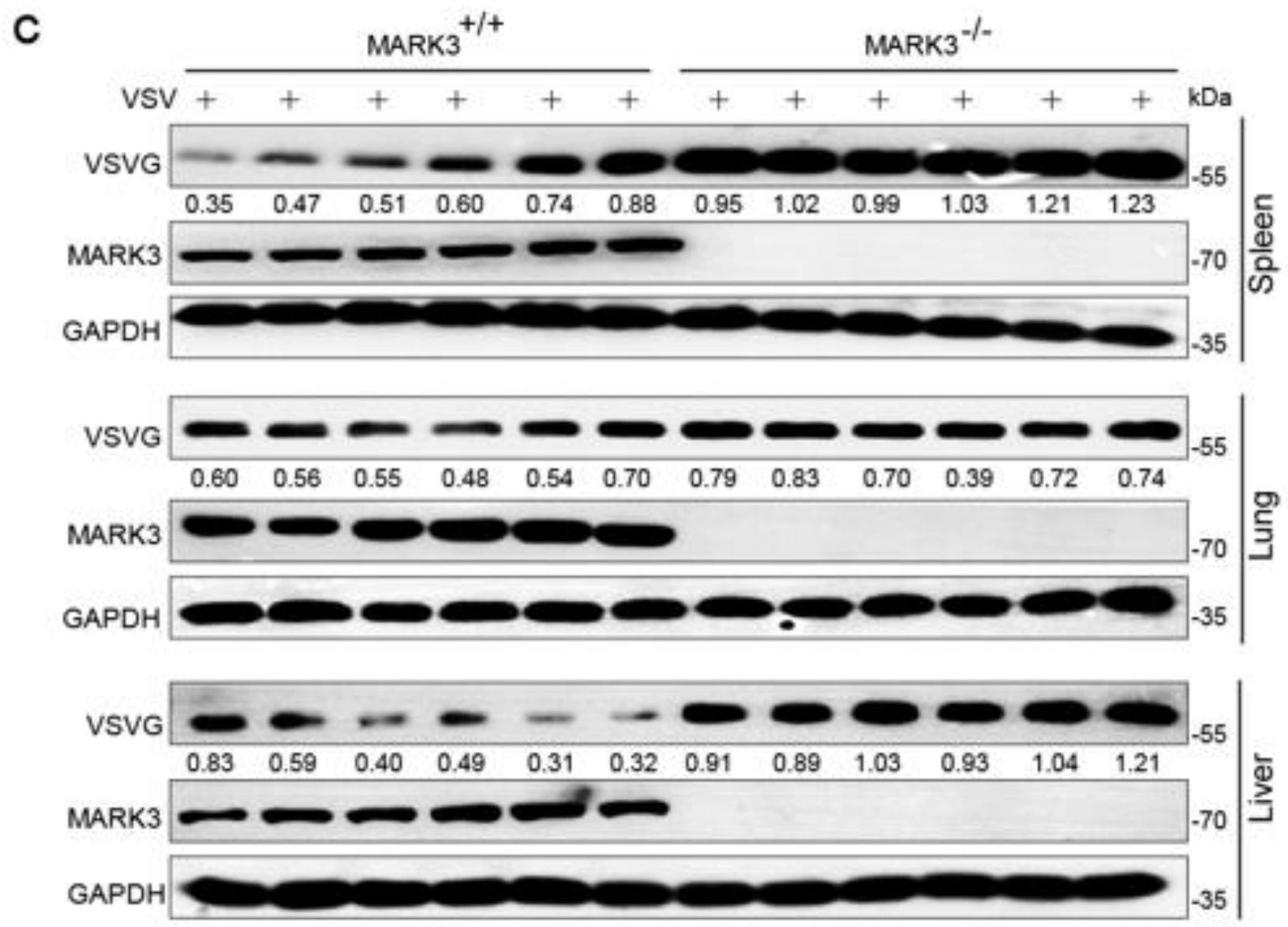

C
MARK3+/+
MARK3-/-
VSV + + + + + + + + + + + +
kDa
VSVG
0.35 0.47 0.51 0.60 0.74 0.88 0.95 1.02 0.99 1.03 1.21 1.23
MARK3
GAPDH
-55
-70
-35
Spleen
VSVG
0.60 0.56 0.55 0.48 0.54 0.70 0.79 0.83 0.70 0.39 0.72 0.74
MARK3
GAPDH
-55
-70
-35
Lung
VSVG
0.83 0.59 0.40 0.49 0.31 0.32 0.91 0.89 1.03 0.93 1.04 1.21
MARK3
GAPDH
-55
-70
-35
Liver

Fig. 5A

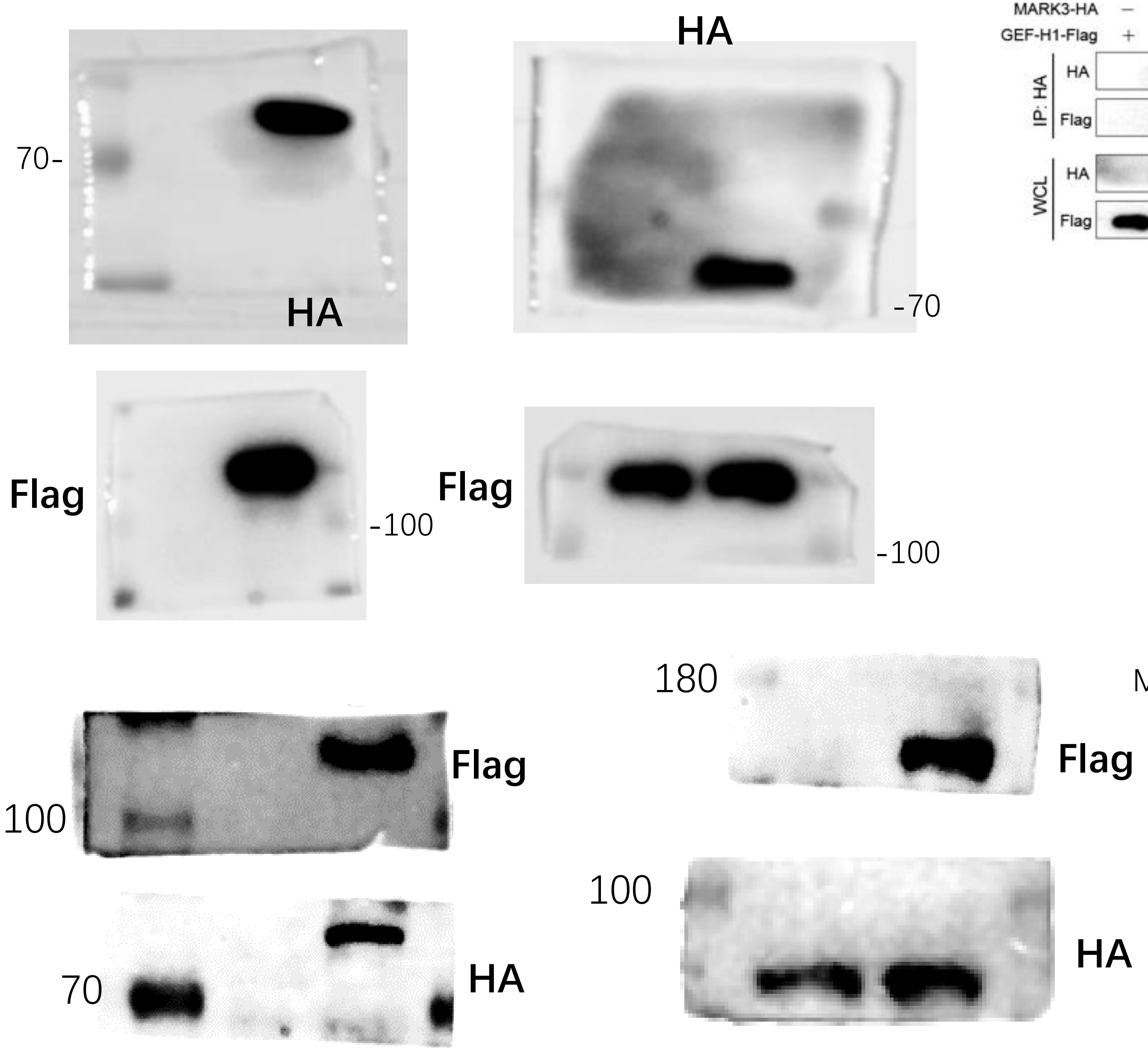
HA
70-
HA
-70
Flag
-100
Flag
-100
Flag
100
180
Flag
70
HA
100
HA

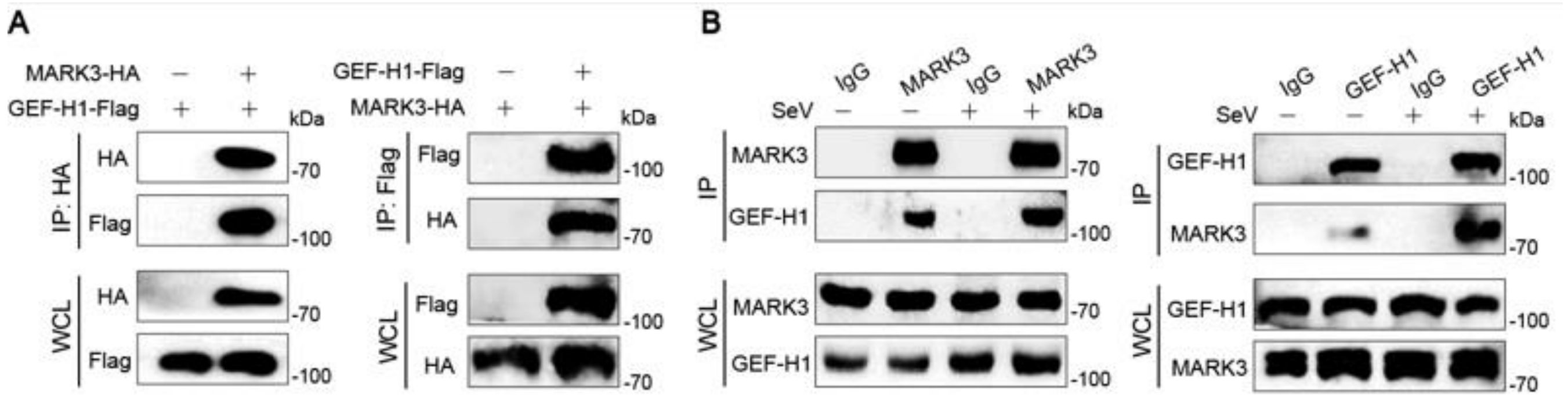
A
MARK3-HA
GEF-H1-Flag
kDa
IP: HA
HA
Flag
WCL
HA
Flag
-70
-100
GEF-H1-Flag
MARK3-HA
kDa
IP: Flag
Flag
HA
WCL
Flag
HA
-100
-70
B
IgG
MARK3
IgG
MARK3
SeV
kDa
IP
MARK3
GEF-H1
WCL
MARK3
GEF-H1
-70
-100
IgG
GEF-H1
IgG
GEF-H1
SeV
kDa
IP
GEF-H1
MARK3
WCL
GEF-H1
MARK3
-100
-70

Fig. 5B

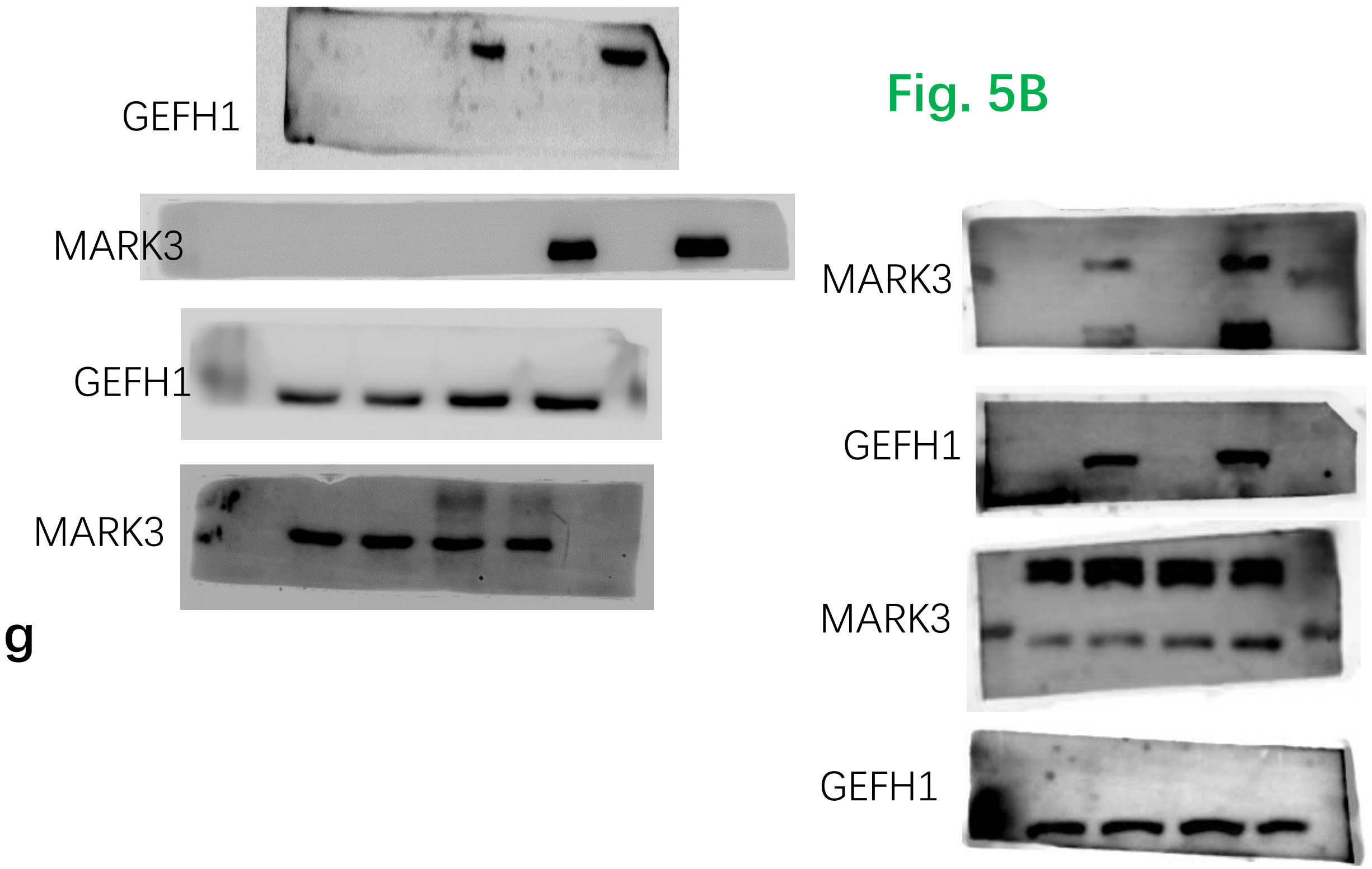
GEFH1
MARK3
GEFH1
MARK3
MARK3
GEFH1
MARK3
GEFH1

Fig. 5C

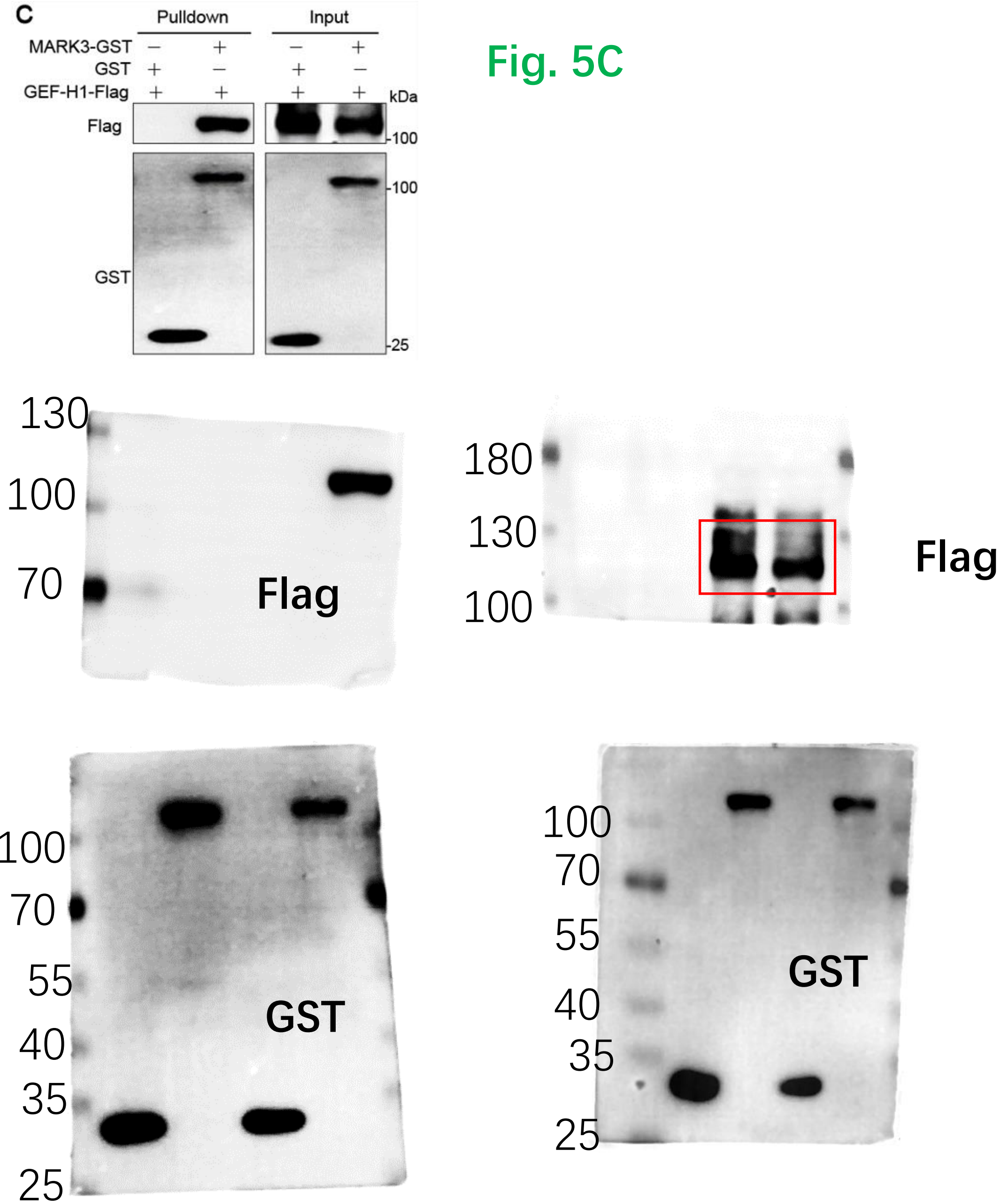
C
Pulldown
Input
MARK3-GST
GST
GEF-H1-Flag
kDa
Flag
GST
100
25
130
70
180
Flag
GST
55
40
35

Fig. 5D

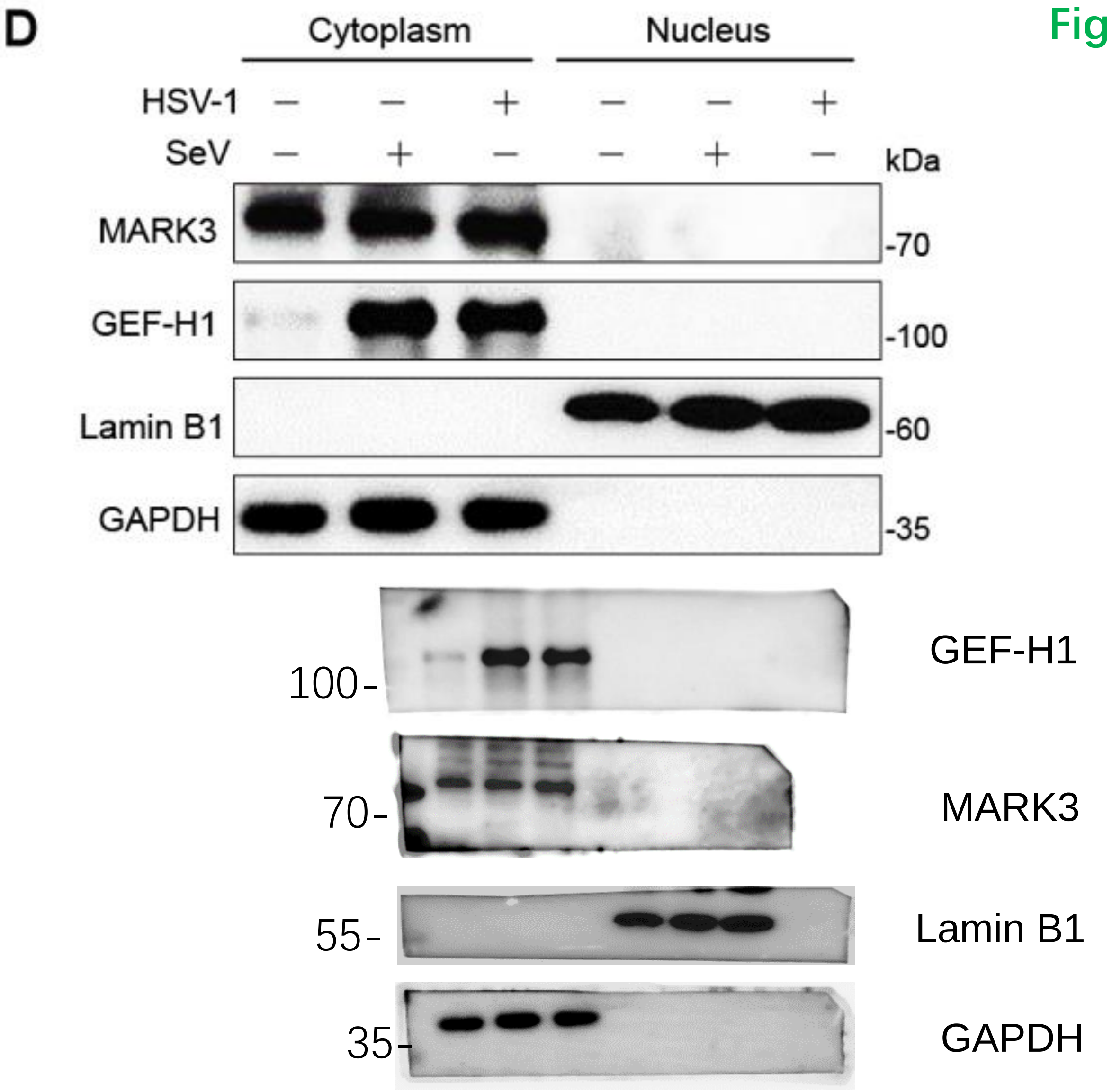
D
Cytoplasm
Nucleus
HSV-1
SeV
kDa
MARK3
GEF-H1
Lamin B1
GAPDH
-70
-100
-60
-35
100-
70-
55-
35-

Fig. 5G

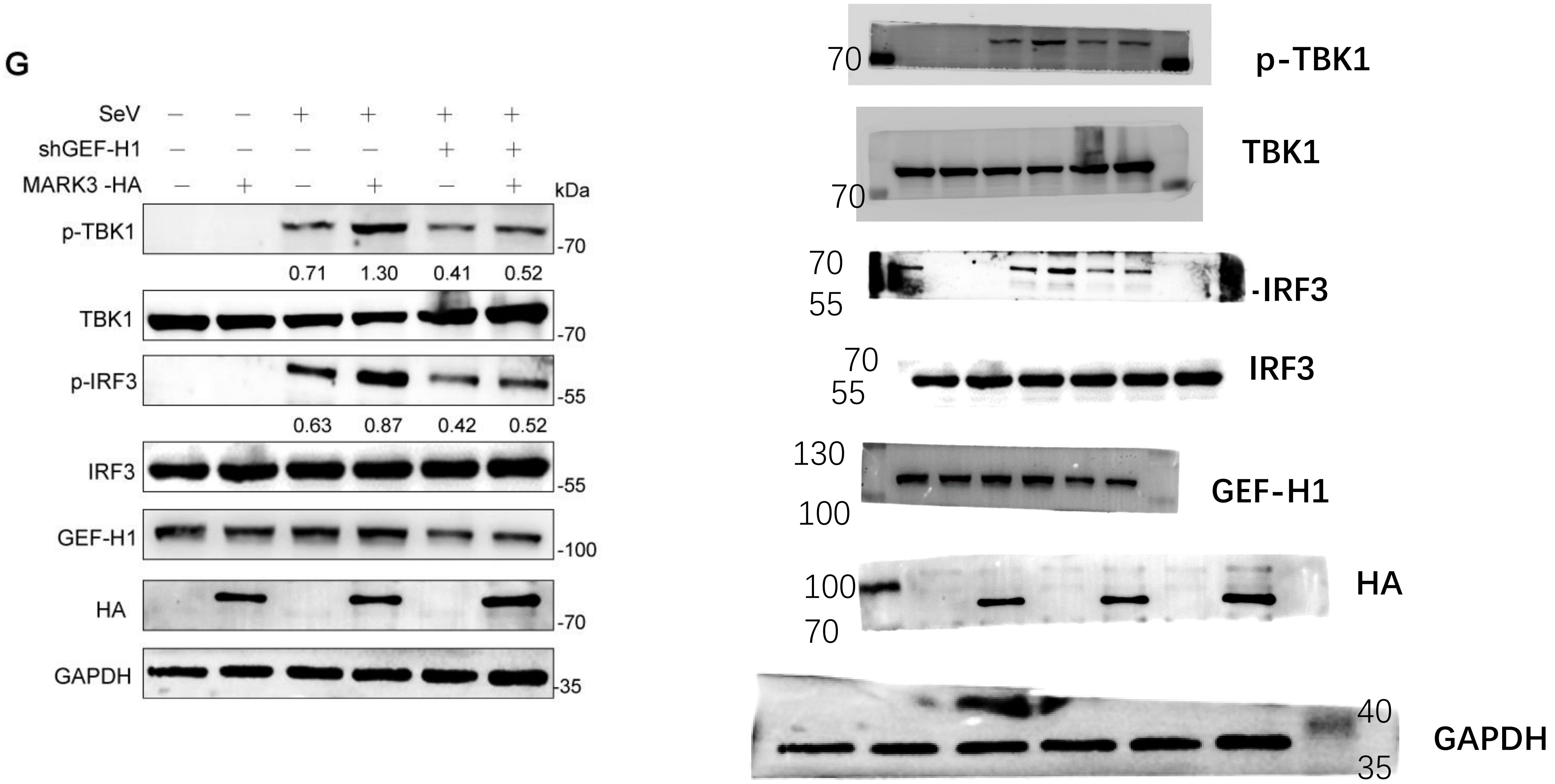
G
SeV − − + + + +
shGEF-H1 − − − − + +
MARK3 -HA − + − + − +
kDa
p-TBK1
-70
0.71 1.30 0.41 0.52
TBK1
-70
p-IRF3
-55
0.63 0.87 0.42 0.52
IRF3
-55
GEF-H1
-100
HA
-70
GAPDH
-35
70
p-TBK1
TBK1
70
70
55
-IRF3
70
55
IRF3
130
100
GEF-H1
100
70
HA
40
35
GAPDH

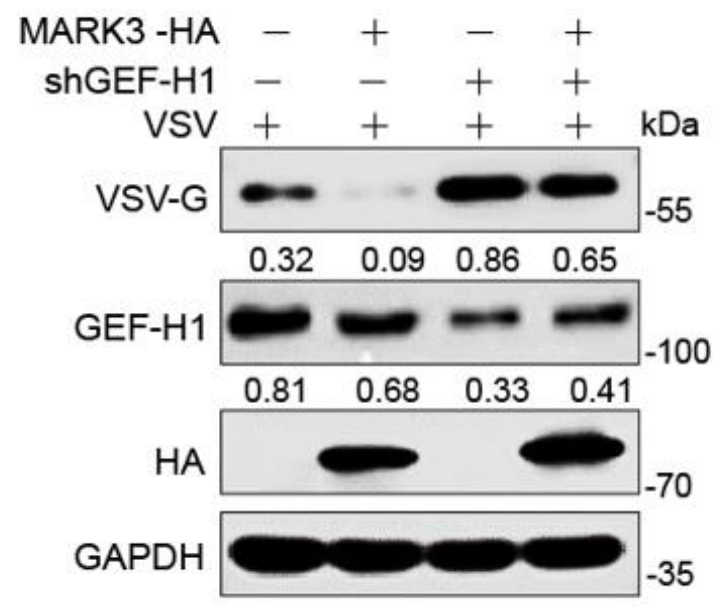

MARK3 -HA − + − +
shGEF-H1 − − + +
VSV + + + +
kDa
VSV-G
-55
0.32 0.09 0.86 0.65
GEF-H1
-100
0.81 0.68 0.33 0.41
HA
-70
GAPDH
-35

Fig. 5H

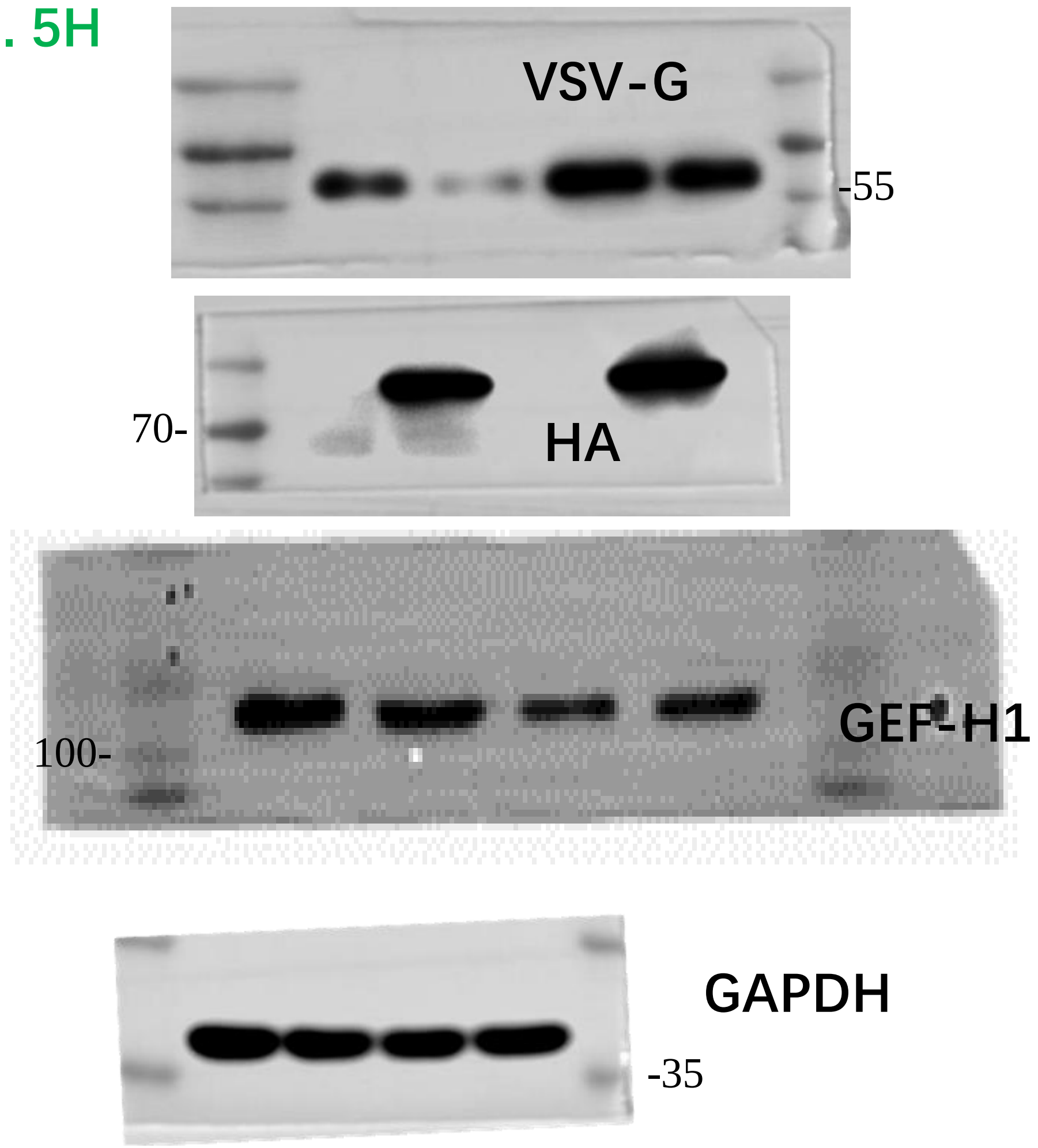

VSV-G
-55
70-
HA
100-
GEF-H1
GAPDH
-35

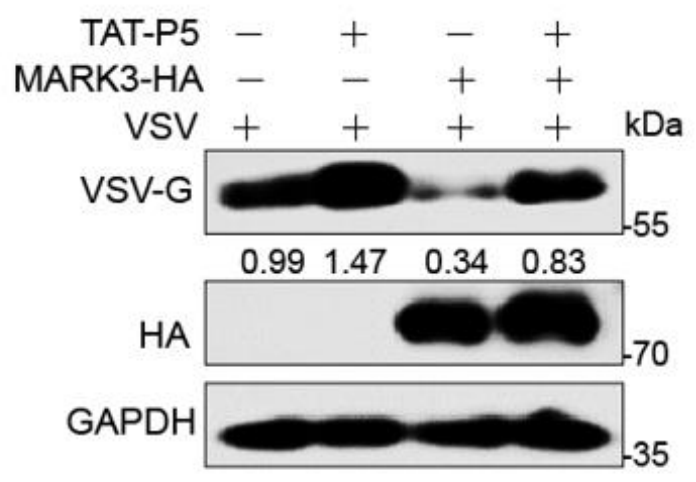

TAT-P5 − + − +
MARK3-HA − − + +
VSV + + + +
kDa
VSV-G
55
0.99 1.47 0.34 0.83
HA
70
GAPDH
35

Fig. 5I

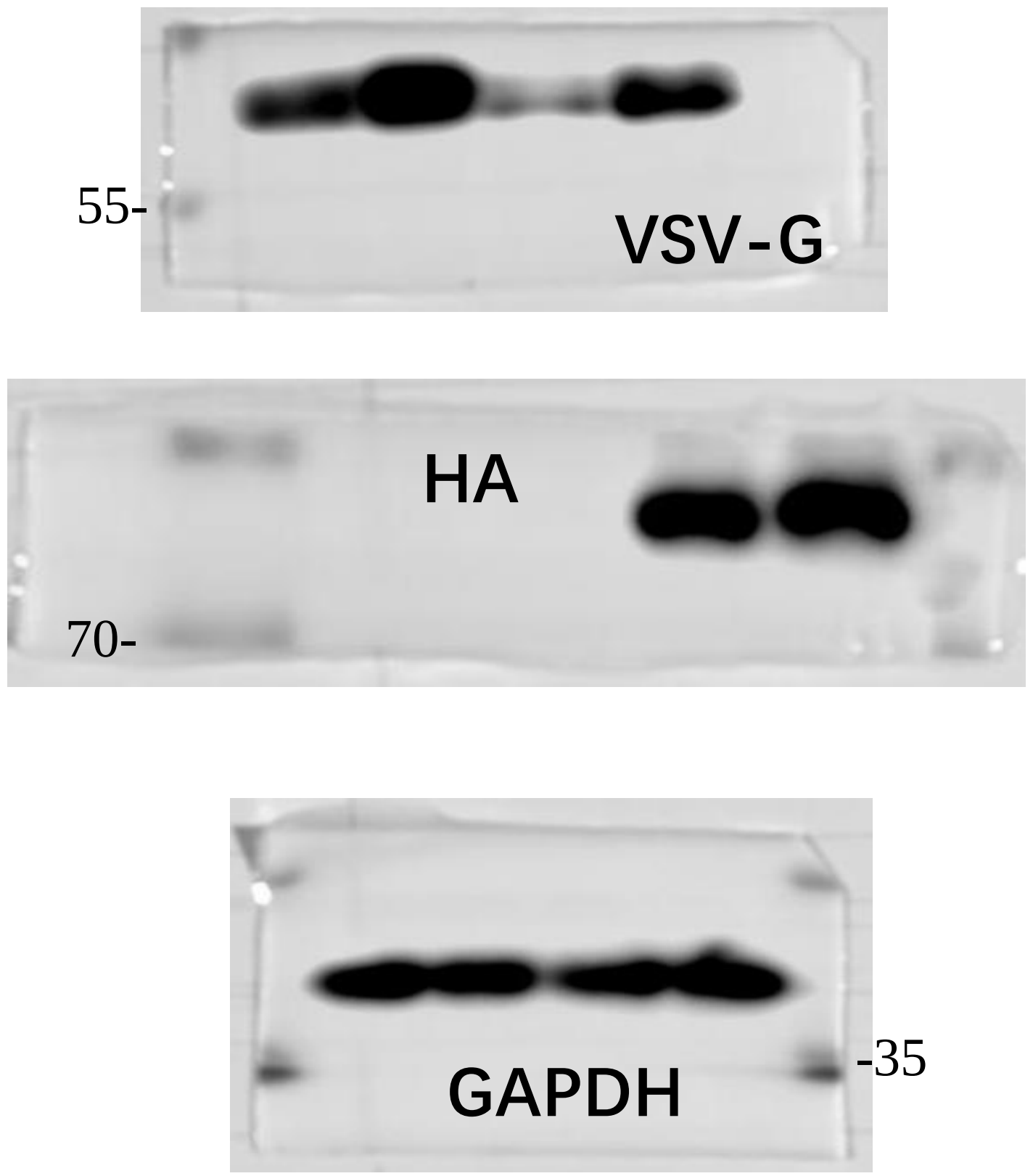

55-
VSV-G
HA
70-
GAPDH
-35

Fig. 6J

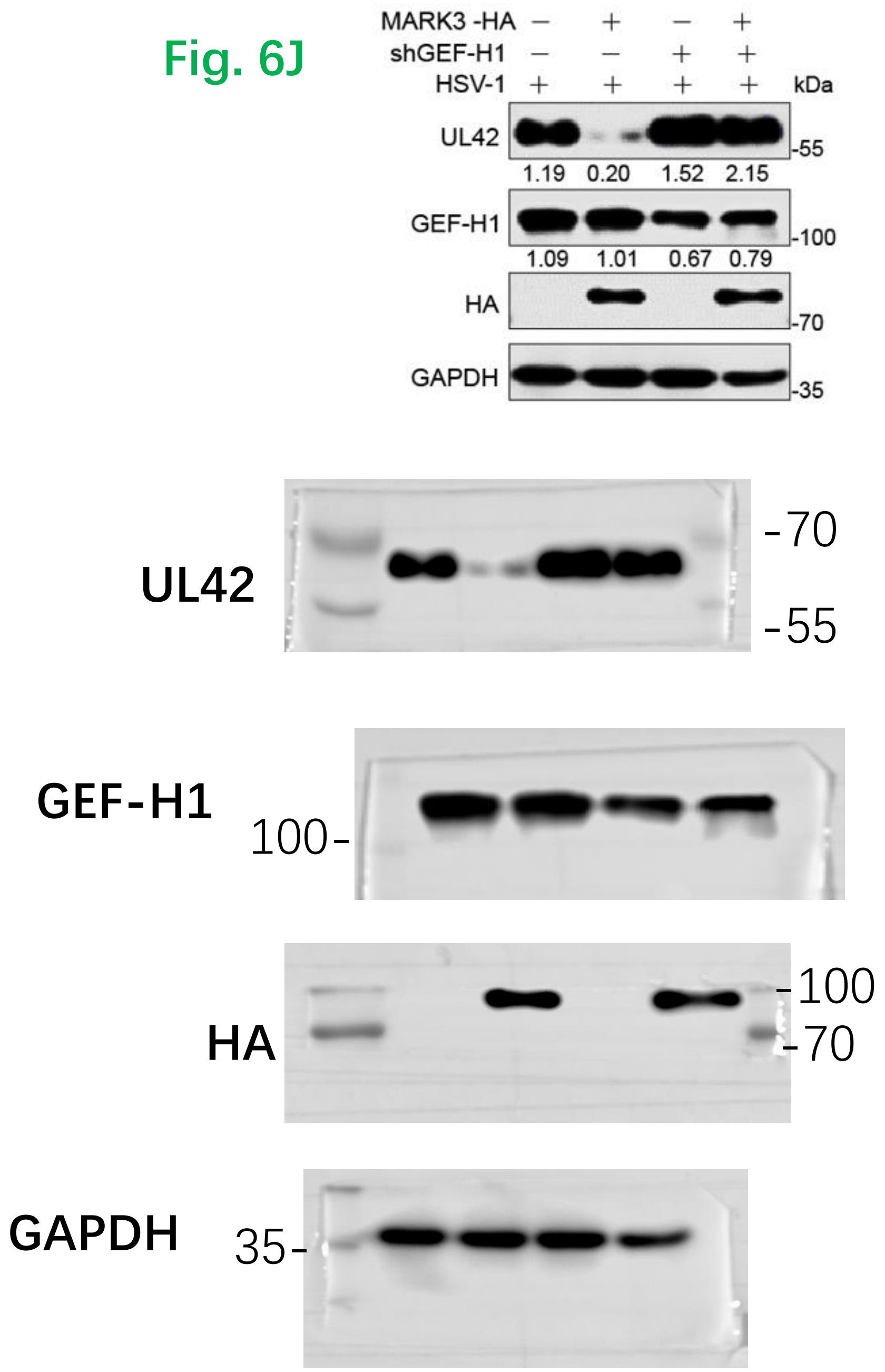

MARK3 -HA – + – +
shGEF-H1 – – + +
HSV-1 + + + +
kDa
UL42
-55
1.19 0.20 1.52 2.15
GEF-H1
-100
1.09 1.01 0.67 0.79
HA
-70
GAPDH
-35
UL42
-70
-55
GEF-H1
100-
HA
-100
-70
GAPDH
35-

Fig. 6K

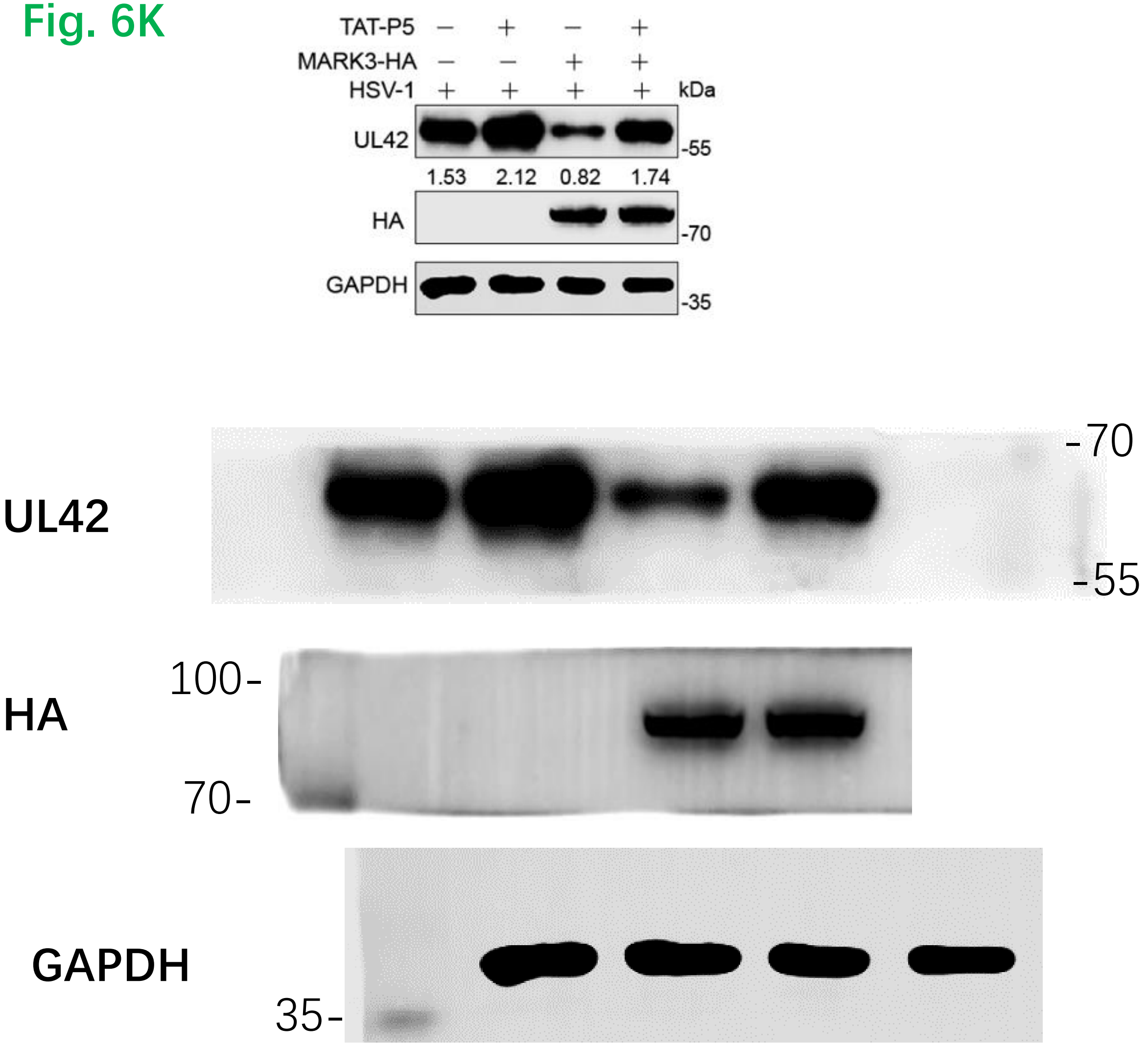

TAT-P5 – + – +
MARK3-HA – – + +
HSV-1 + + + +
kDa
UL42
-55
1.53 2.12 0.82 1.74
HA
-70
GAPDH
-35
UL42
-70
-55
HA
100-
70-
GAPDH
35-

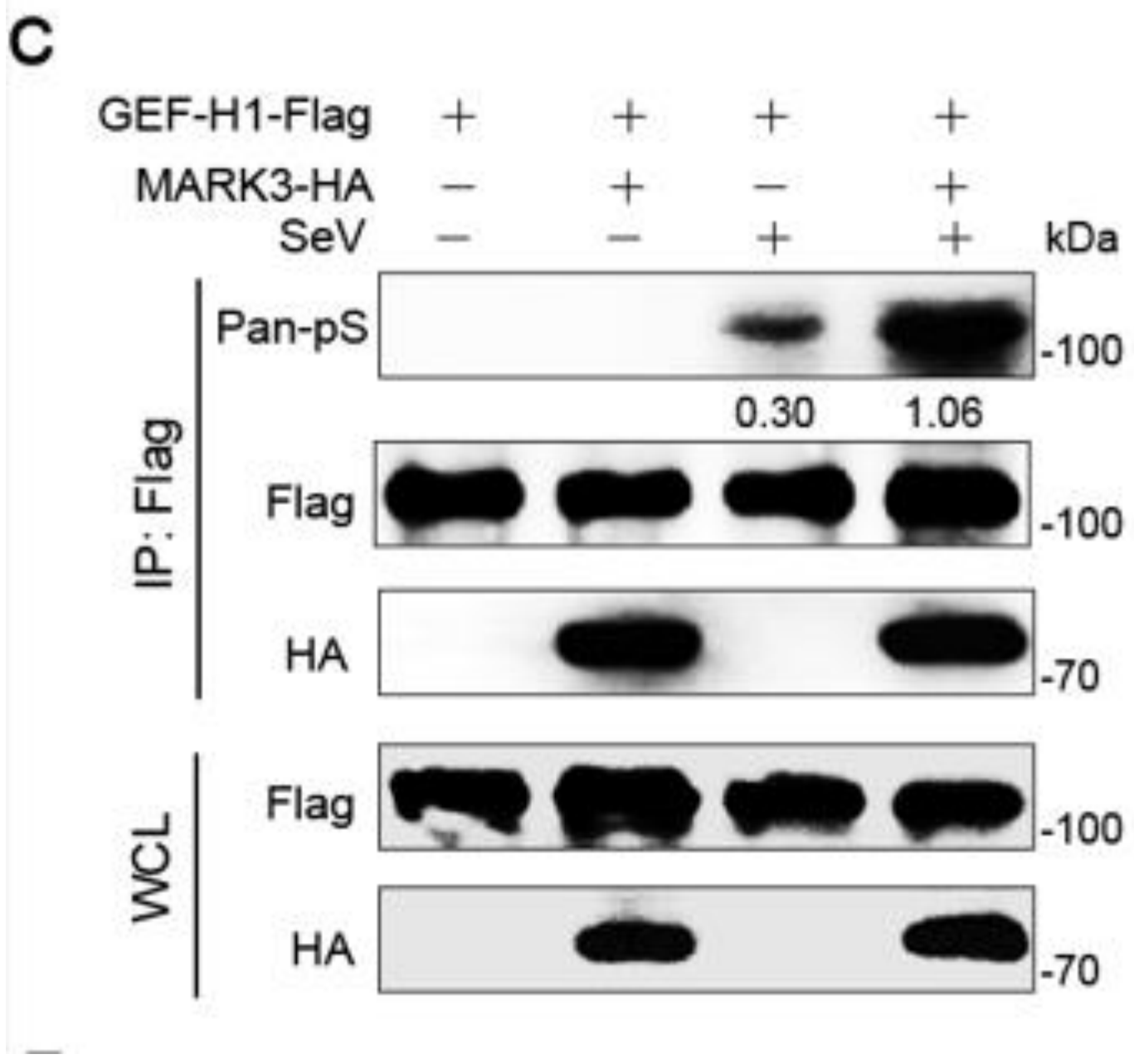
C
GEF-H1-Flag + + + +
MARK3-HA − + − +
SeV − − + +
kDa
IP: Flag
Pan-pS
-100
0.30
1.06
Flag
-100
HA
-70
WCL
Flag
-100
HA
-70

Fig. 6C

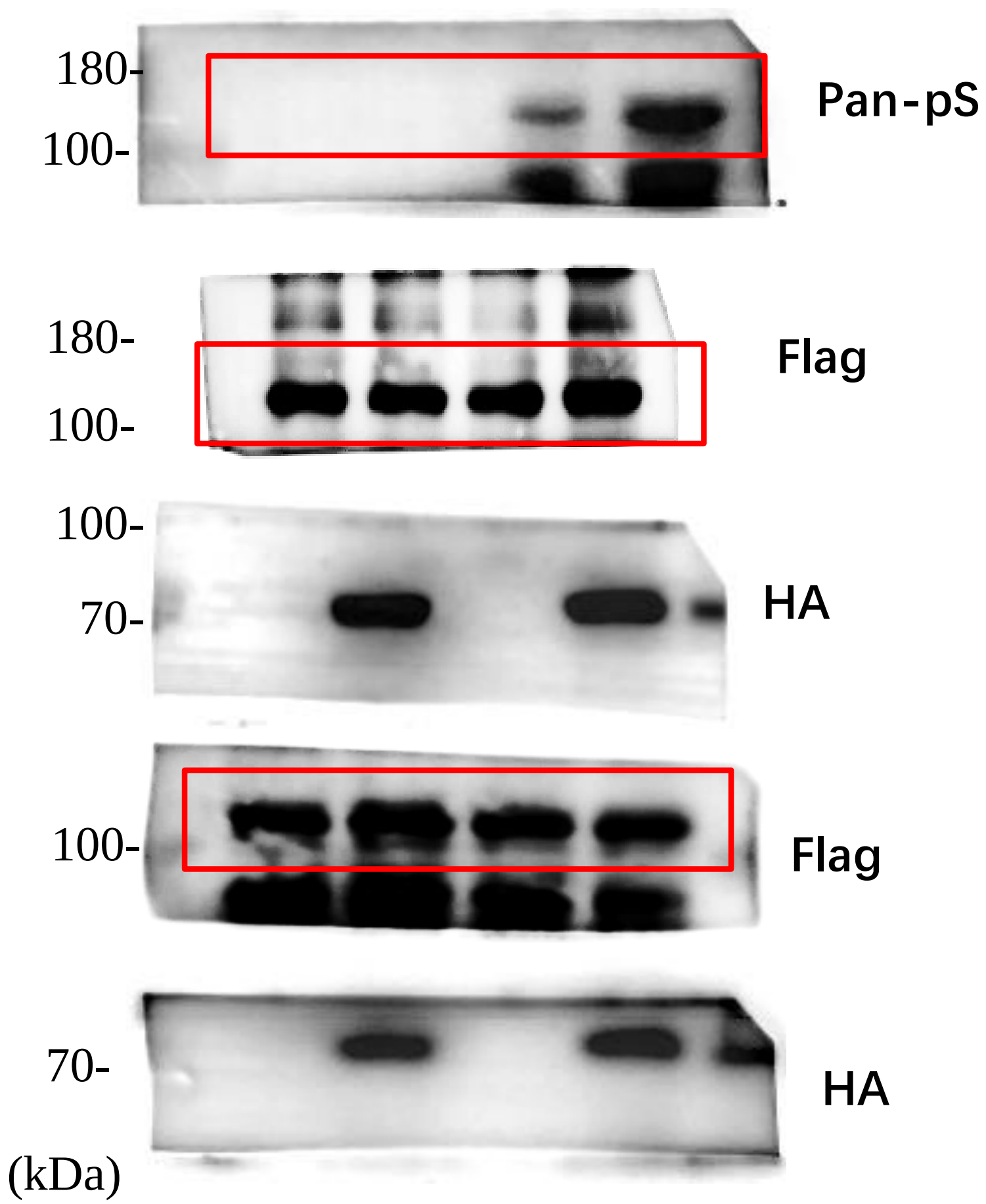
180-
100-
Pan-pS
180-
100-
Flag
100-
70-
HA
100-
Flag
70-
HA
(kDa)

Fig. 6D-1

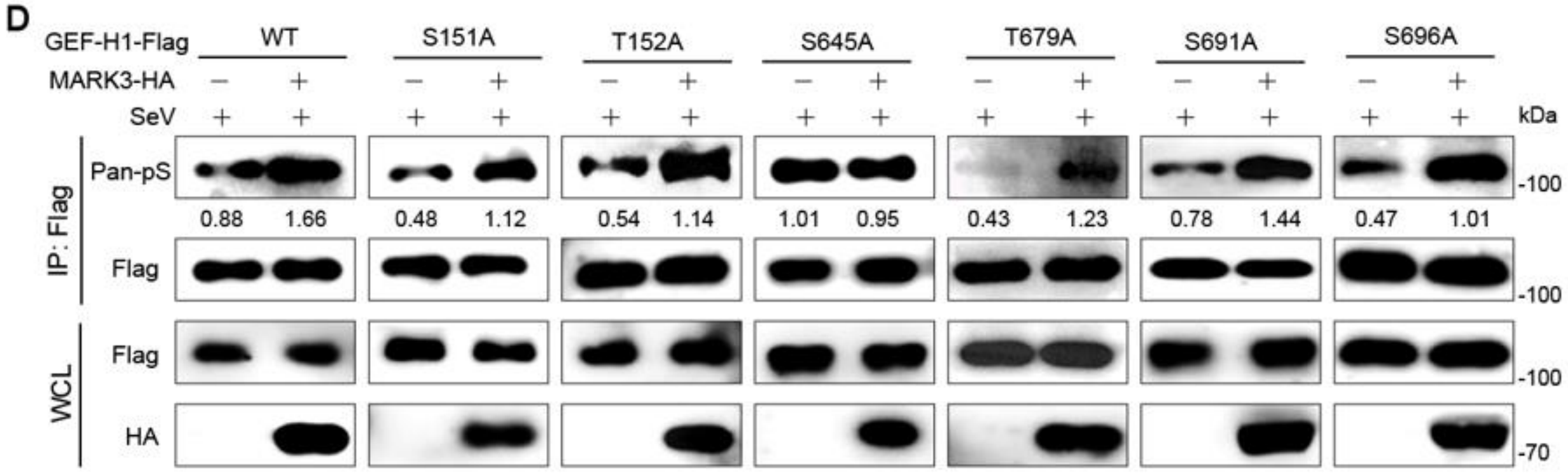

D
GEF-H1-Flag
WT
S151A
T152A
S645A
T679A
S691A
S696A
MARK3-HA
SeV
kDa
IP: Flag
Pan-pS
0.88 1.66
0.48 1.12
0.54 1.14
1.01 0.95
0.43 1.23
0.78 1.44
0.47 1.01
Flag
WCL
Flag
HA
-100
-100
-100
-70

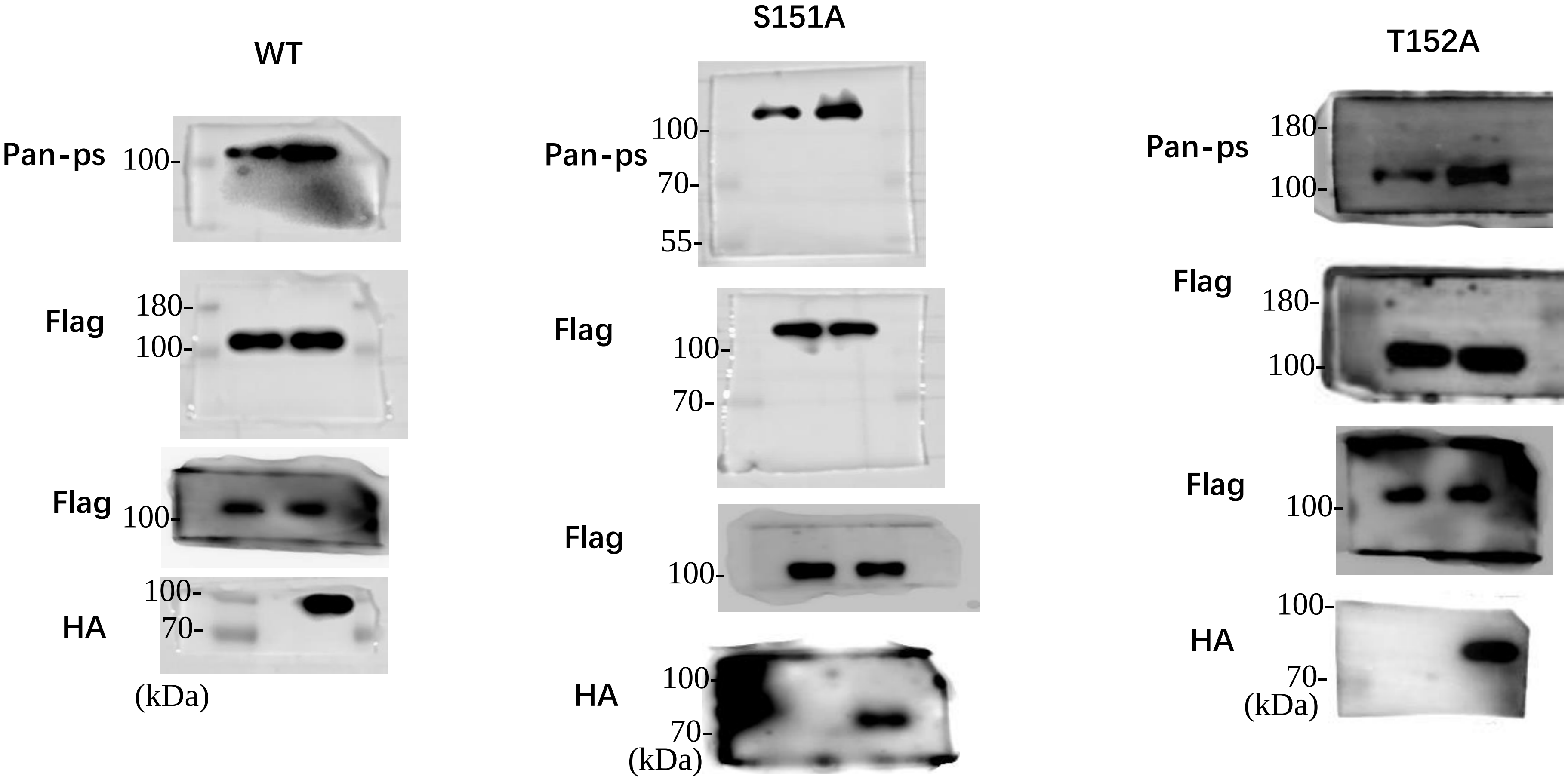

WT
S151A
T152A
Pan-ps
Flag
Flag
HA
180-
100-
70-
55-
(kDa)

Fig. 6D-2

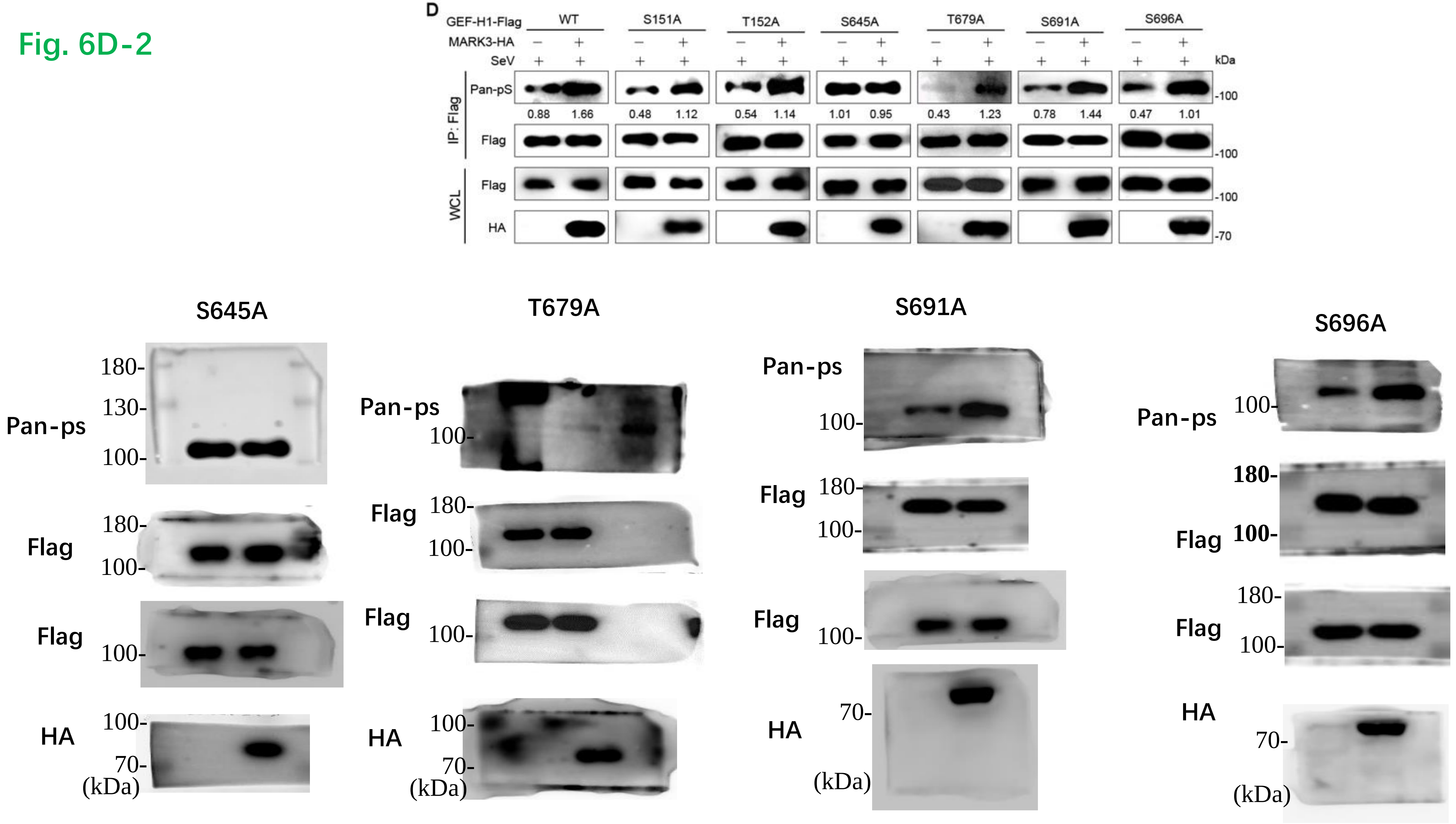
D
GEF-H1-Flag
WT
S151A
T152A
S645A
T679A
S691A
S696A
MARK3-HA
SeV
kDa
IP: Flag
Pan-pS
0.88 1.66 0.48 1.12 0.54 1.14 1.01 0.95 0.43 1.23 0.78 1.44 0.47 1.01
Flag
WCL
Flag
HA
-100
-100
-100
-70
S645A
T679A
S691A
S696A
Pan-ps
Flag
Flag
HA
180-
130-
100-
70-
(kDa)

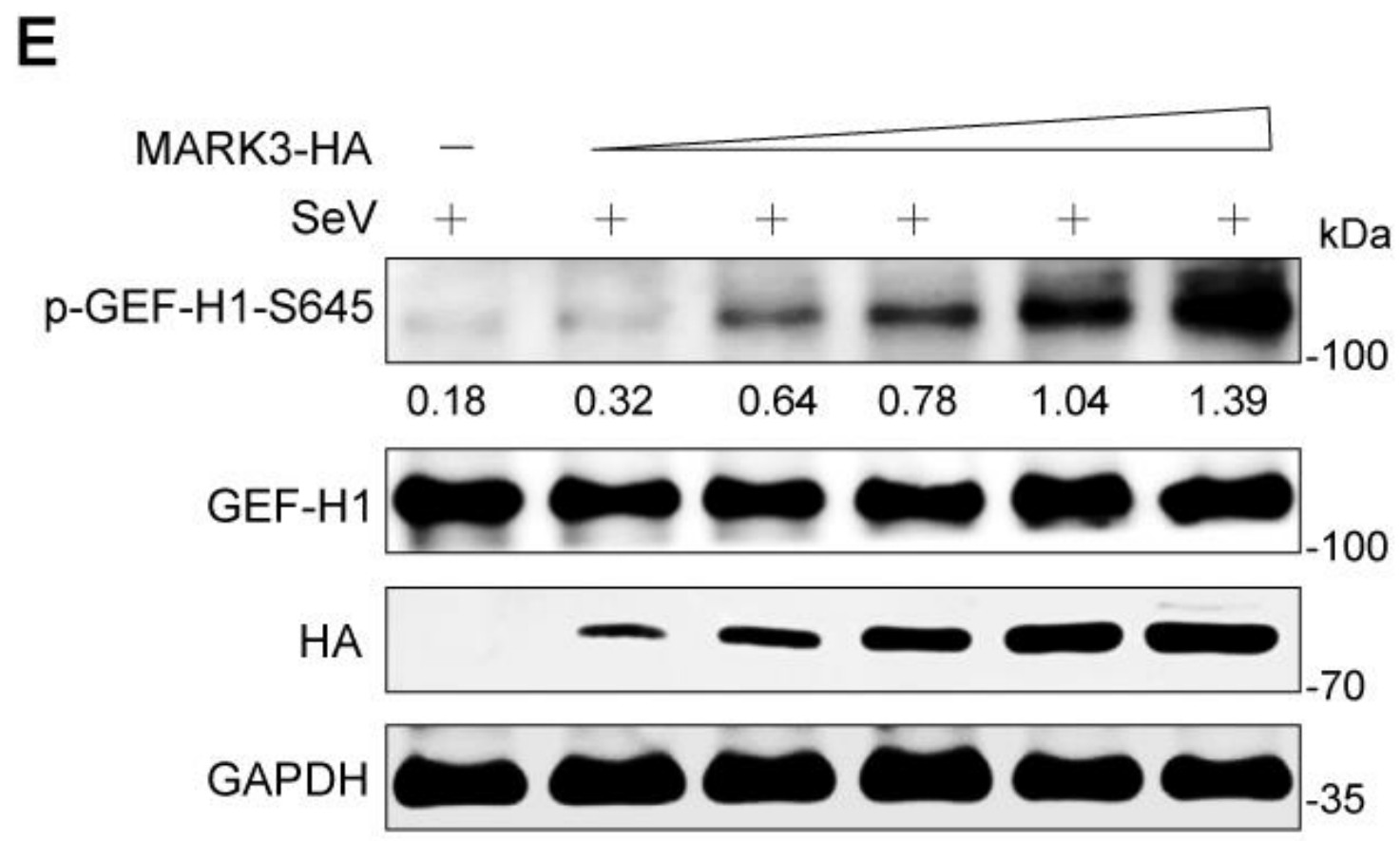
E
MARK3-HA
SeV
kDa
p-GEF-H1-S645
-100
0.18 0.32 0.64 0.78 1.04 1.39
GEF-H1
-100
HA
-70
GAPDH
-35

Fig. 6E

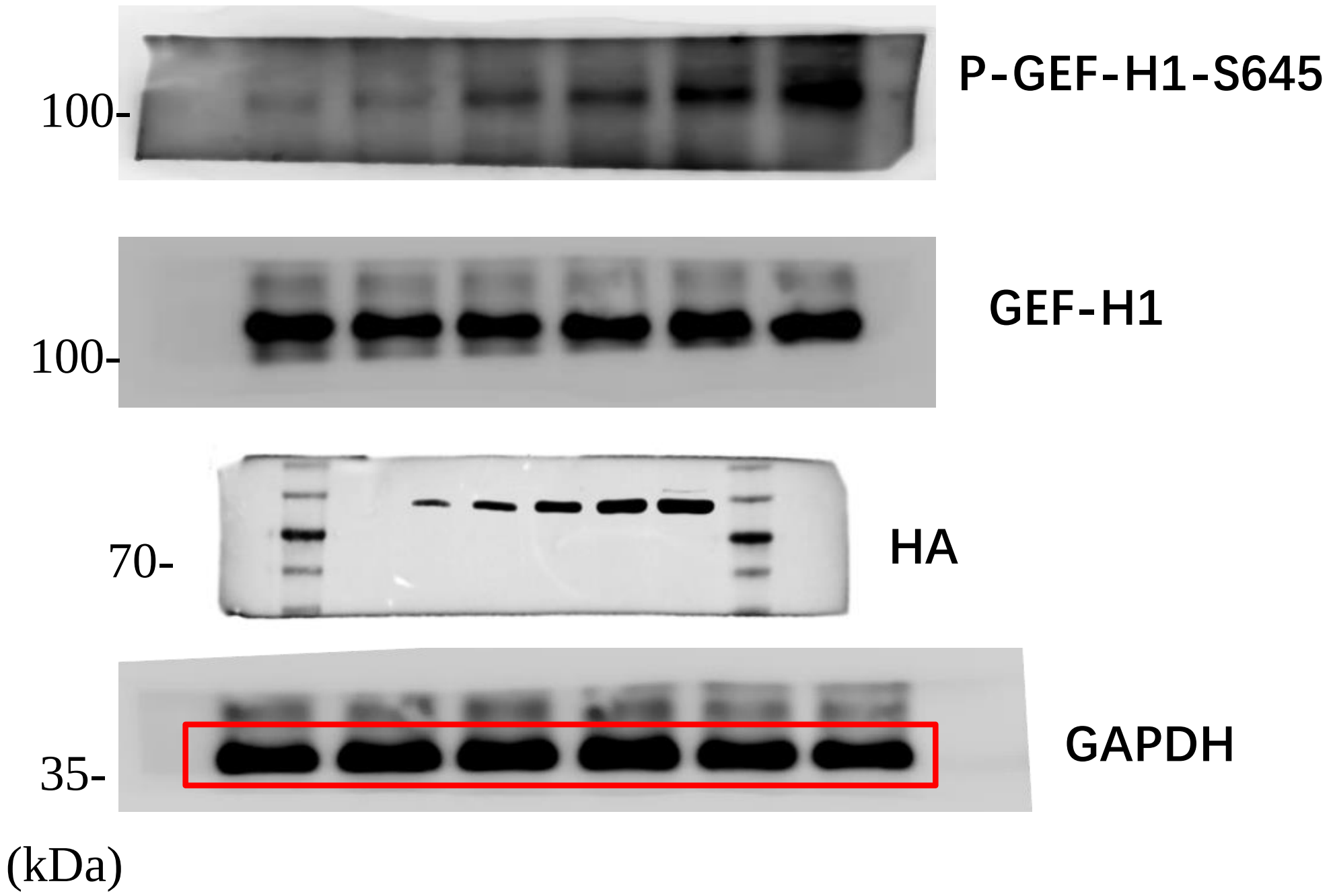
100-
P-GEF-H1-S645
100-
GEF-H1
70-
HA
35-
GAPDH
(kDa)

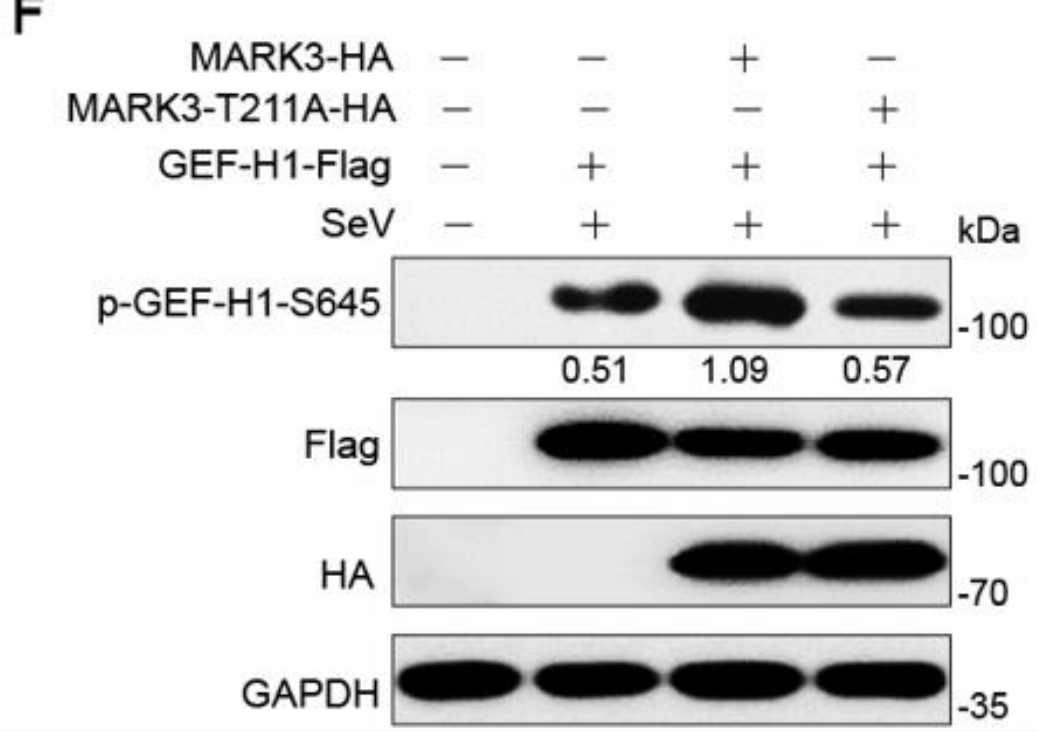
MARK3-HA
MARK3-T211A-HA
GEF-H1-Flag
SeV
kDa
p-GEF-H1-S645
-100
0.51 1.09 0.57
Flag
-100
HA
-70
GAPDH
-35

Fig. 6F

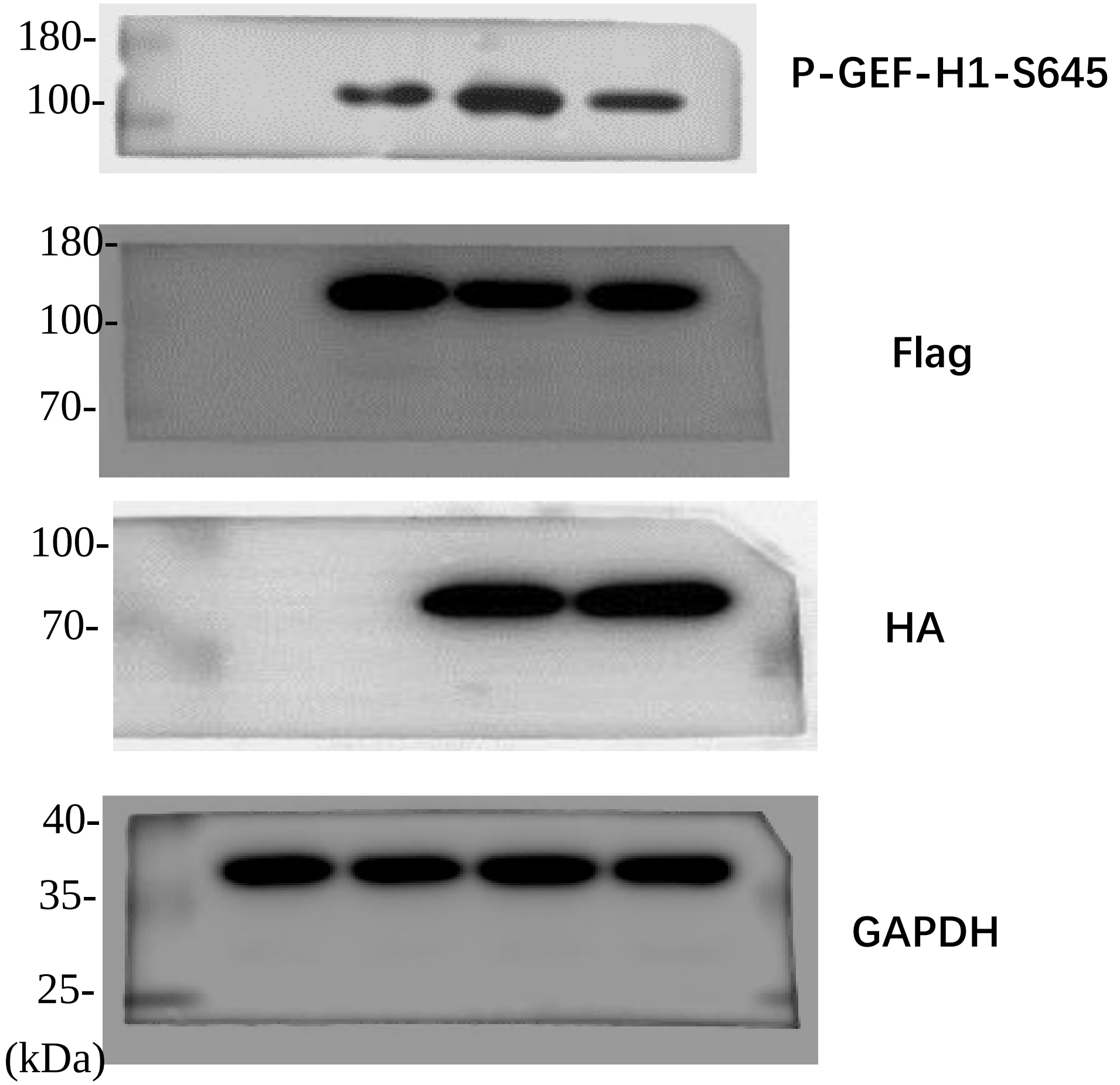
180-
100-
P-GEF-H1-S645
180-
100-
70-
Flag
100-
70-
HA
40-
35-
25-
GAPDH
(kDa)

Fig. 6G

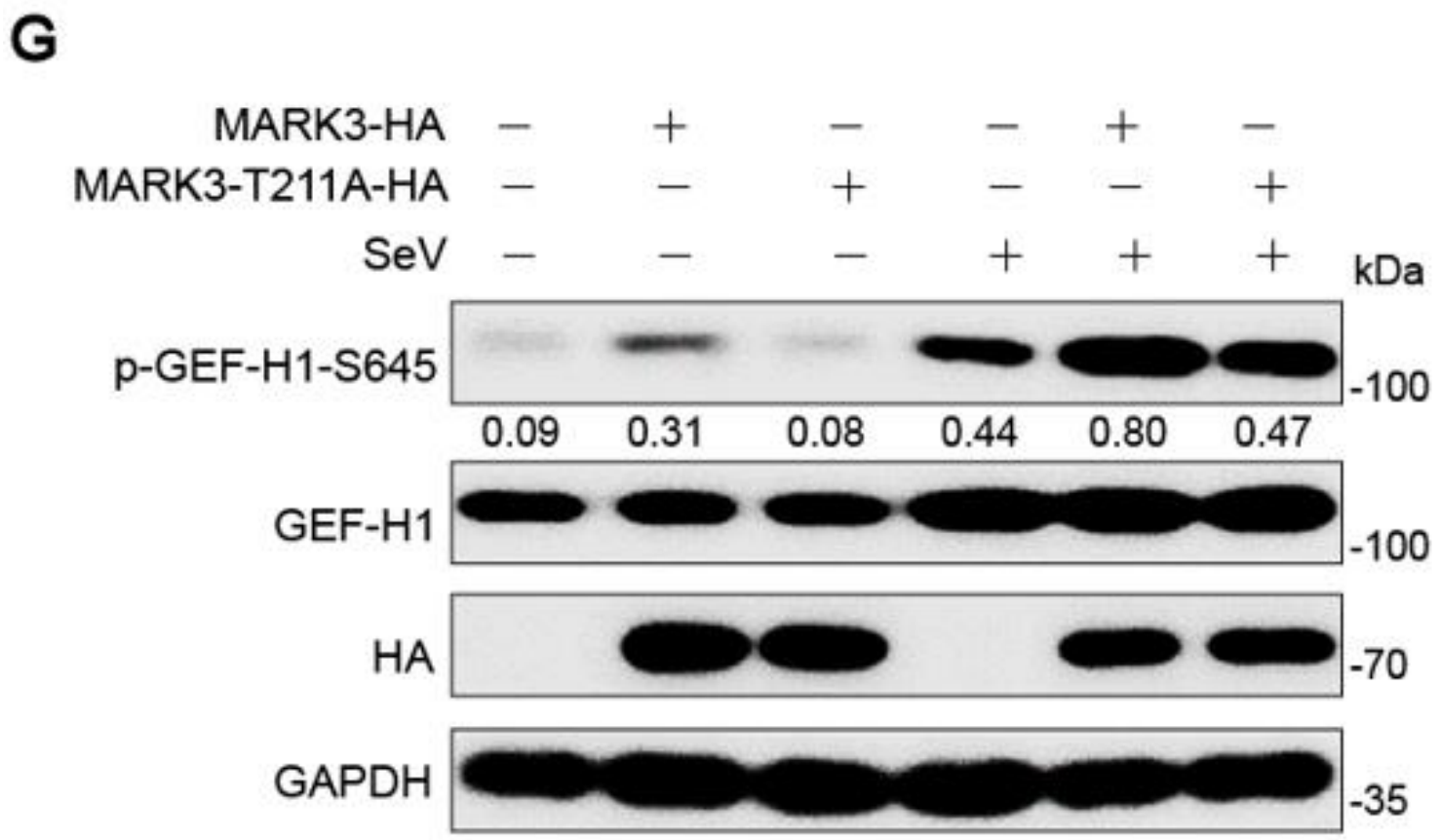
G
MARK3-HA − + − − + −
MARK3-T211A-HA − − + − − +
SeV − − − + + +
kDa
p-GEF-H1-S645
-100
0.09 0.31 0.08 0.44 0.80 0.47
GEF-H1
-100
HA
-70
GAPDH
-35

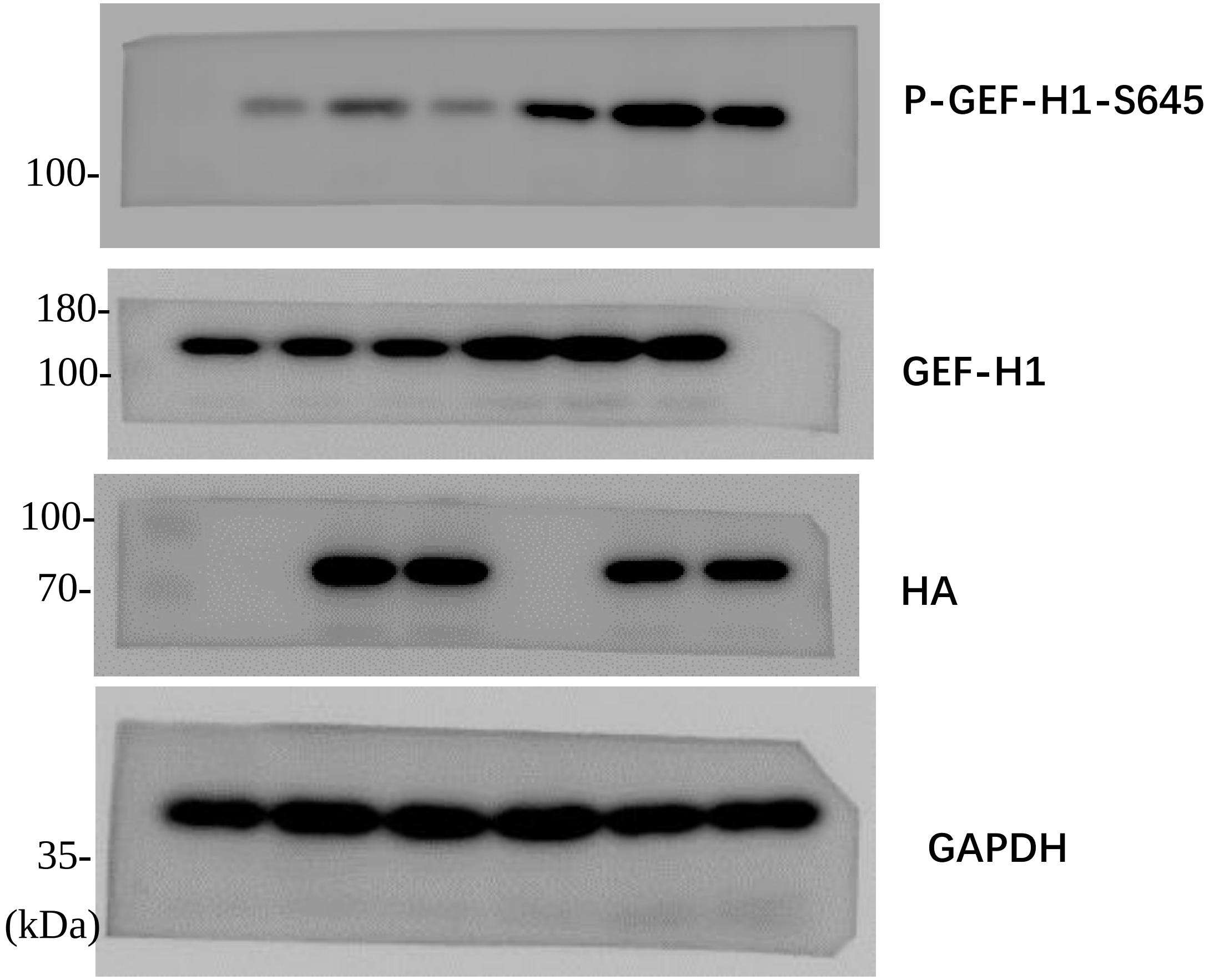
100-
P-GEF-H1-S645
180-
100-
GEF-H1
100-
70-
HA
35-
(kDa)
GAPDH

Fig. 7B

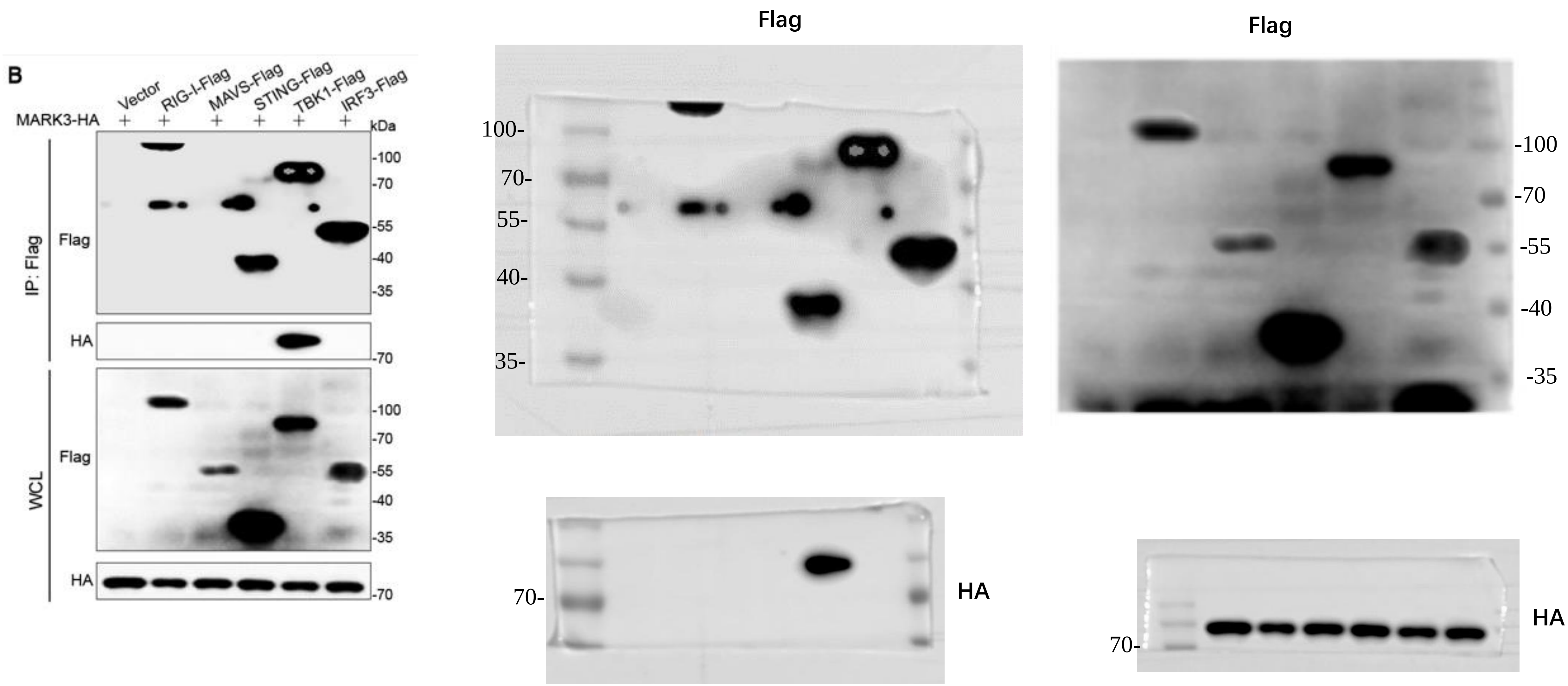
B
Vector
RIG-I-Flag
MAVS-Flag
STING-Flag
TBK1-Flag
IRF3-Flag
MARK3-HA
+ + + + + +
kDa
IP: Flag
Flag
HA
WCL
Flag
HA
-100
-70
-55
-40
-35
Flag
100-
70-
55-
40-
35-
70-
HA
Flag
-100
-70
-55
-40
-35
70-
HA

Fig. 7C

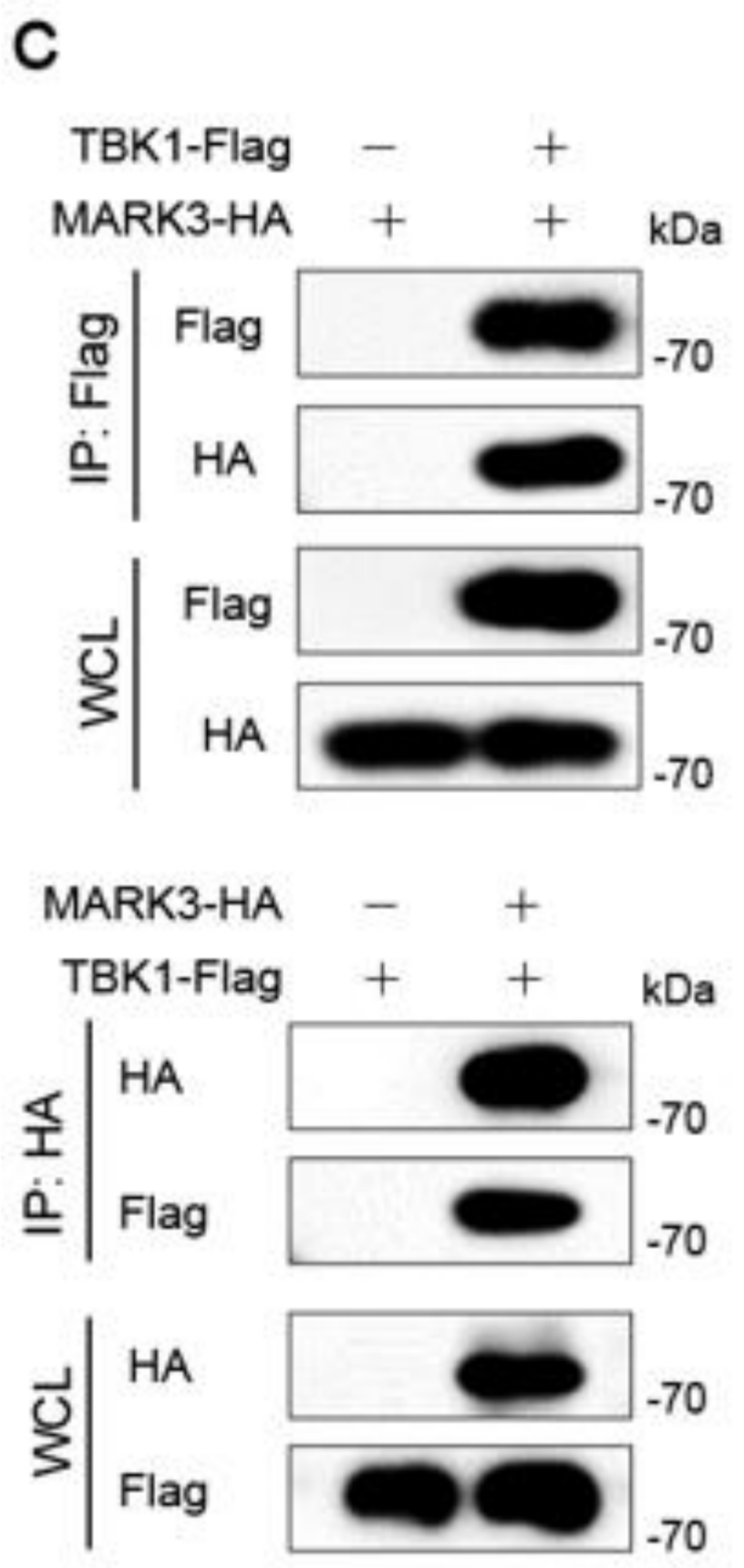
C
TBK1-Flag
MARK3-HA
kDa
IP: Flag
Flag
HA
WCL
Flag
HA
-70
MARK3-HA
TBK1-Flag
kDa
IP: HA
HA
Flag
WCL
HA
Flag
-70

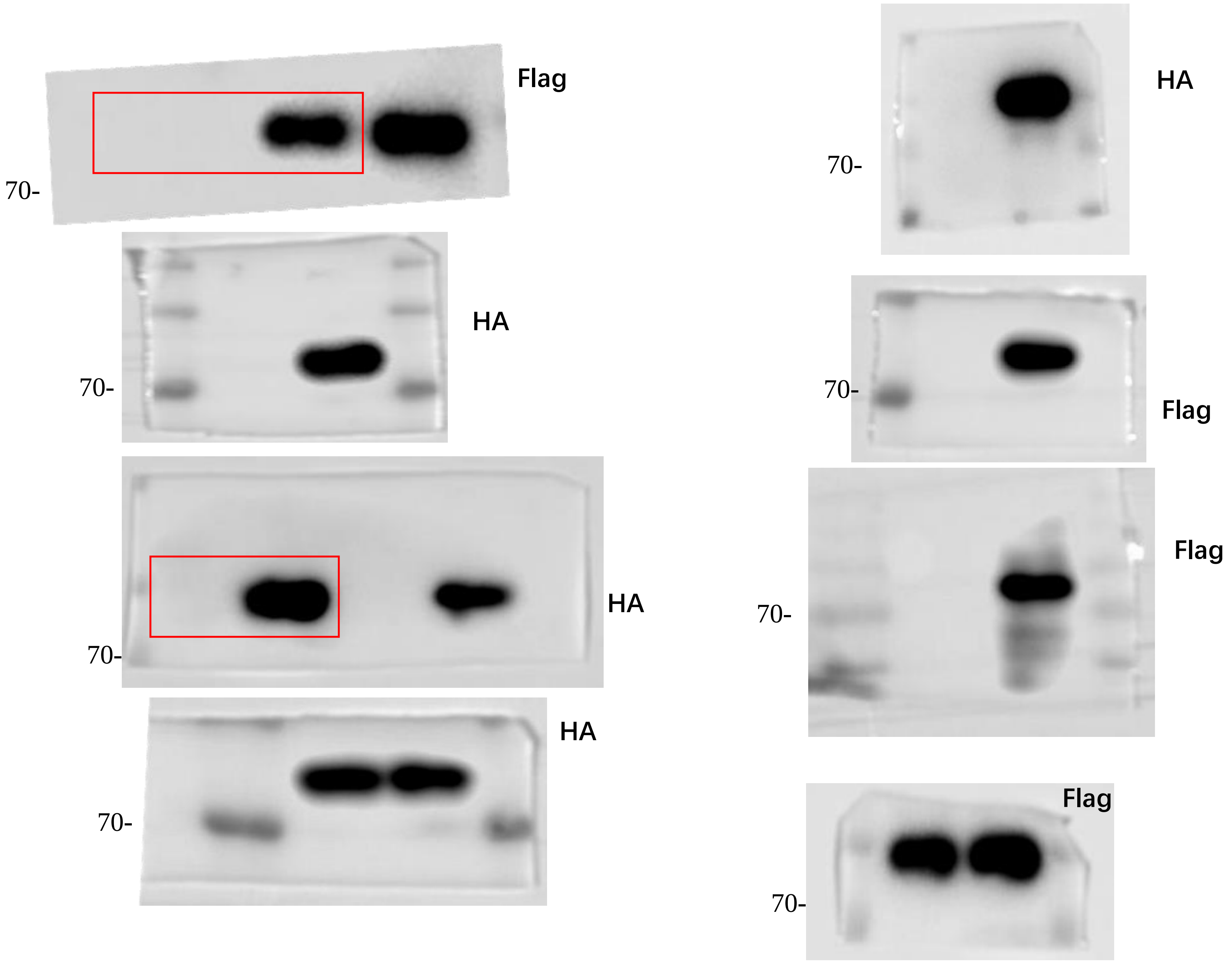
Flag
70-
HA
70-
HA
70-
HA
70-
HA
70-
Flag
70-
Flag
70-
Flag
70-

Fig. 7D

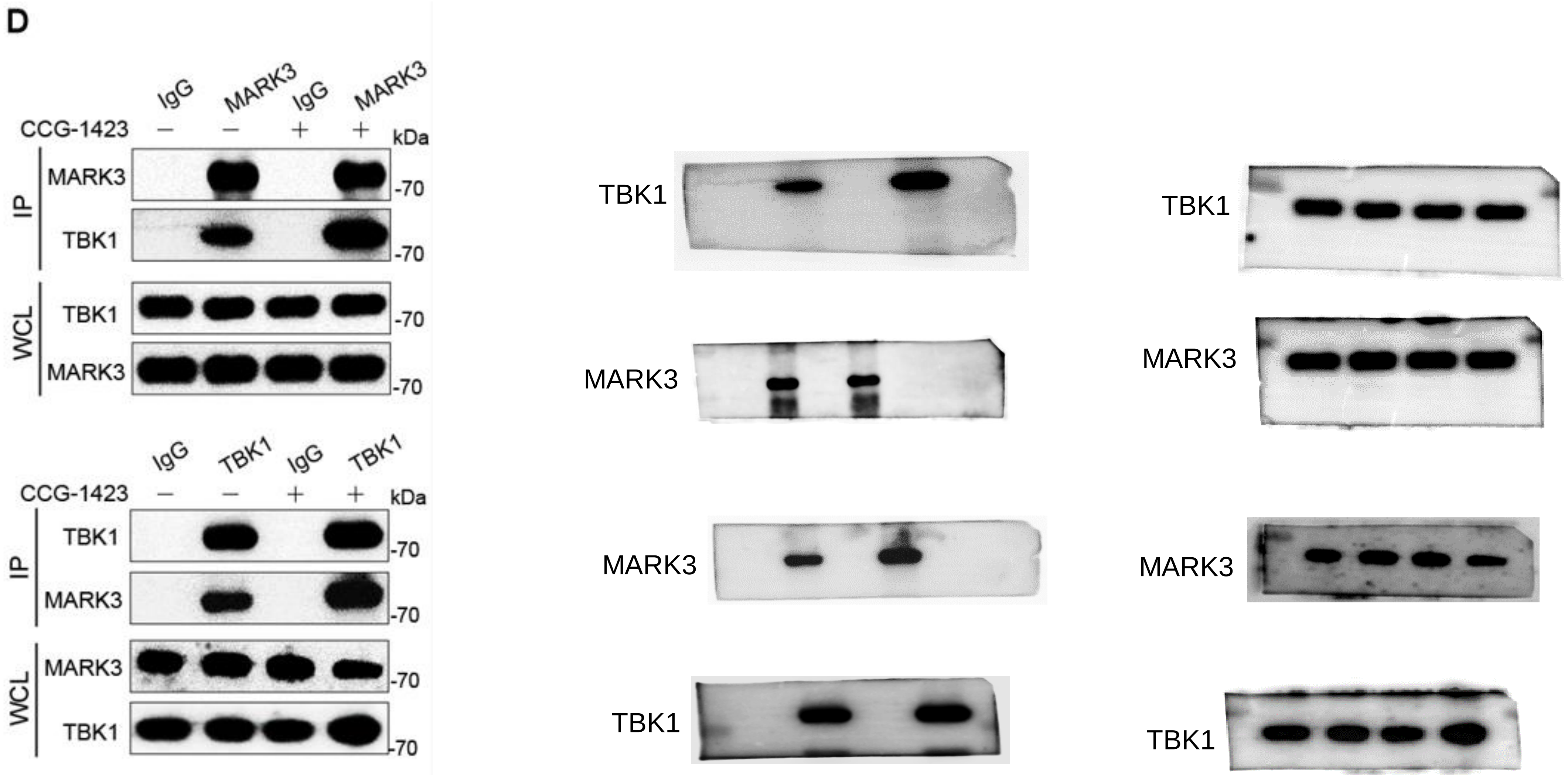
D
IgG
MARK3
IgG
MARK3
CCG-1423
– – + +
kDa
IP
MARK3
TBK1
WCL
TBK1
MARK3
-70
-70
-70
-70
IgG
TBK1
IgG
TBK1
CCG-1423
– – + +
kDa
IP
TBK1
MARK3
WCL
MARK3
TBK1
-70
-70
-70
-70
TBK1
MARK3
MARK3
TBK1
TBK1
MARK3
MARK3
TBK1

Fig. 7E

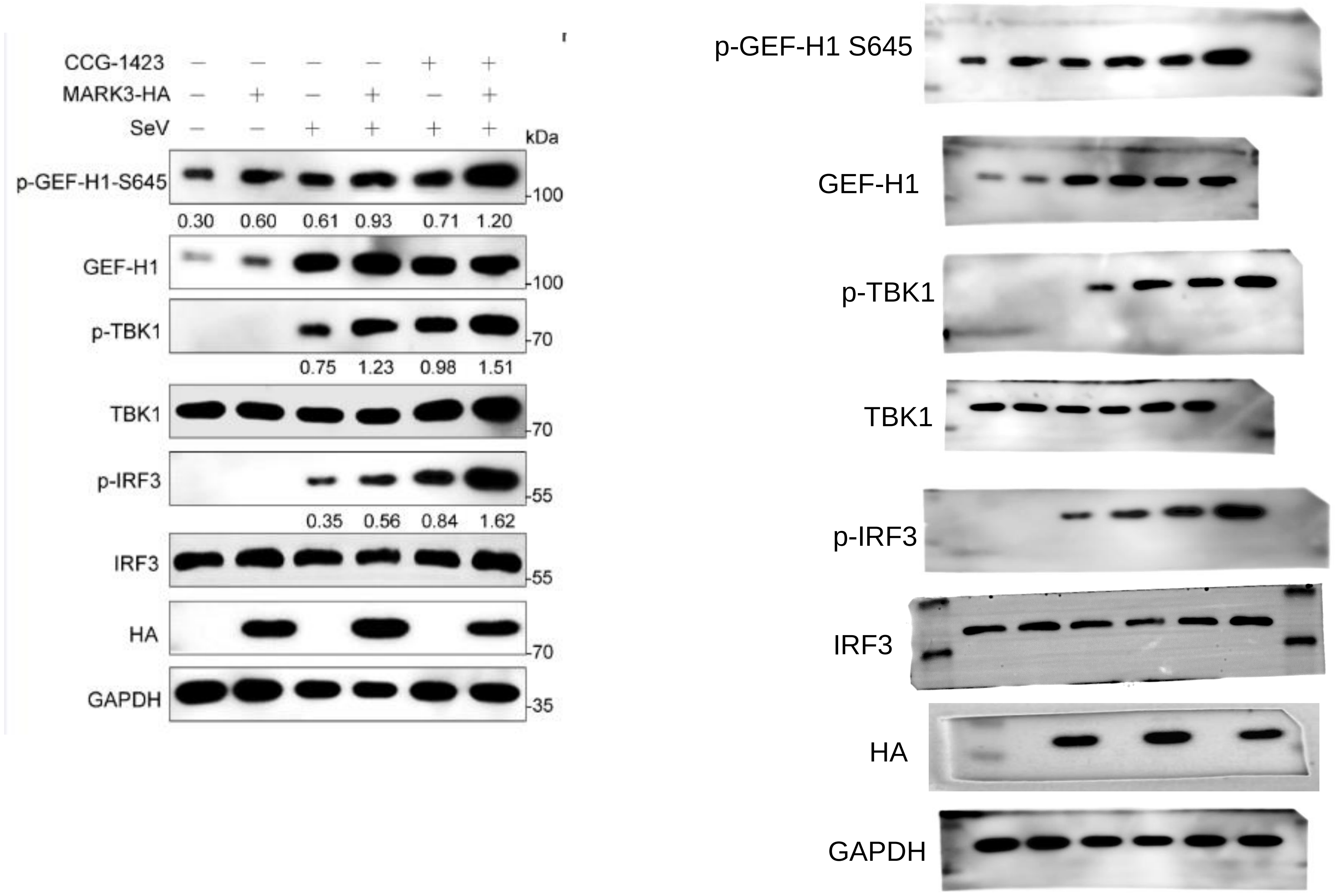

CCG-1423 − − − − + +
MARK3-HA − + − + − +
SeV − − + + + +
kDa
p-GEF-H1-S645
100
0.30 0.60 0.61 0.93 0.71 1.20
GEF-H1
100
p-TBK1
70
0.75 1.23 0.98 1.51
TBK1
70
p-IRF3
55
0.35 0.56 0.84 1.62
IRF3
55
HA
70
GAPDH
35
p-GEF-H1 S645
GEF-H1
p-TBK1
TBK1
p-IRF3
IRF3
HA
GAPDH

Fig. 7F

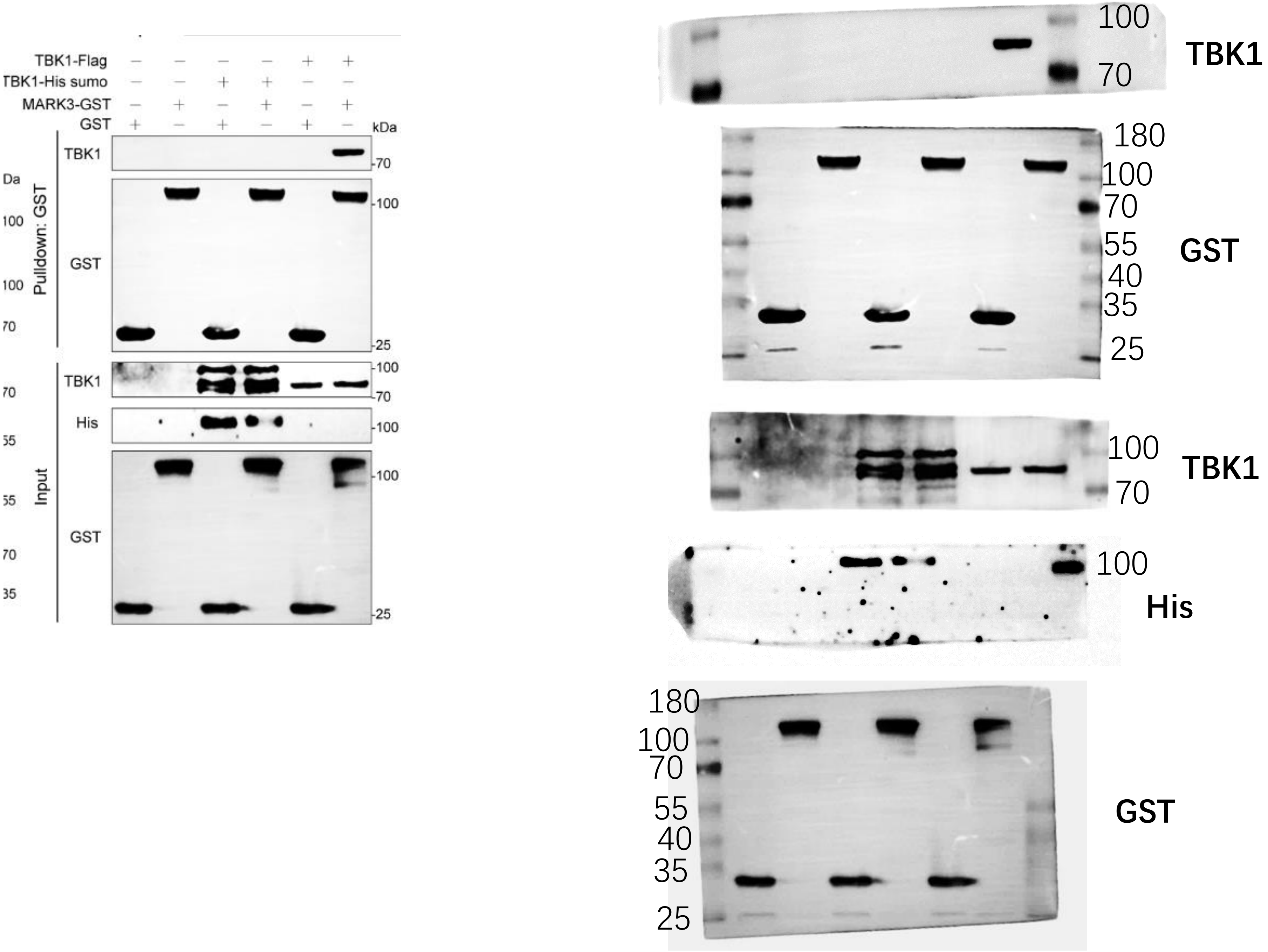

TBK1-Flag
TBK1-His sumo
MARK3-GST
GST
kDa
Pulldown: GST
TBK1
GST
Input
TBK1
His
GST
70
100
25
180
55
40
35
TBK1
GST
TBK1
His
GST

Fig. 7G

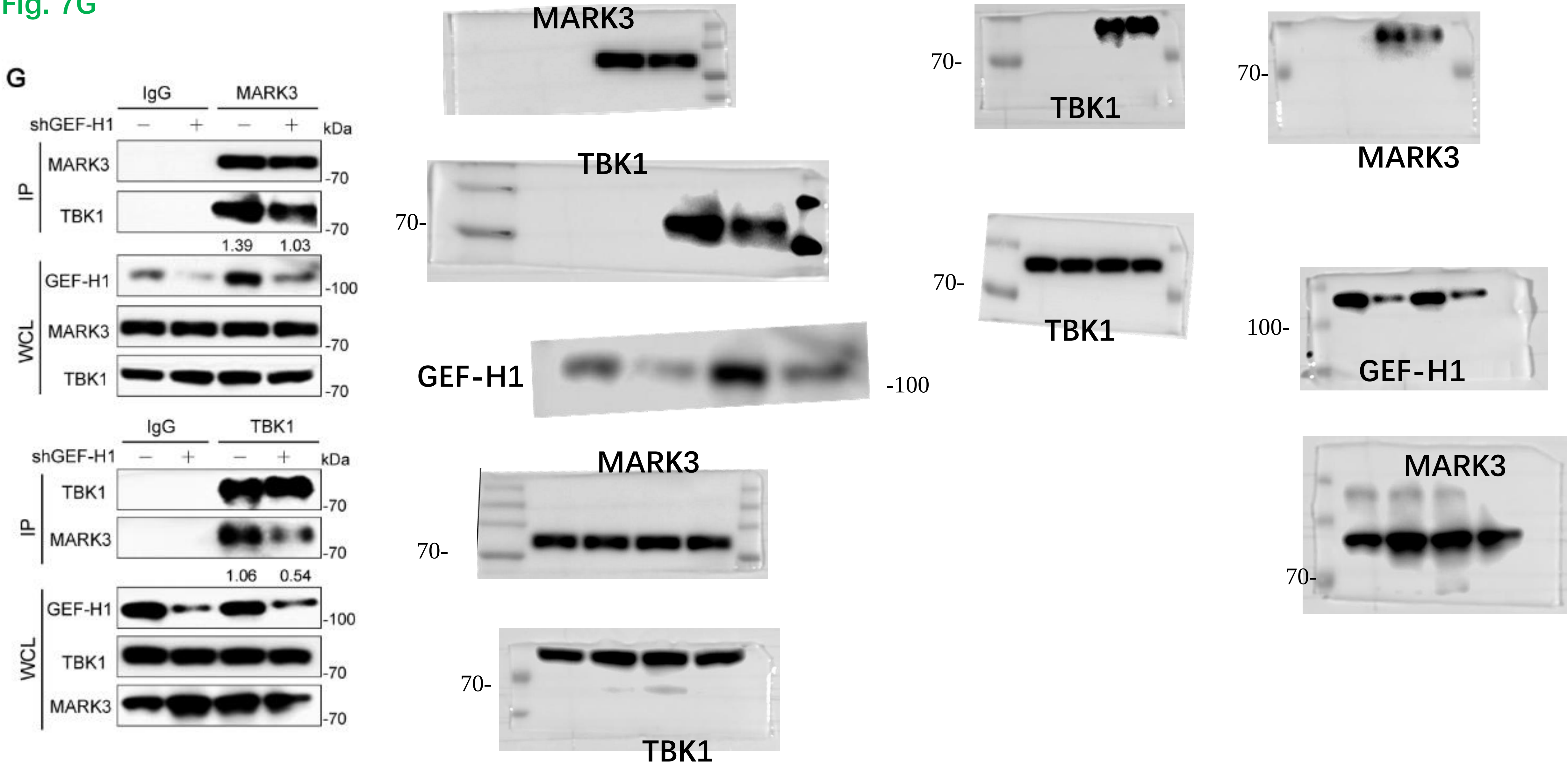
G
IgG
MARK3
shGEF-H1
kDa
IP
MARK3
TBK1
-70
-70
1.39
1.03
WCL
GEF-H1
MARK3
TBK1
-100
-70
-70
IgG
TBK1
shGEF-H1
kDa
IP
TBK1
MARK3
-70
-70
1.06
0.54
WCL
GEF-H1
TBK1
MARK3
-100
-70
-70
MARK3
TBK1
70-
GEF-H1
-100
MARK3
70-
70-
TBK1
TBK1
70-
TBK1
70-
70-
MARK3
100-
GEF-H1
MARK3
70-

Fig. 7H

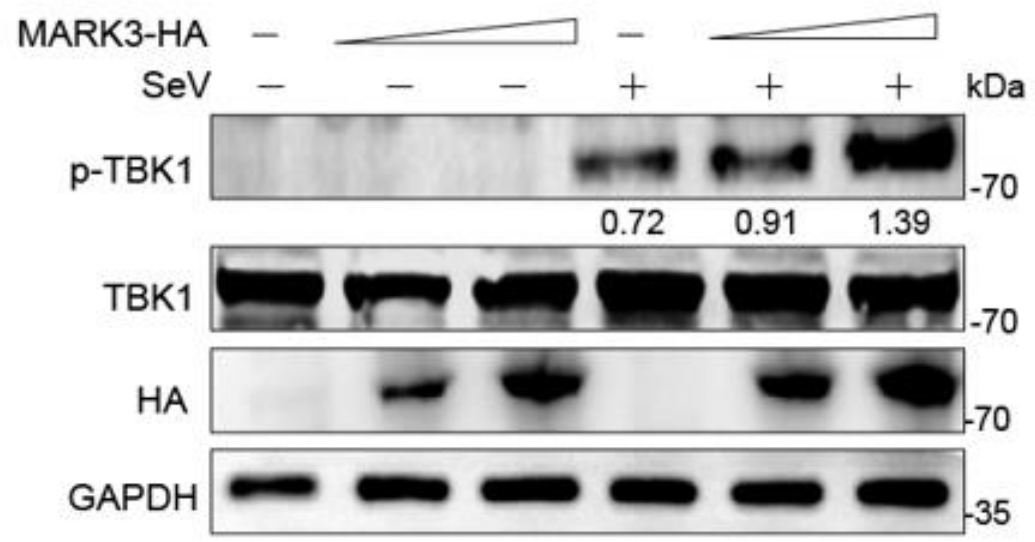
MARK3-HA
SeV
kDa
p-TBK1
TBK1
HA
GAPDH
0.72
0.91
1.39
-70
-70
-70
-35

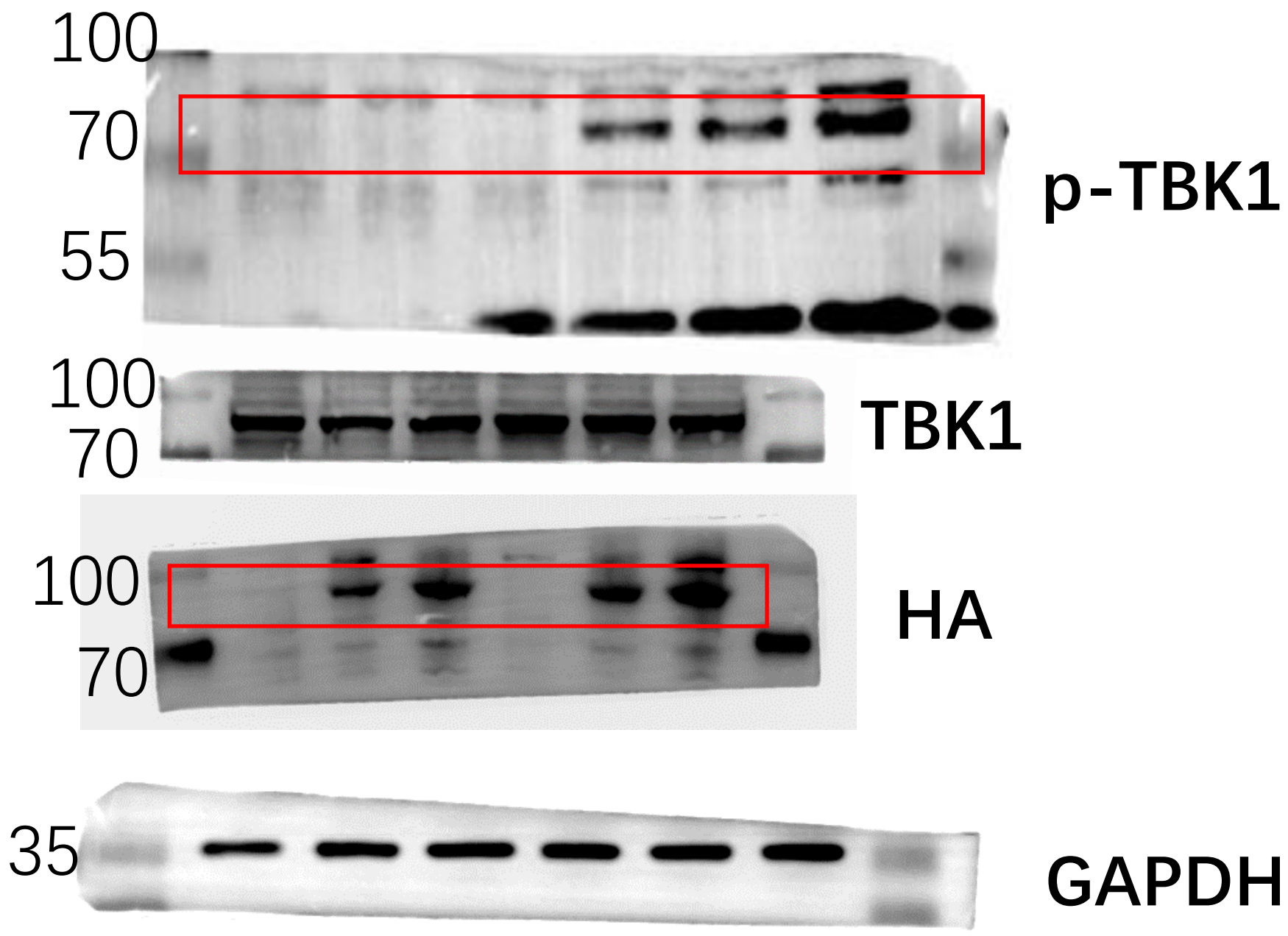
100
70
55
p-TBK1
100
70
TBK1
100
70
HA
35
GAPDH

Fig. 7J

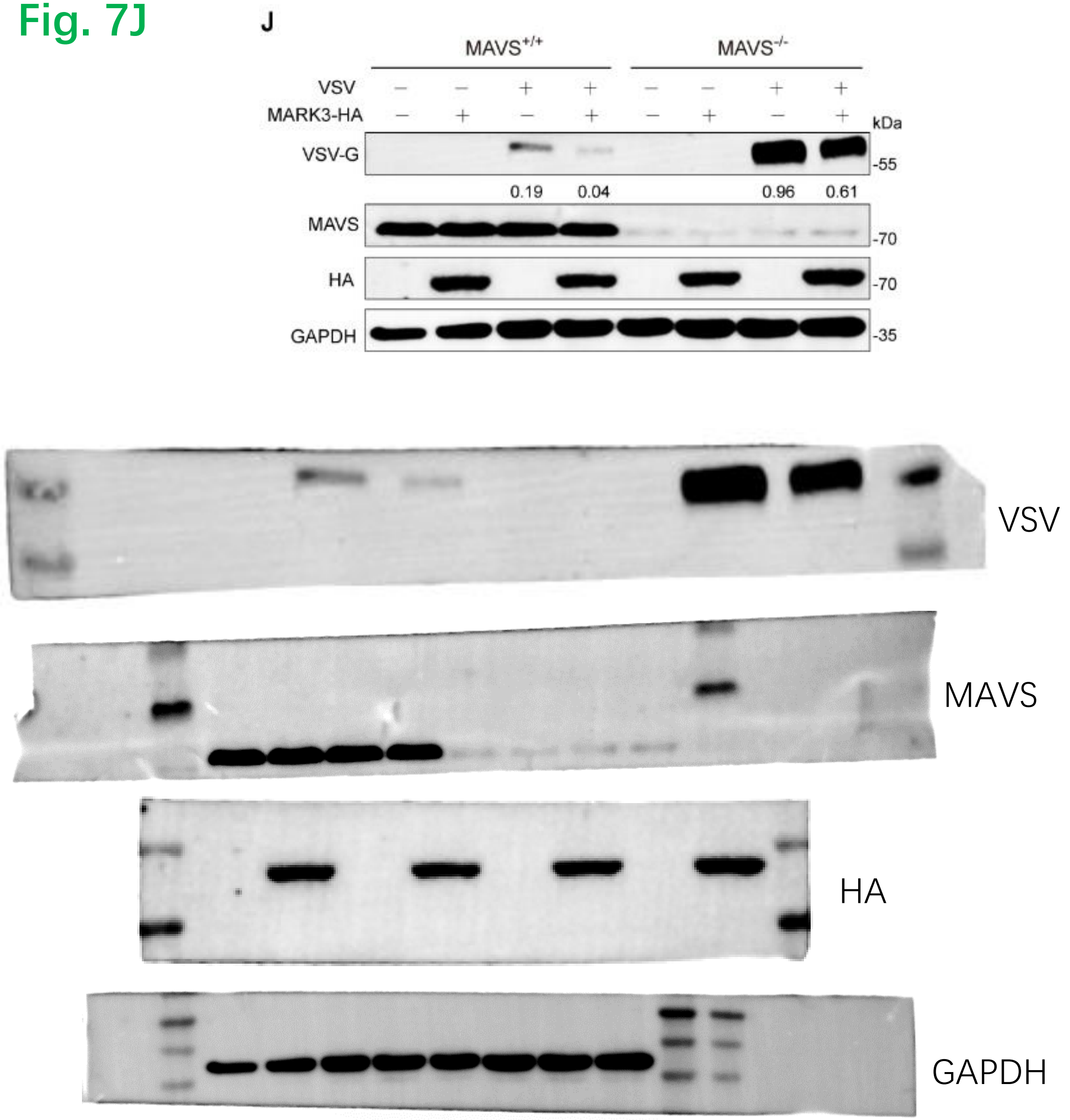
J
MAVS+/+
MAVS-/-
VSV
MARK3-HA
kDa
VSV-G
0.19 0.04 0.96 0.61
MAVS
HA
GAPDH
-55
-70
-70
-35
VSV
MAVS
HA
GAPDH

Fig. 7L

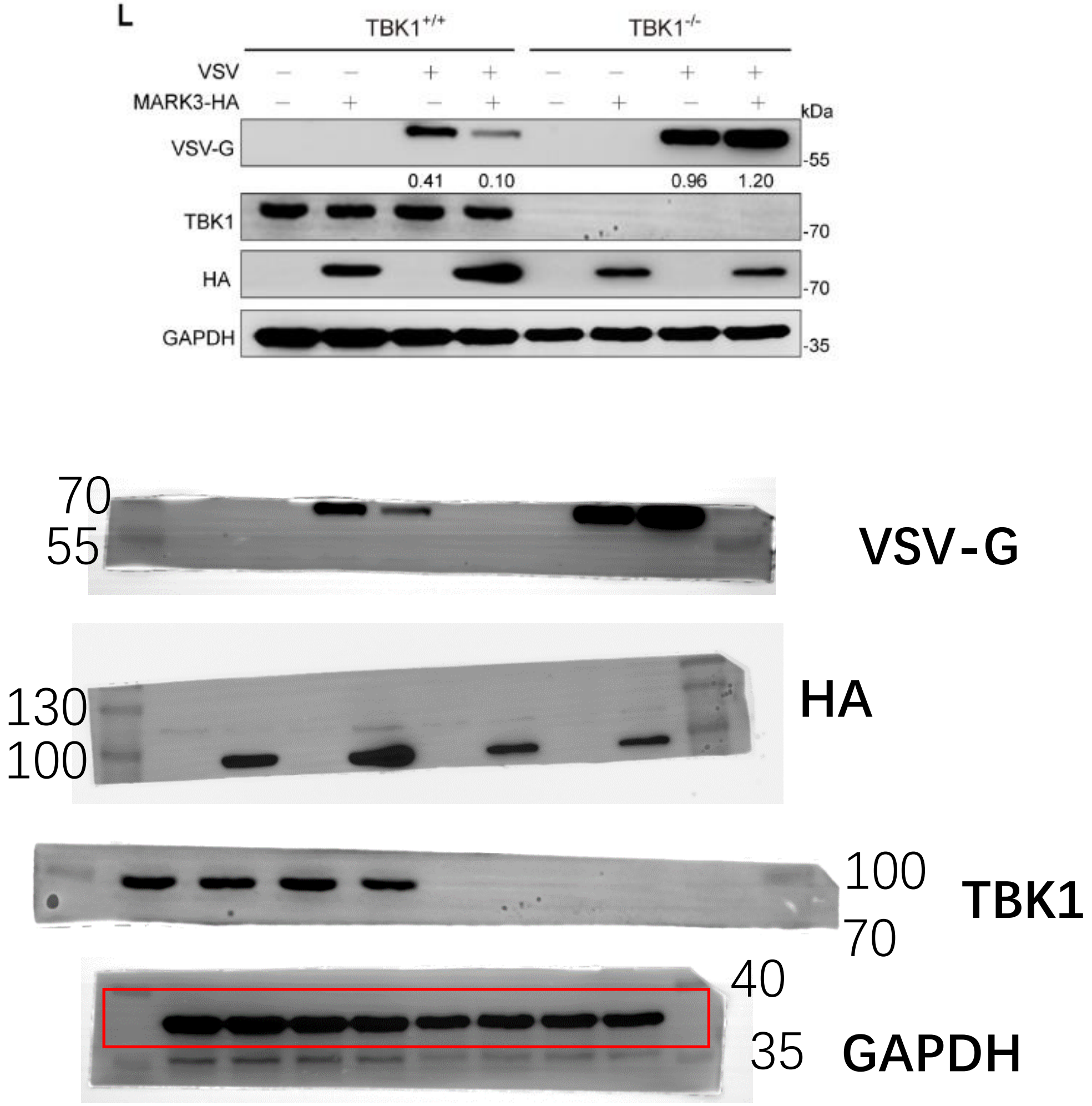
L
TBK1+/+
TBK1-/-
VSV
MARK3-HA
kDa
VSV-G
0.41 0.10 0.96 1.20
TBK1
HA
GAPDH
-55
-70
-70
-35
70
55
VSV-G
130
100
HA
100
70
TBK1
40
35
GAPDH

Supplementary Figures

Fig. S2A

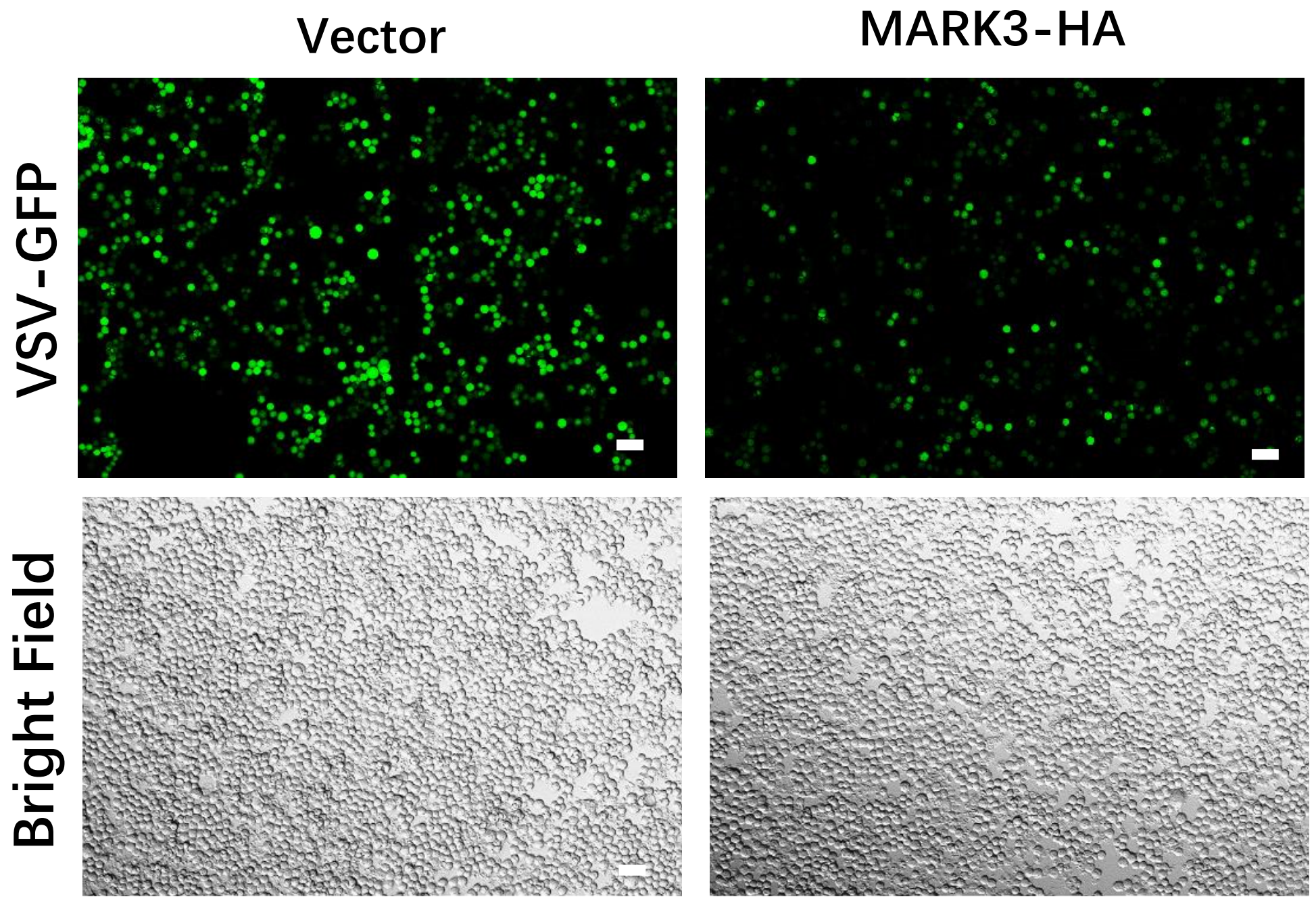

Fig. S2B

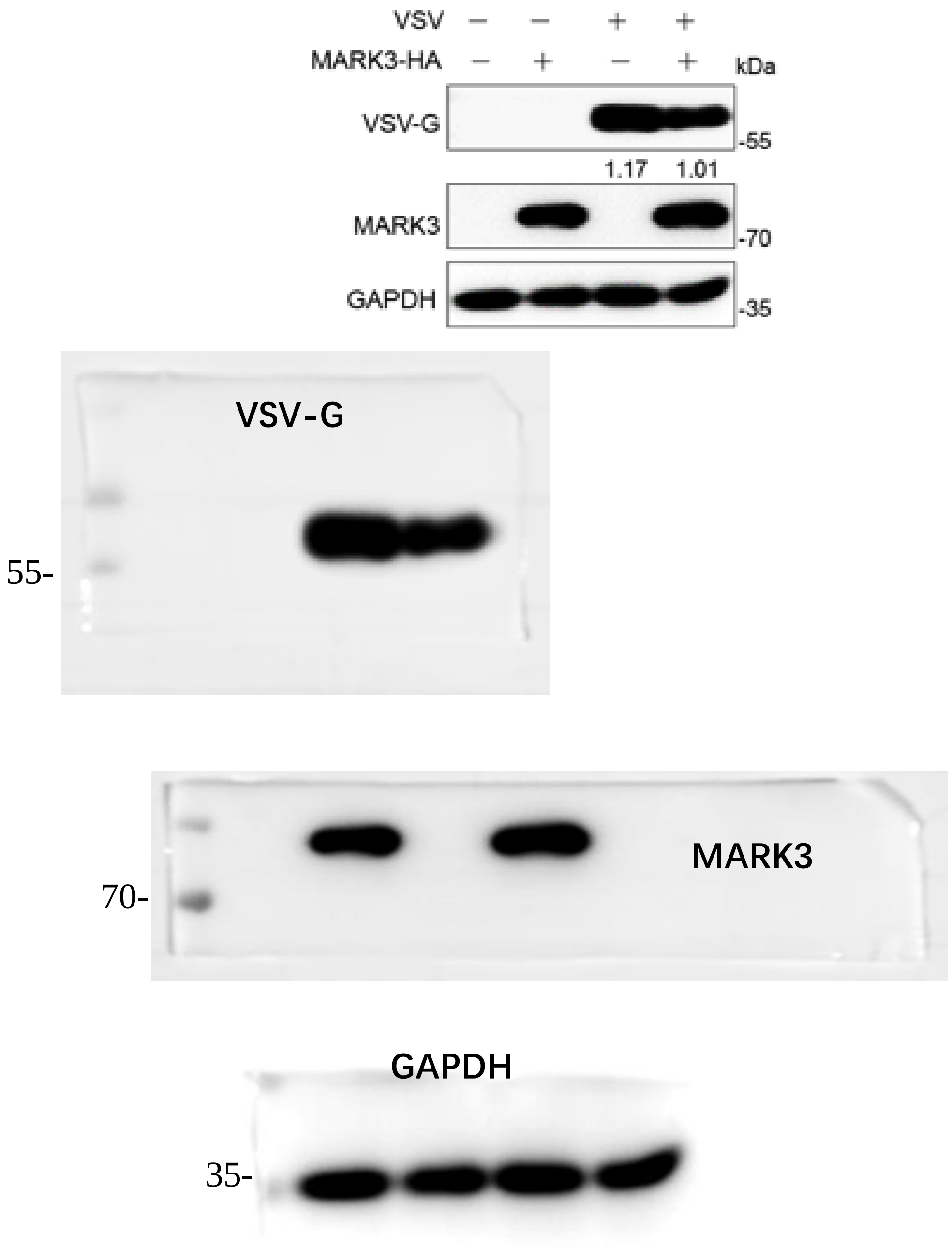
VSV – – + +
MARK3-HA – + – +
kDa
VSV-G
-55
1.17 1.01
MARK3
-70
GAPDH
-35
VSV-G
55-
70-
MARK3
GAPDH
35-

Fig. S2C

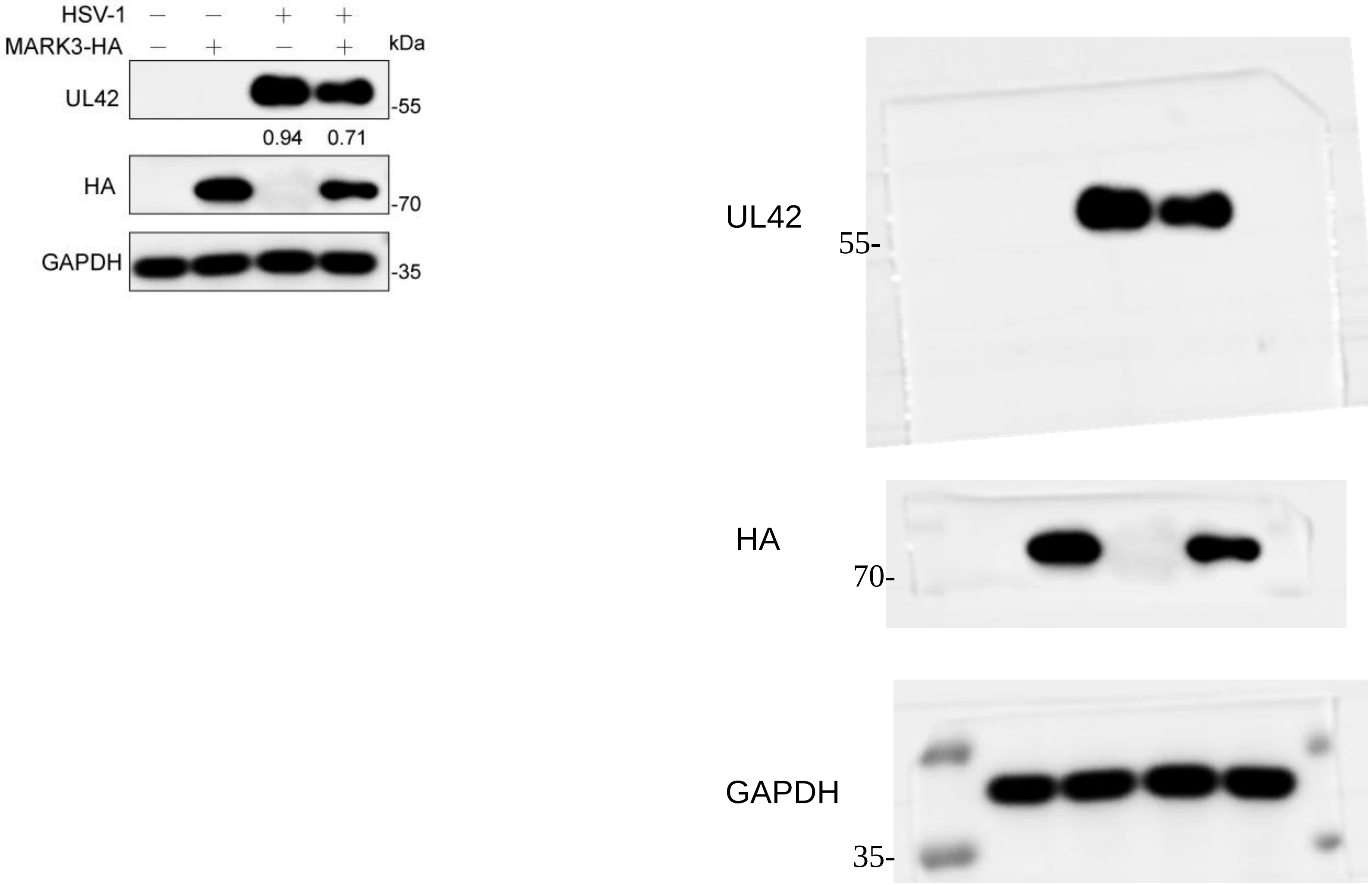
HSV-1 − − + +
MARK3-HA − + − +
kDa
UL42
-55
0.94 0.71
HA
-70
GAPDH
-35
UL42
55-
HA
70-
GAPDH
35-

Fig. S2D

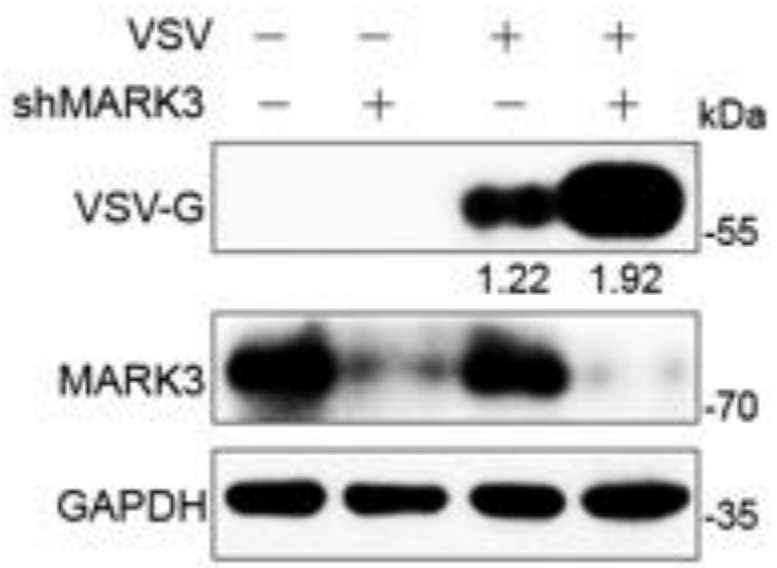
VSV − − + +
shMARK3 − + − + kDa
VSV-G
-55
1.22 1.92
MARK3
-70
GAPDH
-35

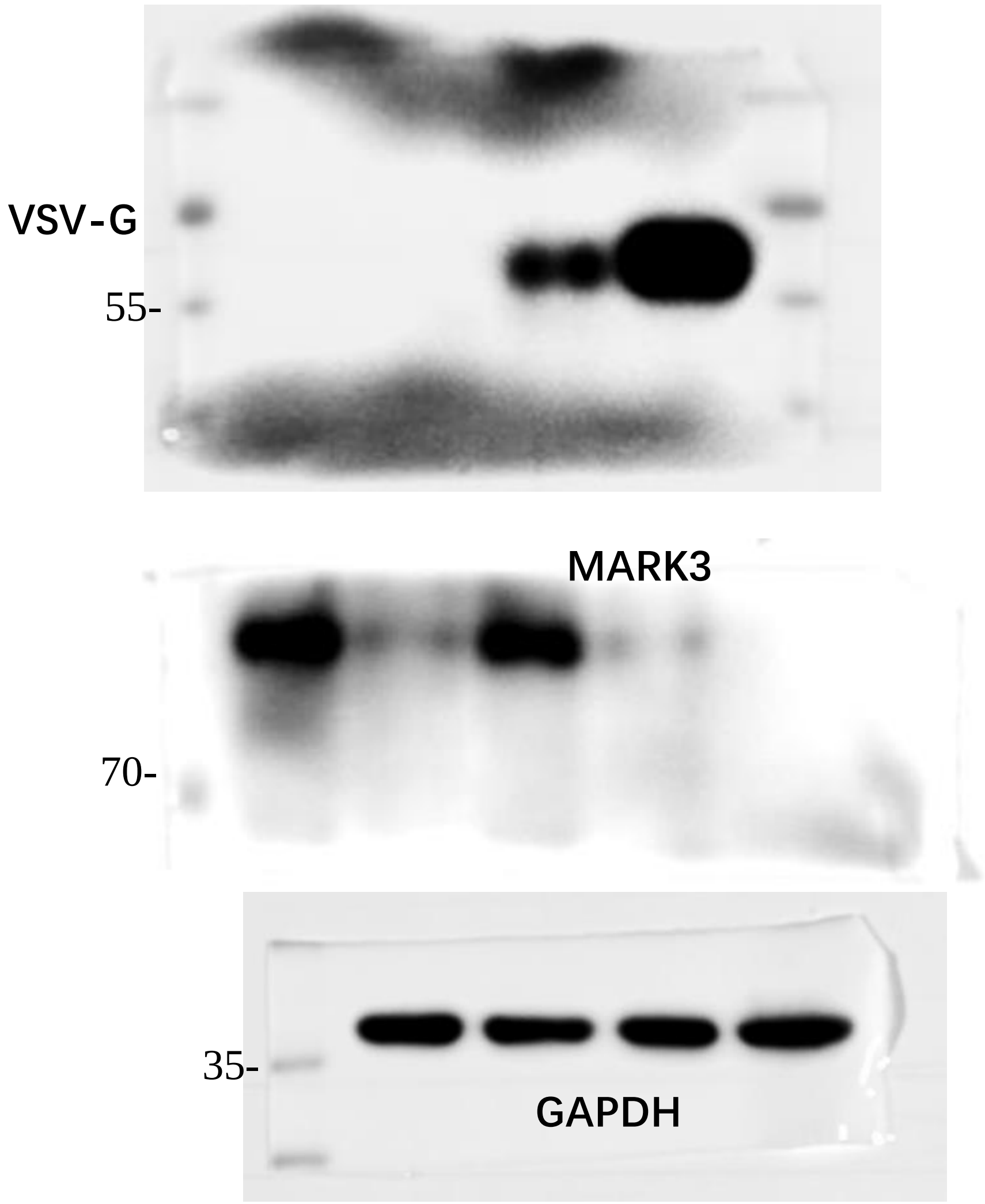
VSV-G
55-
MARK3
70-
35-
GAPDH

Fig. S2E

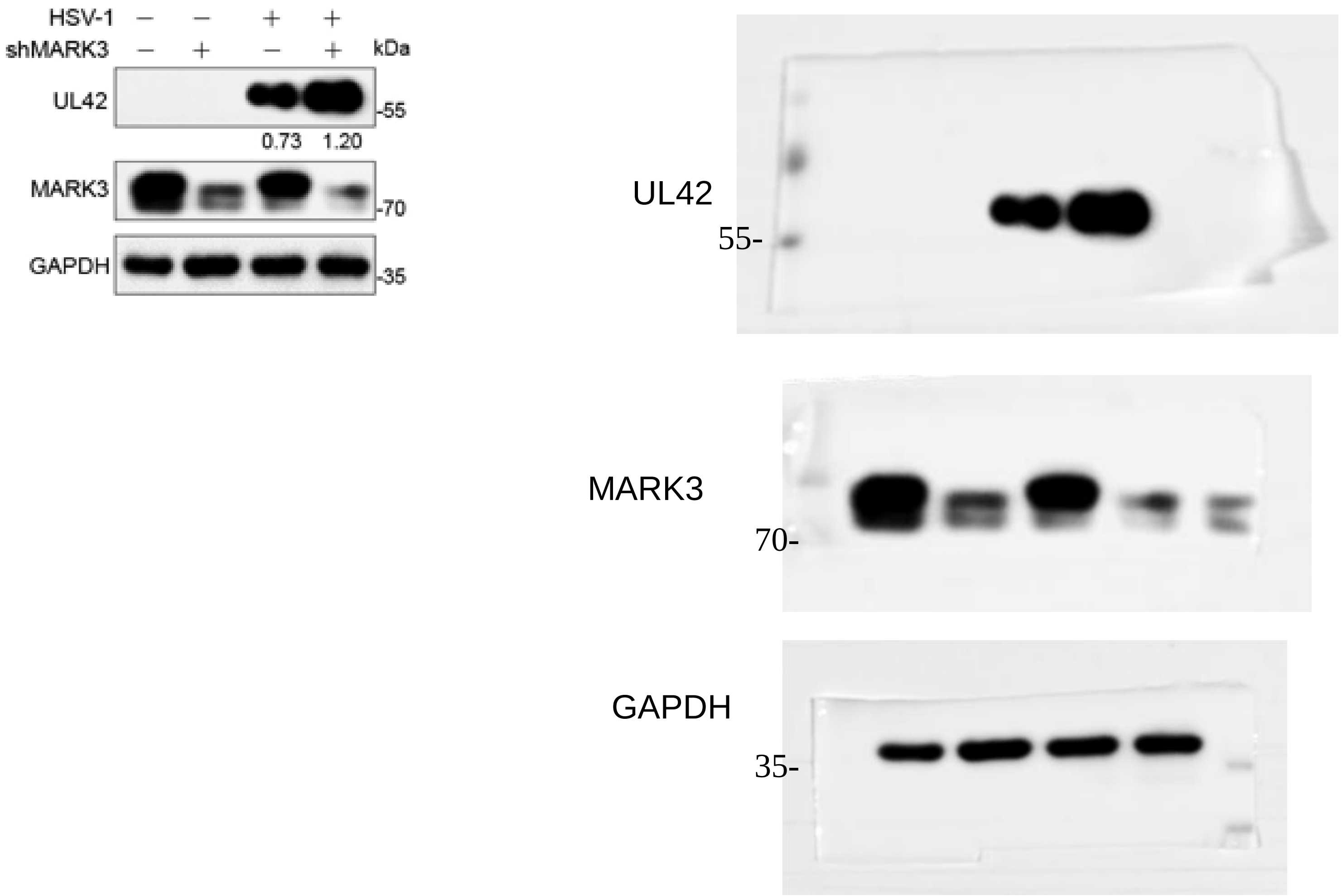
HSV-1 – – + +
shMARK3 – + – +
kDa
UL42
-55
0.73 1.20
MARK3
-70
GAPDH
-35
UL42
55-
MARK3
70-
GAPDH
35-

Fig. S5A/B

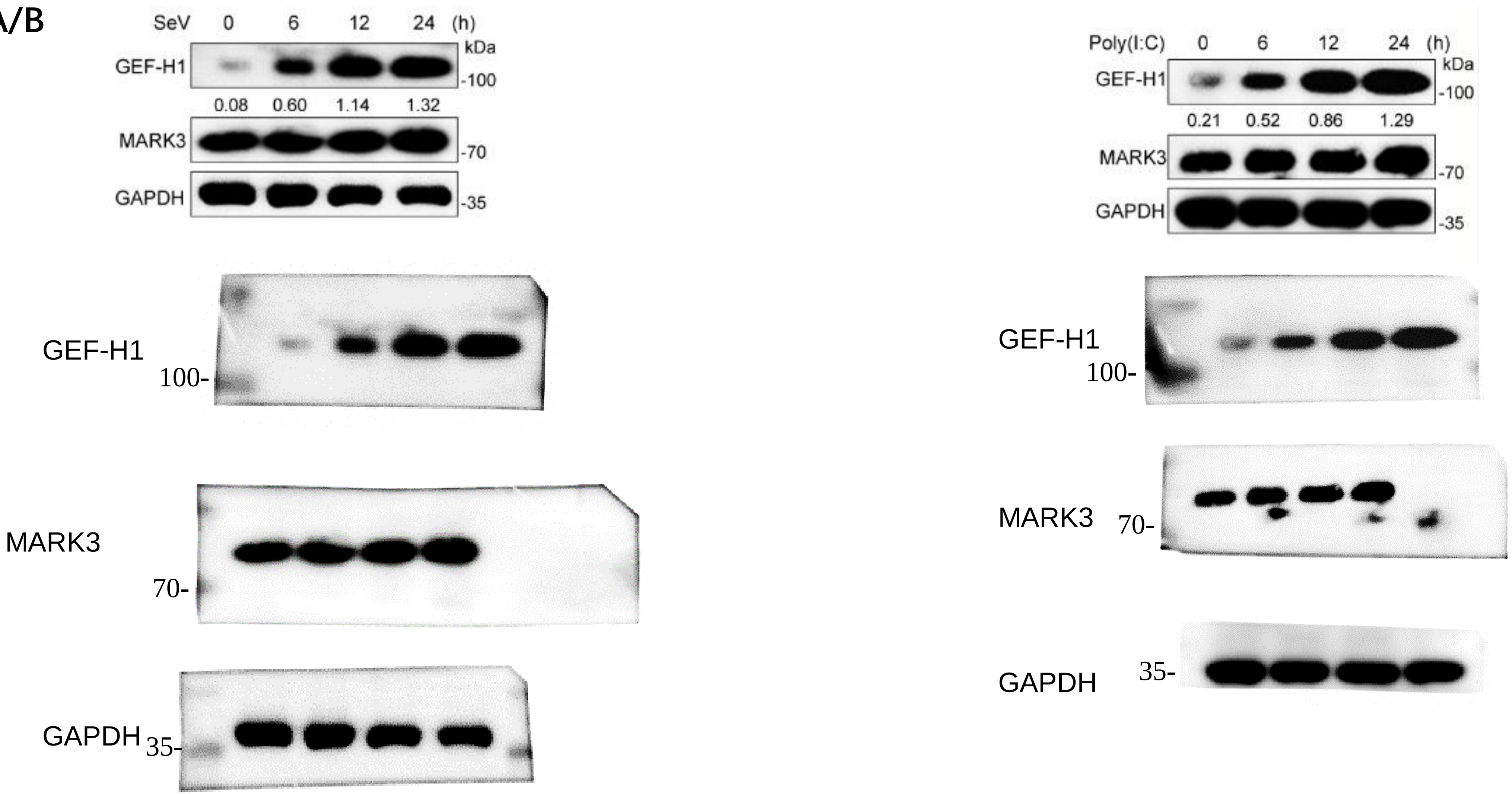
SeV 0 6 12 24 (h)
kDa
GEF-H1
-100
0.08 0.60 1.14 1.32
MARK3
-70
GAPDH
-35
Poly(I:C) 0 6 12 24 (h)
kDa
GEF-H1
-100
0.21 0.52 0.86 1.29
MARK3
-70
GAPDH
-35
GEF-H1
100-
MARK3
70-
GAPDH
35-
GEF-H1
100-
MARK3
70-
GAPDH
35-

Fig. S5C/D

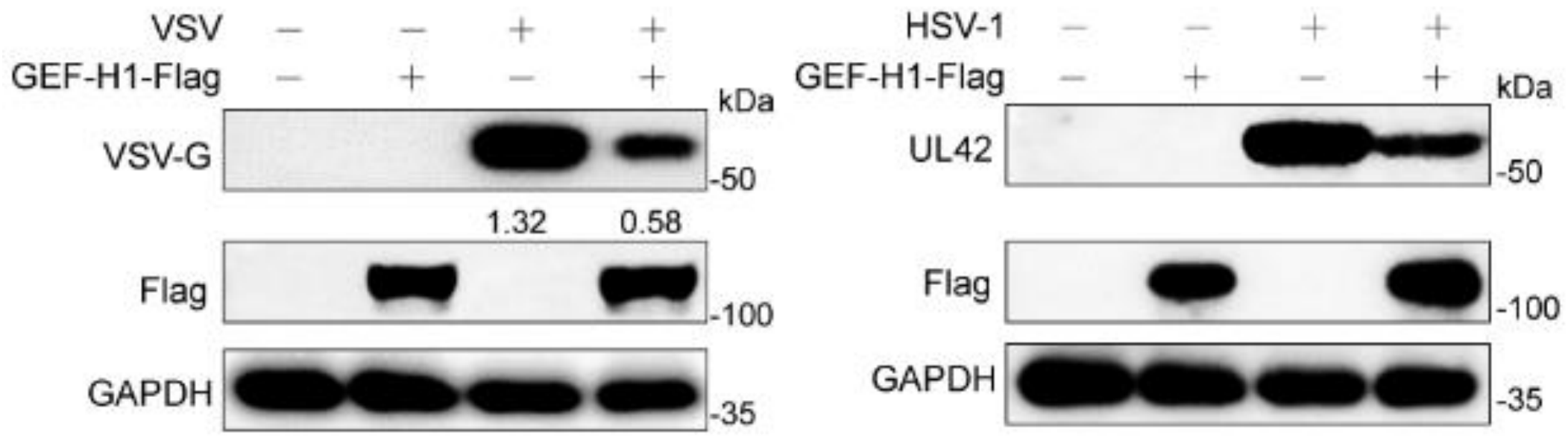
VSV
GEF-H1-Flag
kDa
VSV-G
-50
1.32
0.58
Flag
-100
GAPDH
-35
HSV-1
GEF-H1-Flag
kDa
UL42
-50
Flag
-100
GAPDH
-35

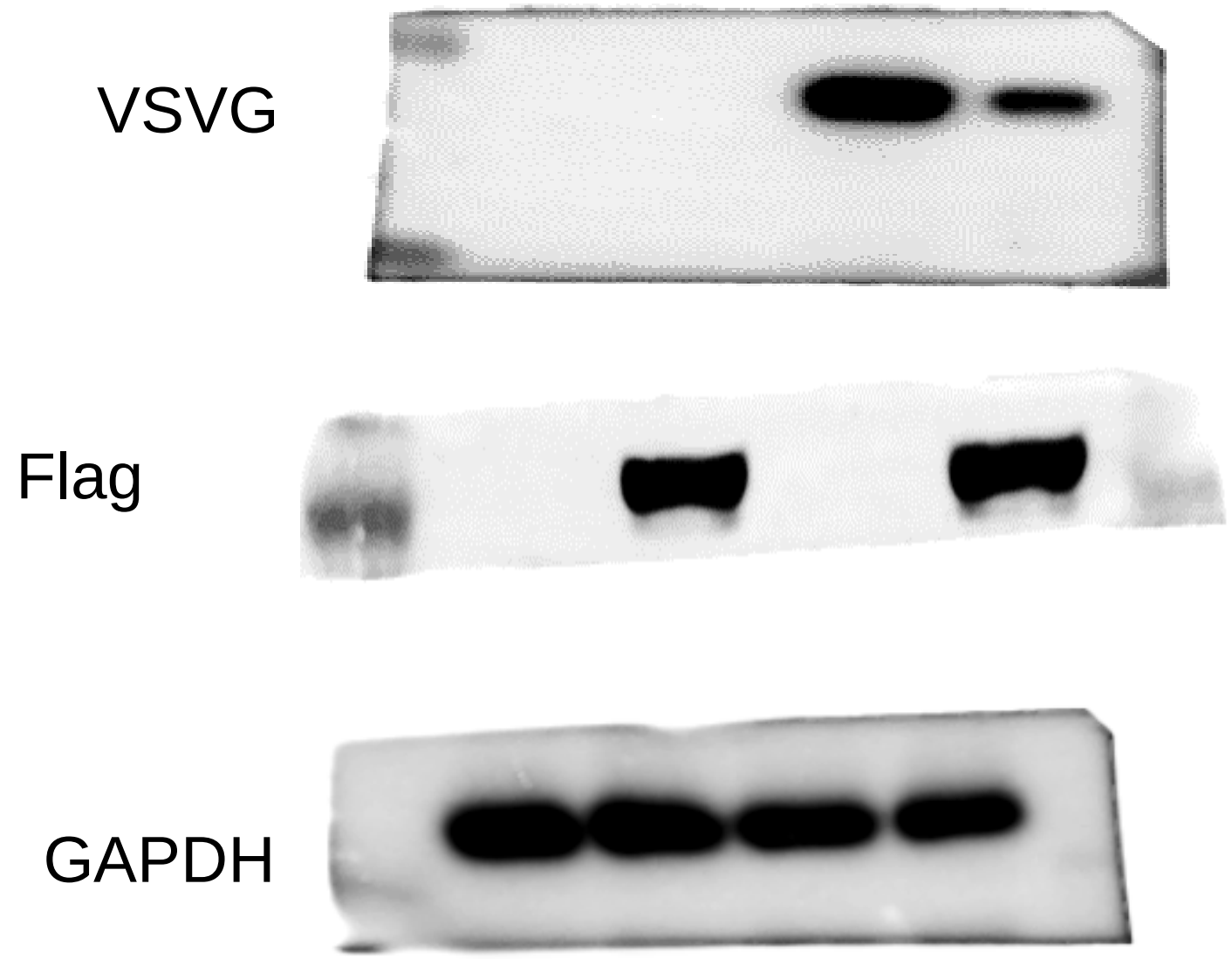
VSVG
Flag
GAPDH

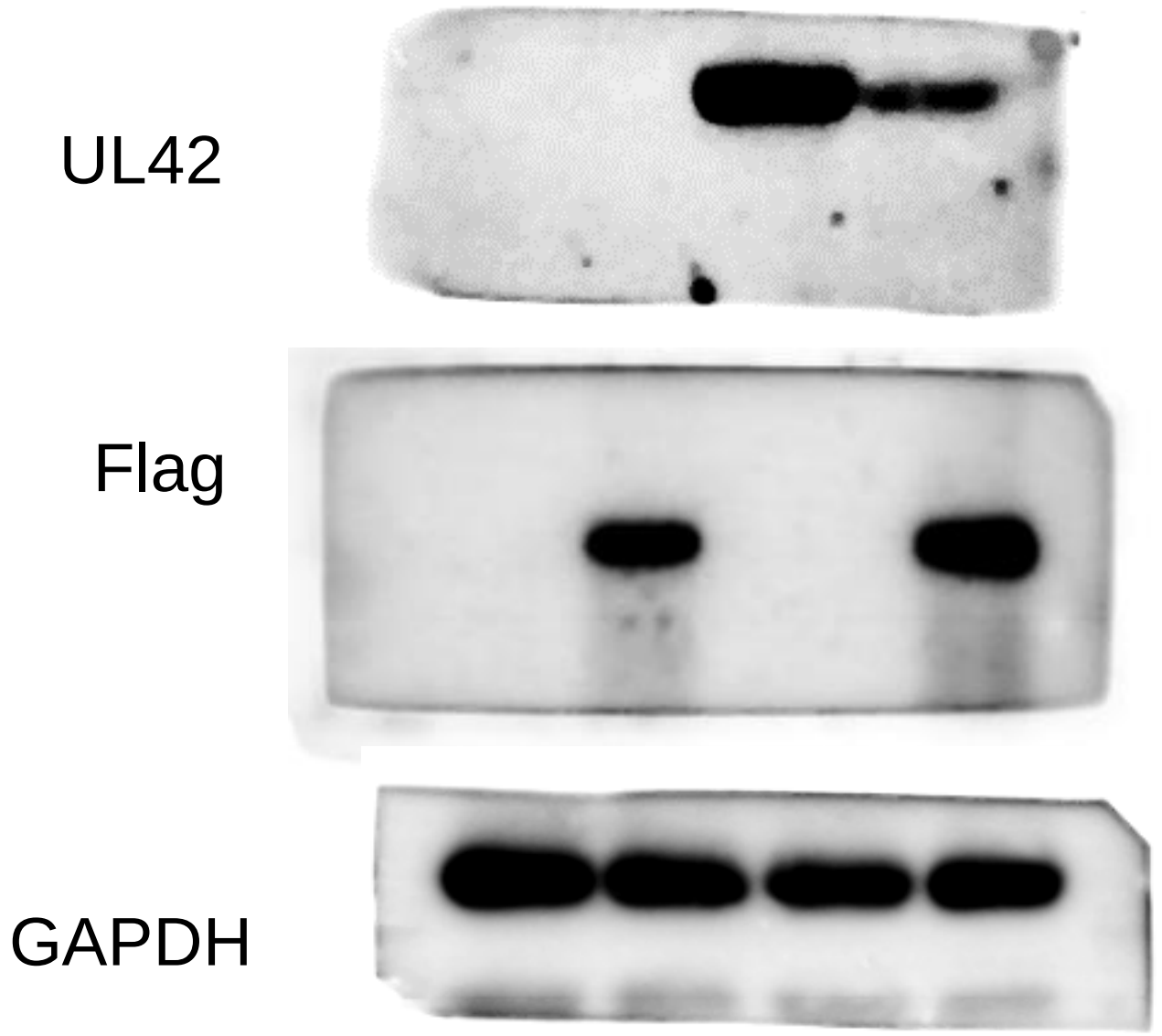
UL42
Flag
GAPDH

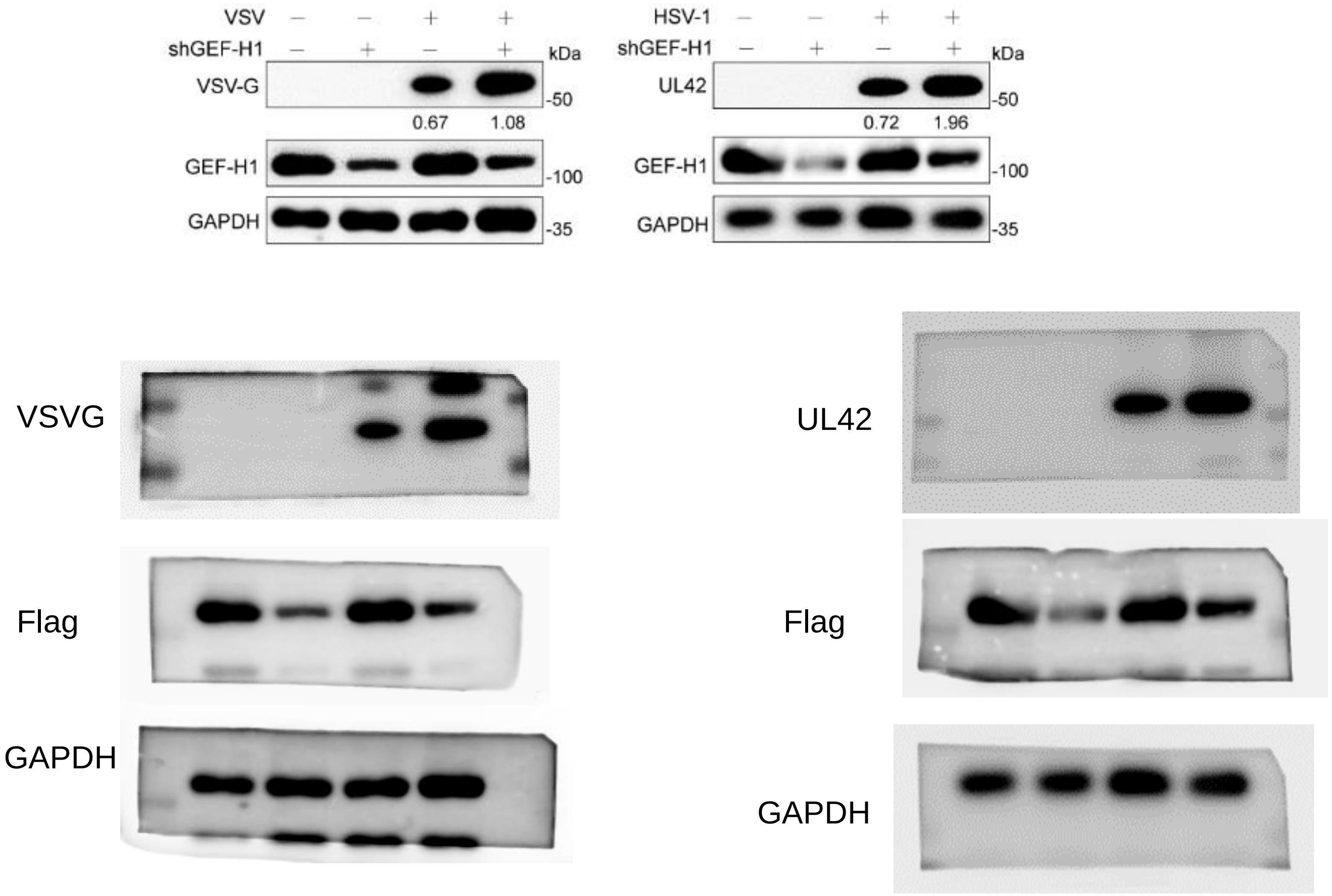

VSV − − + +
shGEF-H1 − + − +
kDa
VSV-G
-50
0.67
1.08
GEF-H1
-100
GAPDH
-35
HSV-1 − − + +
shGEF-H1 − + − +
kDa
UL42
-50
0.72
1.96
GEF-H1
-100
GAPDH
-35
VSVG
Flag
GAPDH
UL42
Flag
GAPDH

Fig. S5E/F

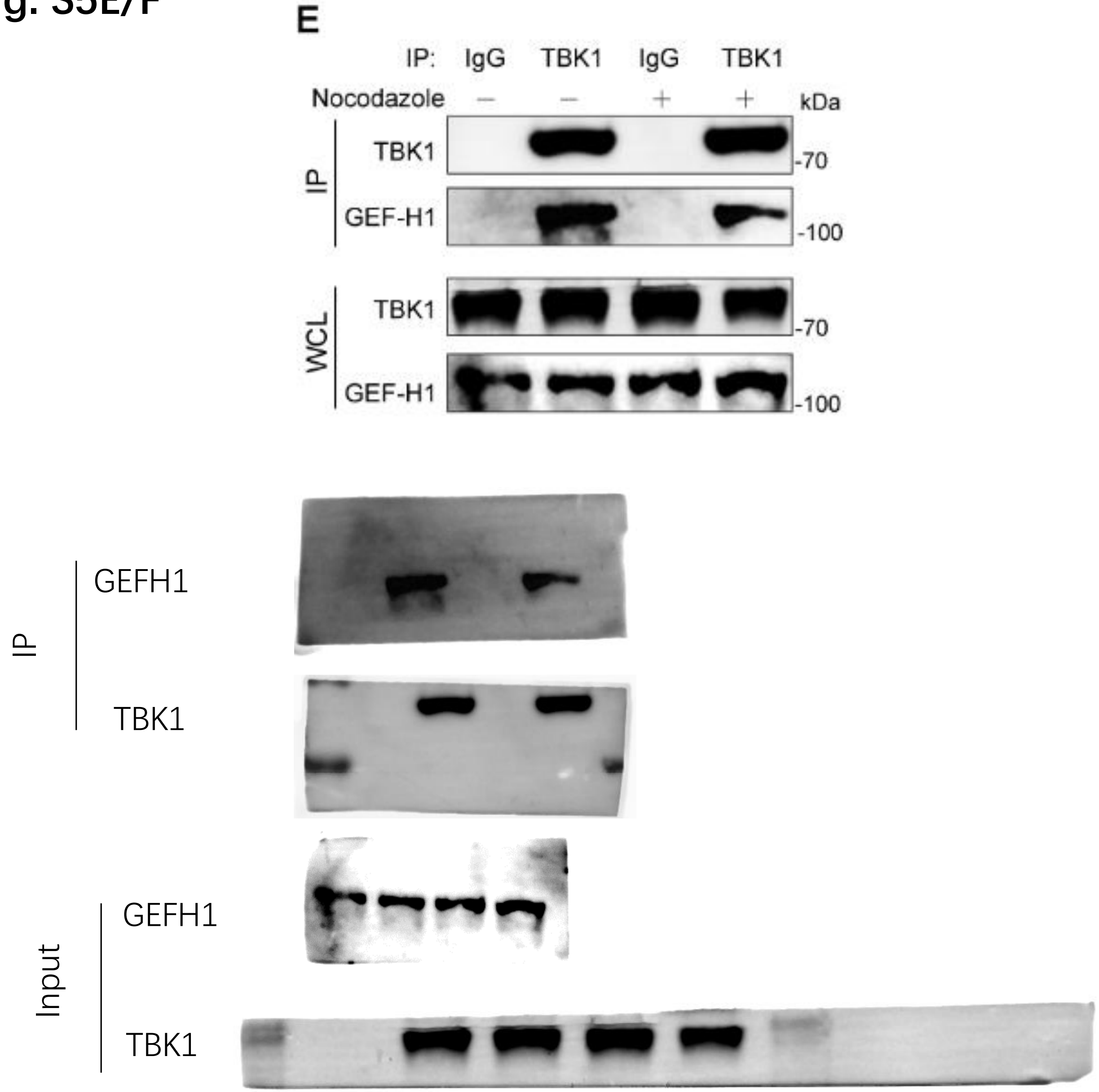
E
IP: IgG TBK1 IgG TBK1
Nocodazole − − + +
kDa
IP
TBK1
GEF-H1
WCL
TBK1
GEF-H1
-70
-100
-70
-100
IP
GEFH1
TBK1
Input
GEFH1
TBK1

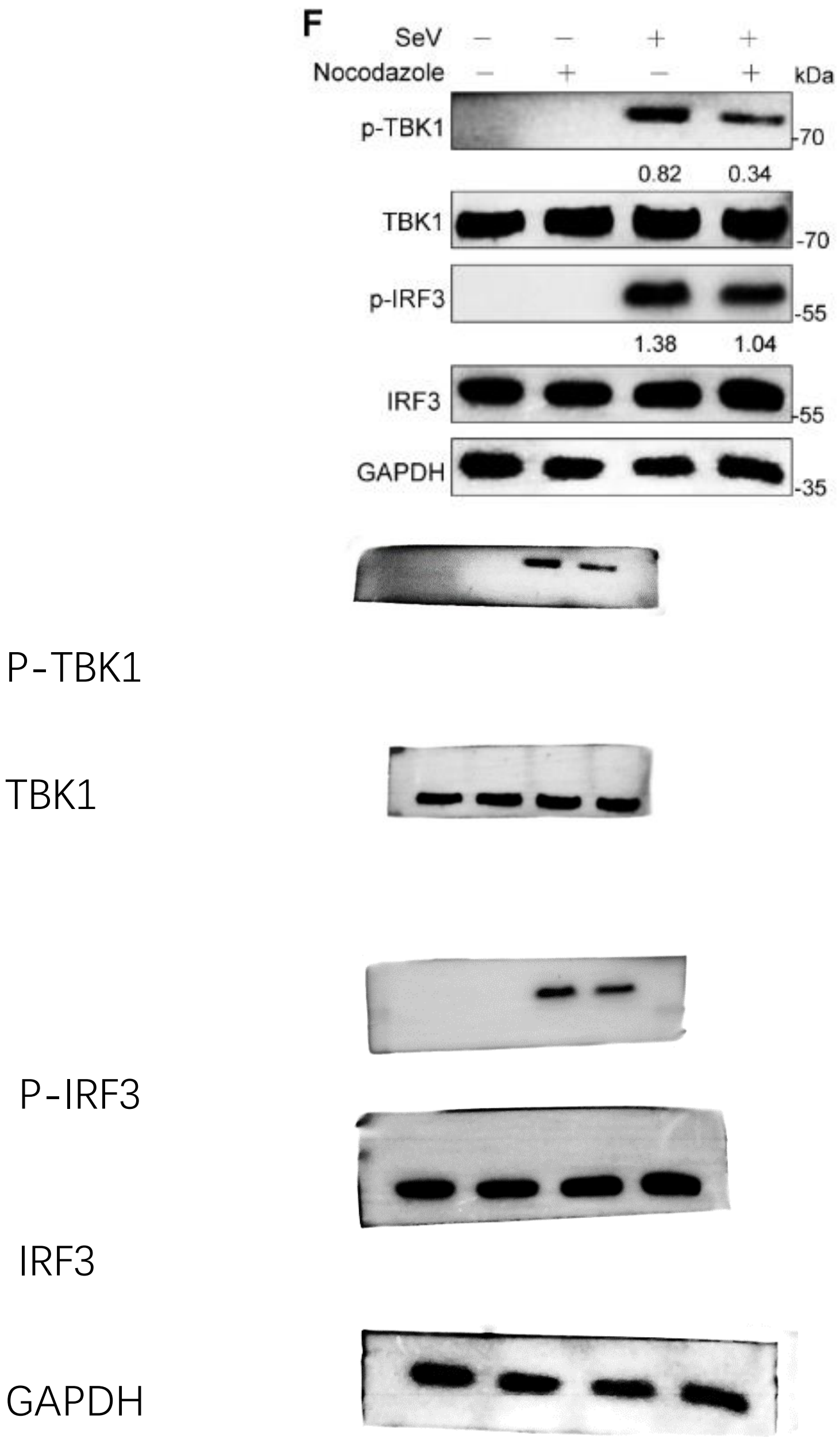
F
SeV − − + +
Nocodazole − + − +
kDa
p-TBK1
-70
0.82 0.34
TBK1
-70
p-IRF3
-55
1.38 1.04
IRF3
-55
GAPDH
-35
P-TBK1
TBK1
P-IRF3
IRF3
GAPDH

Fig. S6B

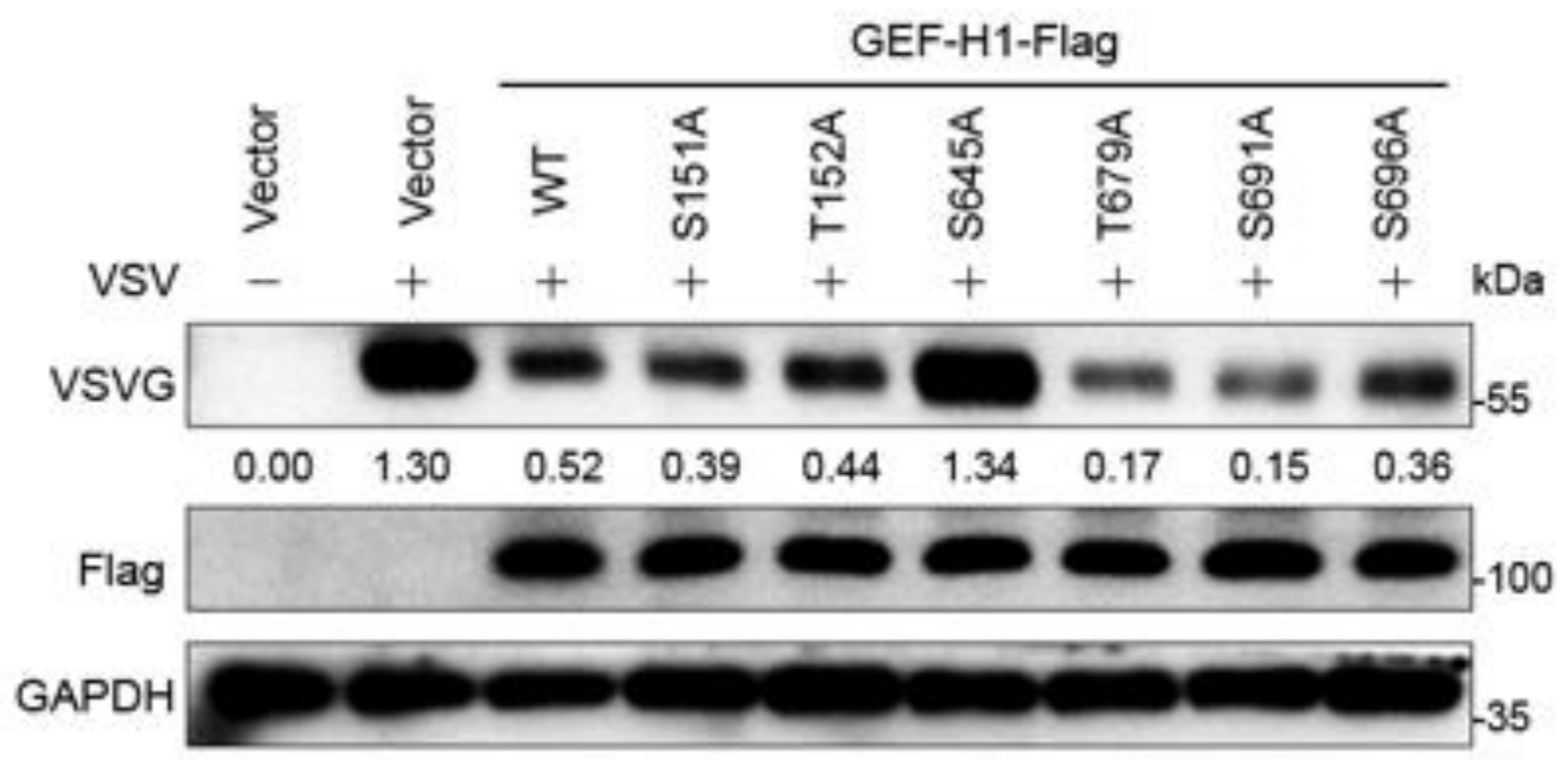
GEF-H1-Flag
Vector
Vector
WT
S151A
T152A
S645A
T679A
S691A
S696A
VSV
−
+
+
+
+
+
+
+
+
kDa
VSVG
-55
0.00
1.30
0.52
0.39
0.44
1.34
0.17
0.15
0.36
Flag
-100
GAPDH
-35

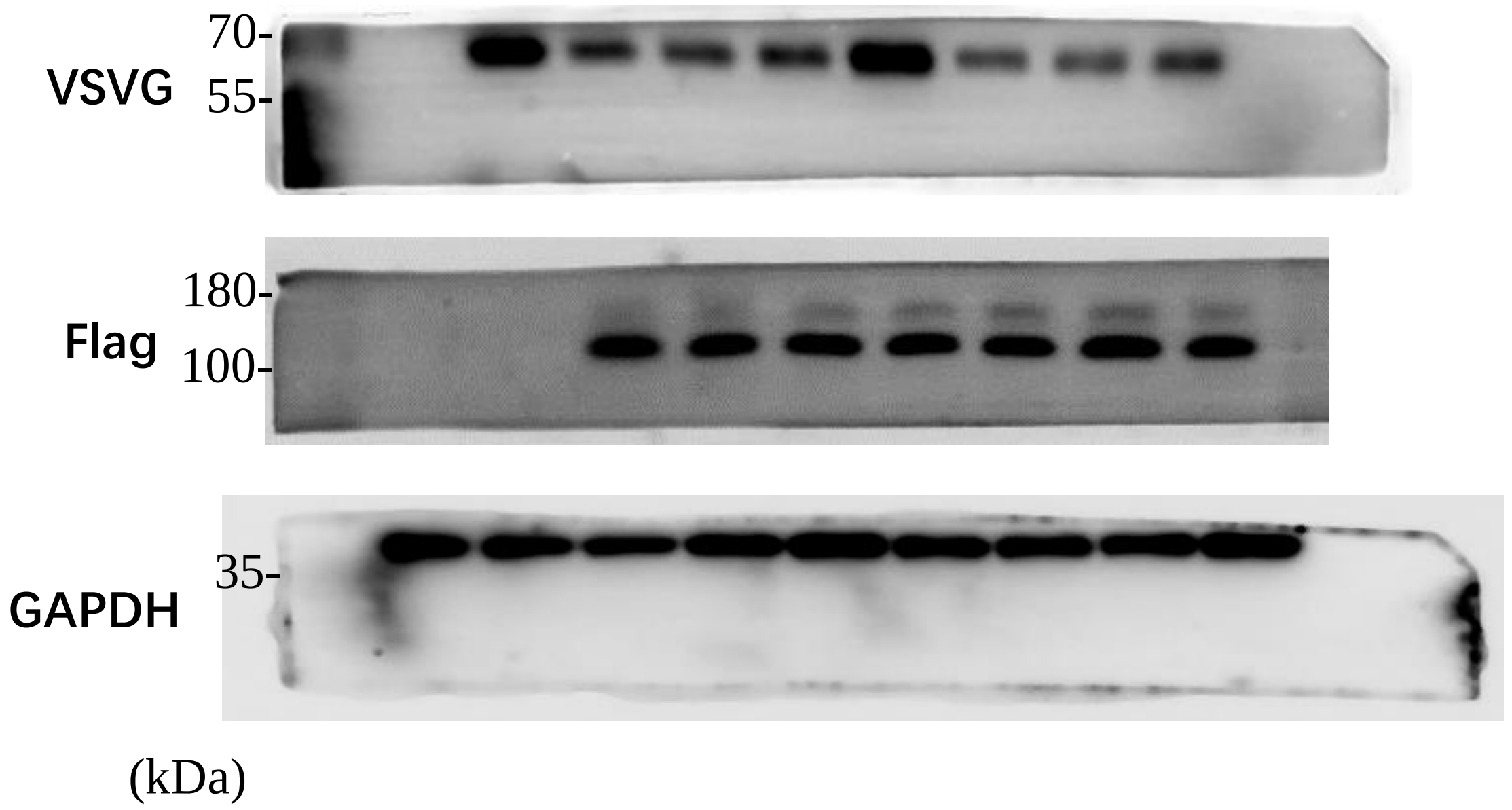
VSVG
70-
55-
Flag
180-
100-
GAPDH
35-
(kDa)

Fig. S6C

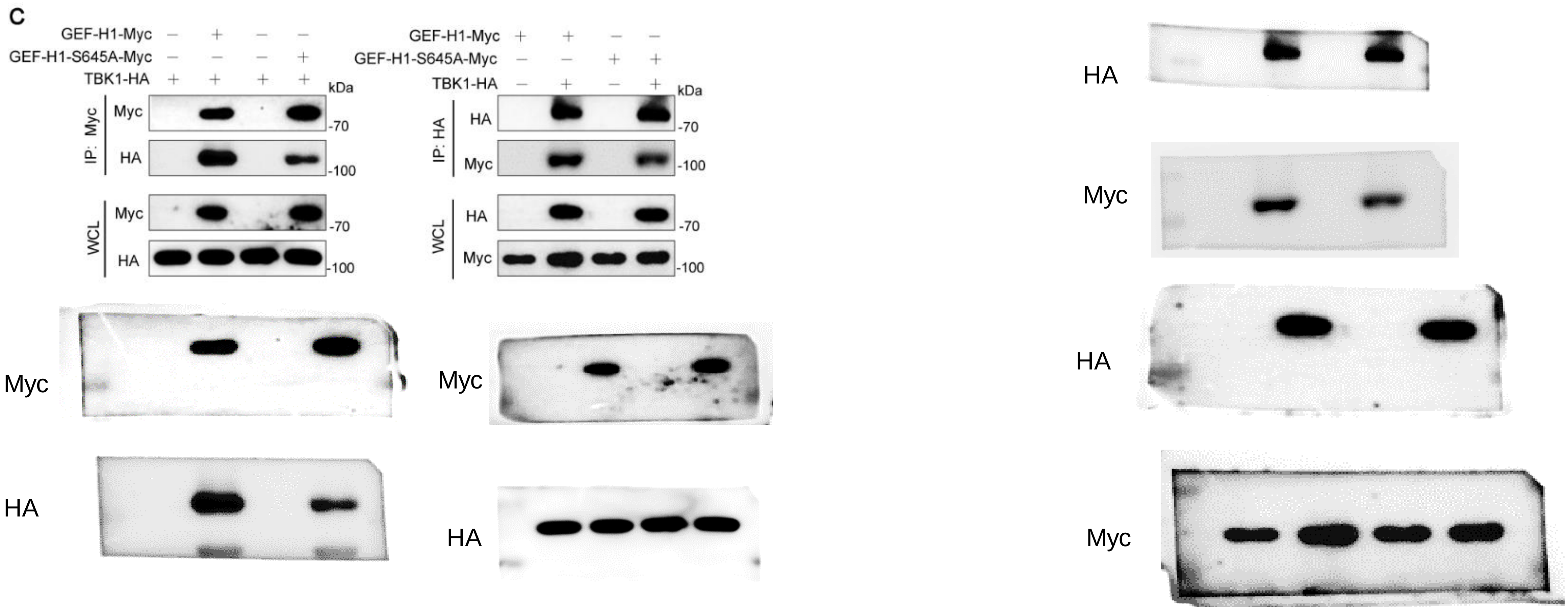

C
GEF-H1-Myc
GEF-H1-S645A-Myc
TBK1-HA
kDa
IP: Myc
Myc
HA
-70
-100
WCL
Myc
HA
-70
-100
GEF-H1-Myc
GEF-H1-S645A-Myc
TBK1-HA
kDa
IP: HA
HA
Myc
-70
-100
WCL
HA
Myc
-70
-100
Myc
HA
Myc
HA
HA
Myc
HA
Myc

Fig. S6E

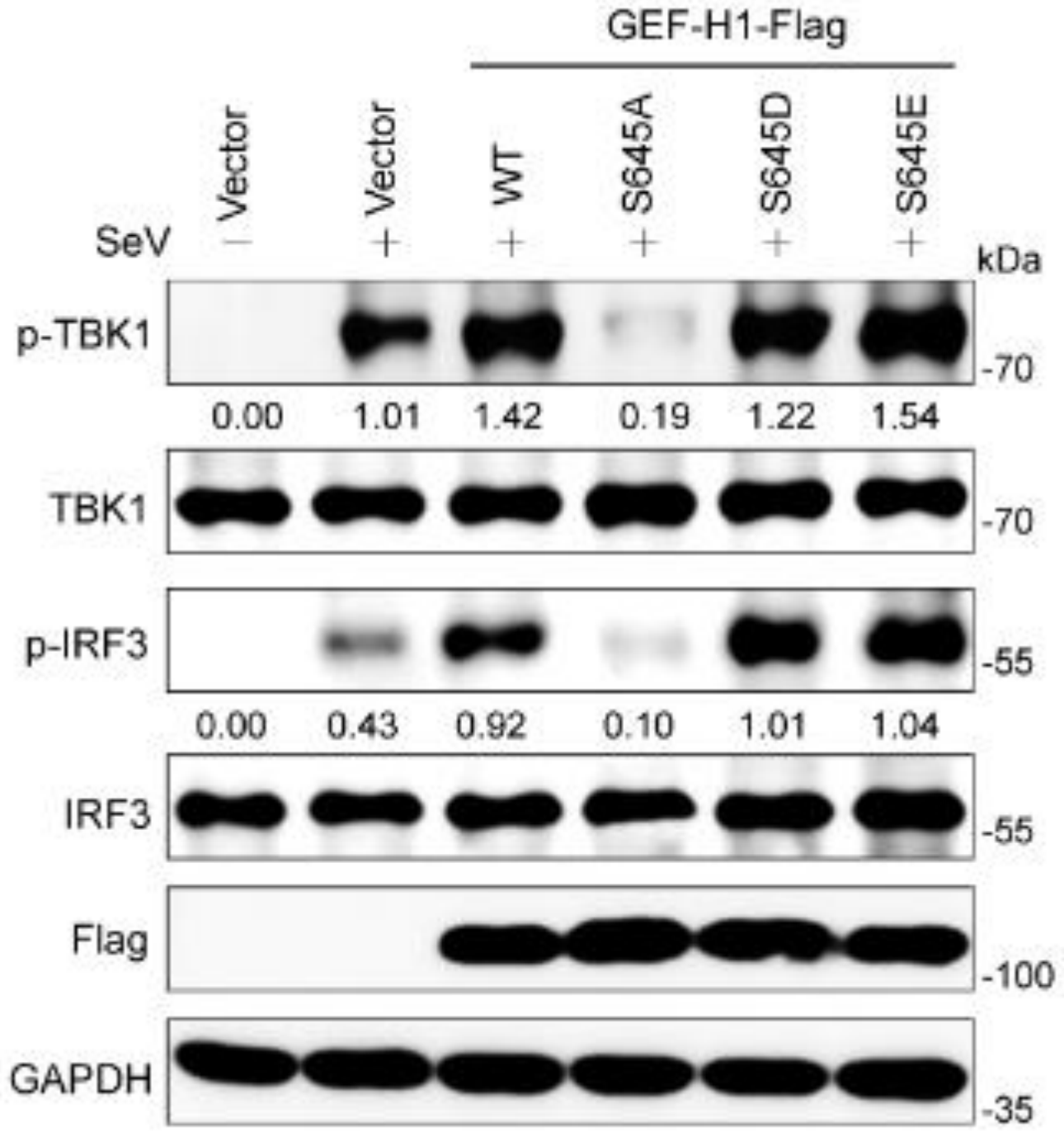
GEF-H1-Flag
SeV
Vector
Vector
WT
S645A
S645D
S645E
kDa
p-TBK1
-70
0.00 1.01 1.42 0.19 1.22 1.54
TBK1
-70
p-IRF3
-55
0.00 0.43 0.92 0.10 1.01 1.04
IRF3
-55
Flag
-100
GAPDH
-35

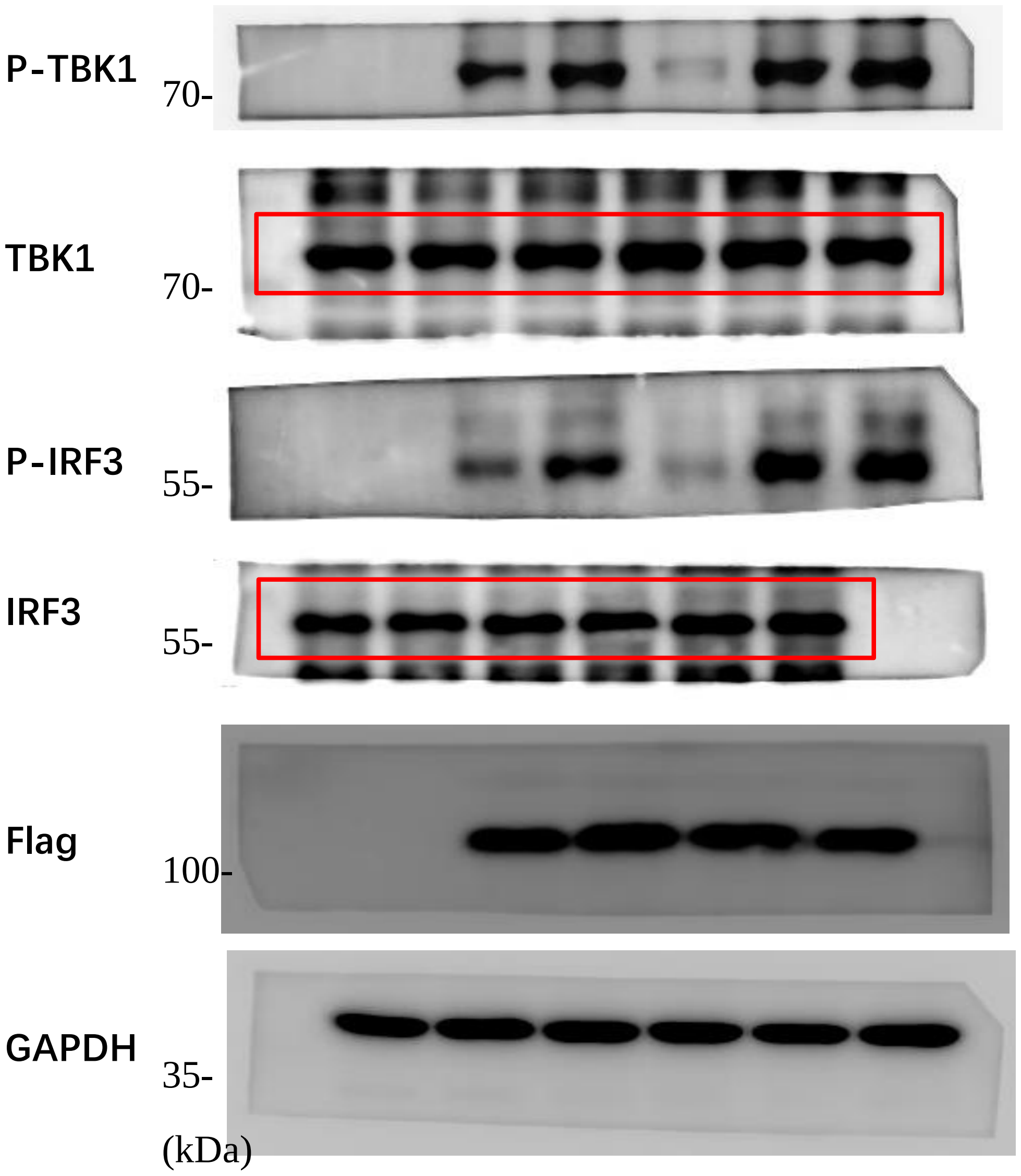
P-TBK1
70-
TBK1
70-
P-IRF3
55-
IRF3
55-
Flag
100-
GAPDH
35-
(kDa)

Fig. S6G

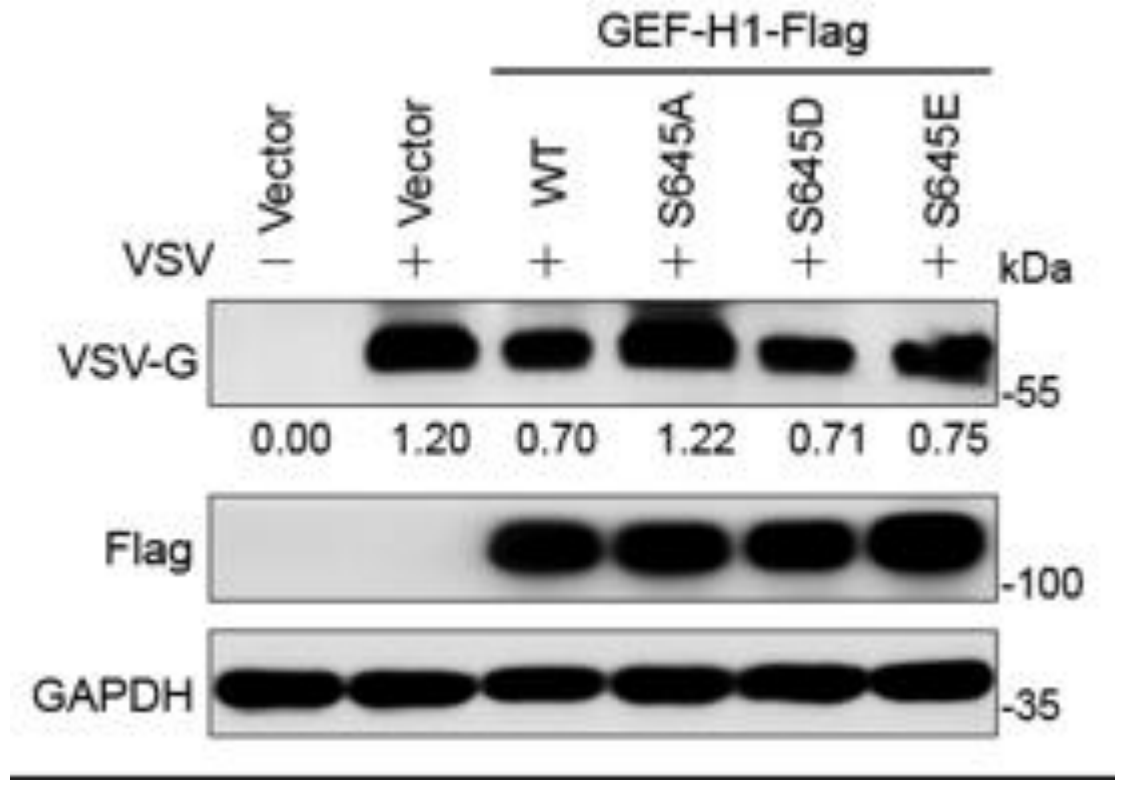

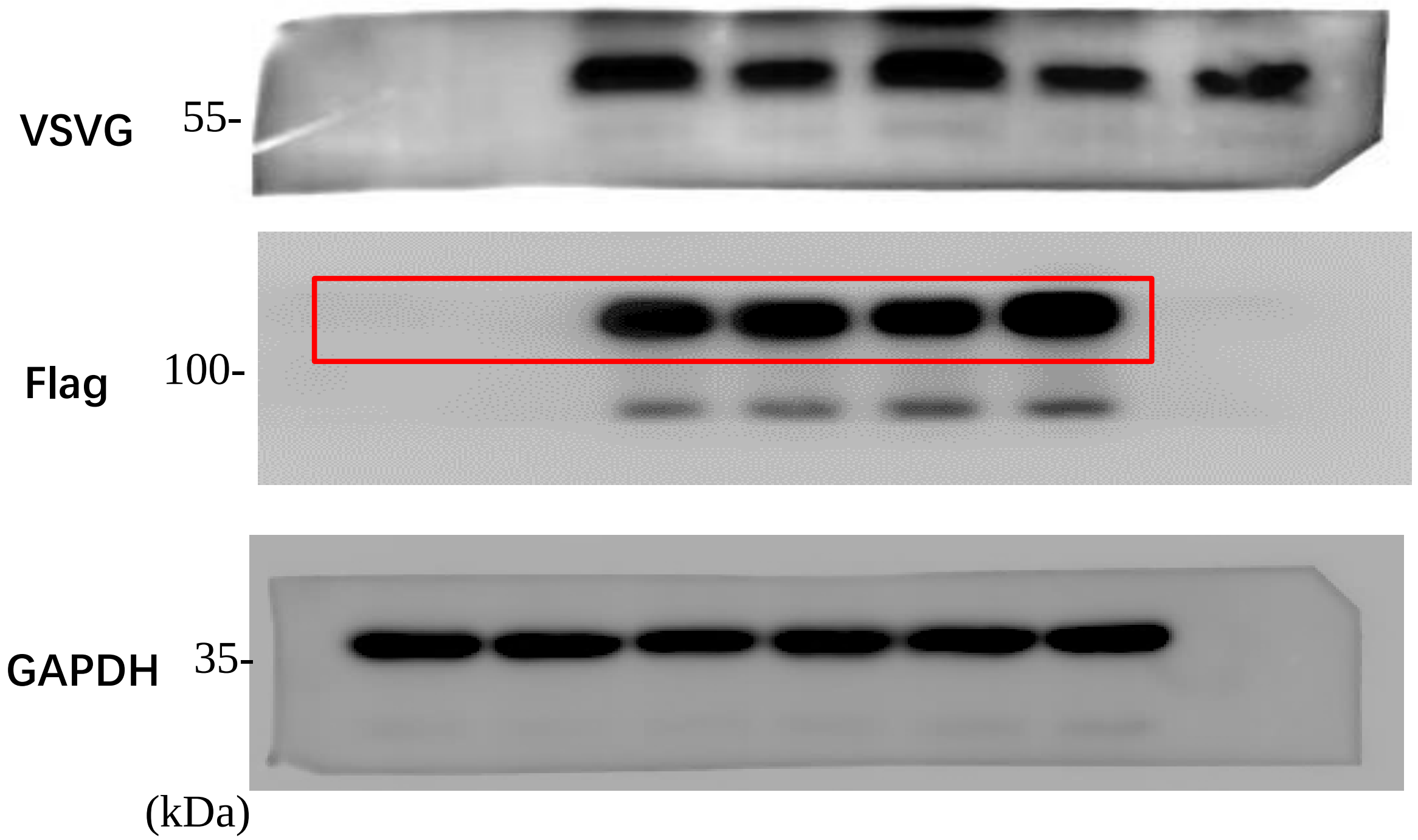

Fig. S6H

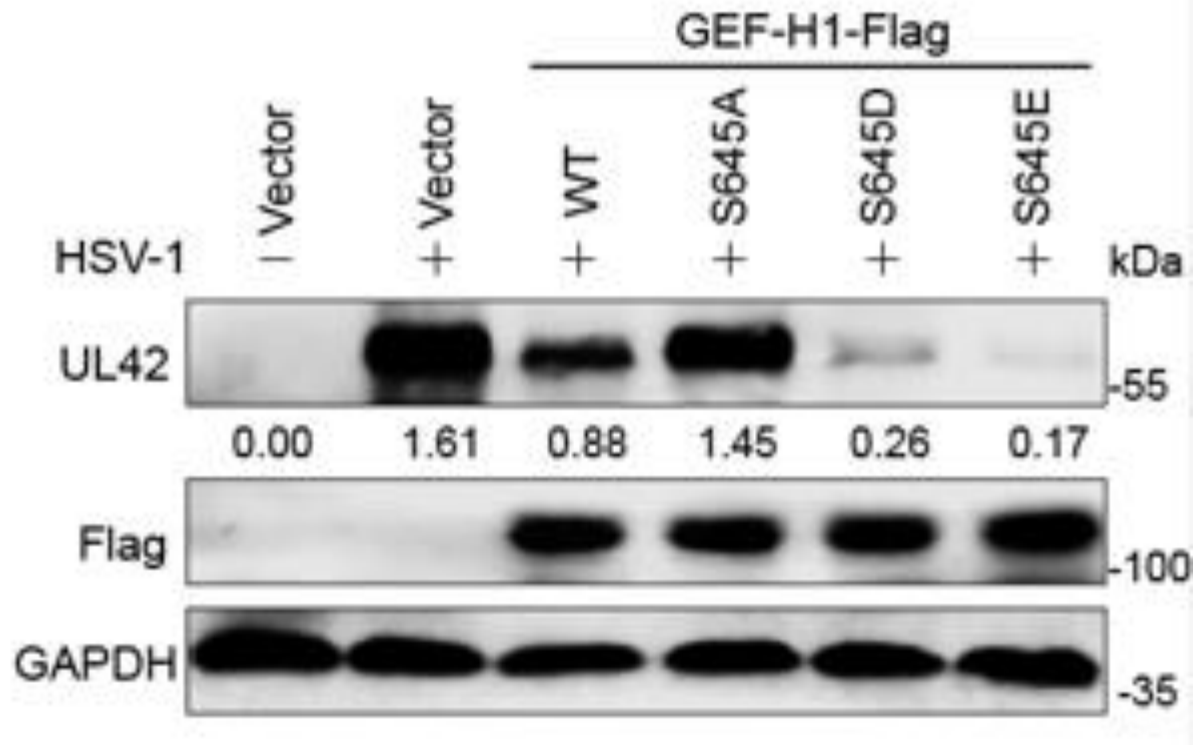

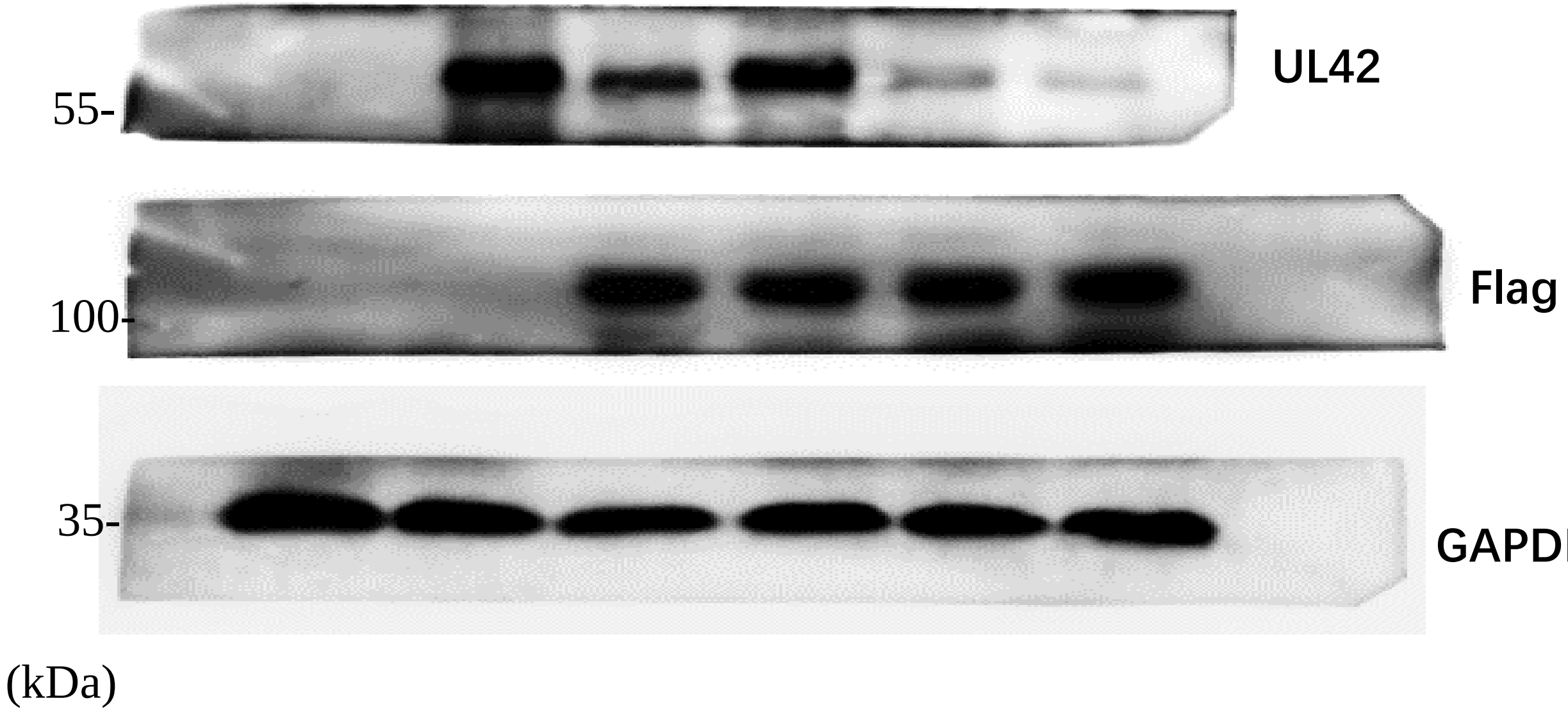

Supplement: Supplementary file 1 [file ppat.1014701.s009.pdf]
